# Detecting change in comparison to peers in NHS prescribing data: a novel application of cumulative sum methodology

**DOI:** 10.1186/s12911-018-0642-6

**Published:** 2018-07-09

**Authors:** Alex J. Walker, Seb Bacon, Richard Croker, Ben Goldacre

**Affiliations:** Centre for Evidence-Based Medicine, Nuffield Department of Primary Care Health Sciences, University of Oxford, Radcliffe Observatory Quarter, Woodstock Road, Oxford, OX2 6GG UK

**Keywords:** Prescribing data, CUSUM, Change detection

## Abstract

**Background:**

The widely used OpenPrescribing.net service provides standard measures which compare prescribing of Clinical Commissioning Groups (CCGs) and English General Practices against that of their peers. Detecting changes in prescribing behaviour compared with peers can help identify missed opportunities for medicines optimisation. Automating the process of detecting these changes is necessary due to the volume of data, but challenging due to variation in prescribing volume for different measures and locations. We set out to develop and implement a method of detecting change on all individual prescribing measures, in order to notify CCGs and practices of such changes in a timely manner.

**Methods:**

We used the statistical process control method CUSUM to detect prescribing behaviour changes in relation to population trends for the individual standard measures on OpenPrescribing. Increases and decreases in percentile were detected separately, using a multiple of standard deviation as the threshold for detecting change. The algorithm was modified to continue re-triggering when trajectory persists. It was deployed, user-tested, and summary statistics generated on the number of alerts by CCG and practice.

**Results:**

The algorithm detected changes in prescribing for 32 prespecified measures, across a wide range of CCG and practice sizes. Across the 209 English CCGs, a mean of 2.5 increase and 2.4 decrease alerts were triggered per CCG, per month. For the 7578 practices, a mean of 1.3 increase and 1.4 decrease alerts were triggered per practice, per month.

**Conclusions:**

The CUSUM method appears to effectively discriminate between random noise and sustained change in prescribing behaviour. This method aims to allow practices and CCGs to be informed of important changes quickly, with a view to improve their prescribing behaviour. The number of alerts triggered for CCGs and practices appears to be appropriate. Prescribing behaviour after users are alerted to changes will be monitored in order to assess the impact of these alerts.

**Electronic supplementary material:**

The online version of this article (10.1186/s12911-018-0642-6) contains supplementary material, which is available to authorized users.

## Background

There is an extensive literature documenting variation in care detected in routine electronic health record data, and efforts to distinguish warranted from unwarranted variation, as well as real change from statistical noise. There is evidence that audit and feedback strategies can be effective in improving prescribing behaviour, including a Cochrane review [1] and recent randomised controlled trials [2–4]. We run the OpenPrescribing.net service [5] which provides a user-friendly interface for the raw data on all National Health Service (NHS) prescribing in English primary care published by NHS Digital [6]. OpenPrescribing is freely available to anyone who wishes to use it and is widely accessed, with over 47,000 unique users during 2016. We regularly receive feedback from GPs, medicines optimisation teams and other researchers. The service presents various prescribing measures which have been developed by clinicians and pharmacists working in collaboration with data analysts to address issues of cost, safety or efficacy. Each month the performance of each Clinical Commissioning Group (CCG) and practice on each measure is calculated and presented in comparison with the whole population in the form of absolute figures and time-trends of centile performance. CCGs are clinically-led organisations responsible for the planning and commissioning of health care services for their local area. Change can be seen over time for many of these measures, as changes in prescribing behaviour occur in response to changes in price, evidence of effectiveness, or safety issues. Within these population trends, some clinicians or institutions change their behaviour sooner than others.

Statistical Process Control (SPC) is a range of techniques used to identify outliers and detect change in performance. It was originally developed for engineering applications such as monitoring manufacturing processes [7], but has since been applied to a wide range of other applications including medicine [8–10]. While potentially useful, it is thought to be underused in a medical context [11]. Cumulative sum control charts (CUSUM) calculate the cumulative deviation from the mean over an extended period and can therefore be used to detect slow but salient changes in performance. There are various implementations of the CUSUM methodology, and it can be adapted somewhat to suit different applications; it is also easy to adjust how sensitive the algorithm is to change.

OpenPrescribing has a monthly alerts service to practitioners, which notifies when there are signals suggesting variation in care that may benefit from clinician attention. These are initially triggered simply if a practice is an outlier (highest or lowest decile) in the most recent month’s data for a given numerator and denominator of prescribing data (see methods for a description of prescribing measures). Rather than just waiting for a given centre to enter the top or bottom decile, a useful addition would be to automatically detect and alert users to changes against population trends for any of the measures on OpenPrescribing.net, in order that they can respond earlier to a change. Given the lack of an established method of doing this automatically, and the number of practices and measures (making it impossible to achieve manually) we set out to apply Statistical Process Control techniques to the problem (specifically the CUSUM algorithm). In a conventional implementation of CUSUM, an alert is triggered once when change is detected, after which the algorithm resets, meaning that even if prescribing continues to decline, an alert is unlikely to be to be triggered for a few months. OpenPrescribing is open to all users, who may commence monitoring at different time points, where they would benefit from being informed of an ongoing change; furthermore, CCG or practice staff may benefit from repeated alerts where worsening performance is ongoing. We therefore also set out to adapt the methodology to ensure that alerts are triggered repeatedly where change continues to occur.

## Methods

### Data

We used data from the OpenPrescribing project, which imports prescribing data from the monthly prescribing data files published by NHS digital [6]. These contain data on cost and volume prescribed for each drug, dose and preparation, for each English general practice. These data are combined with practice list sizes, and British National Formulary (BNF) codes and names from the NHS Business Service Authority’s Information Portal [12]. OpenPrescribing uses these data to create tools including national trends in prescribing, pre-specified prescribing measures for CCGs and practices, and user-generated analyses on any combination of drugs or population denominators.

The prespecified prescribing measures have been developed to address issues of cost, safety or efficacy by clinicians and pharmacists working in collaboration with data analysts. Each month, OpenPrescribing calculates the percentile that each CCG and practice is in, for each measure. Measures are oriented such that a higher percentile corresponds to what would be considered ‘worse’ prescribing (with the exception of those where no value judgement is made, e.g. direct acting oral anticoagulants (DOACs) [13] and pregabalin [14]). Rather than using prescriptions per head of population these measures are created with prescribing volume for a set of drugs as the denominator, and a subset of those drugs as the numerator, in order to correct for population variation. For example, one measure assesses cost-effectiveness of prescribing on desogestrel [15], a commonly used oral contraceptive. This molecule is prescribed in various forms: Cerazette, an expensive branded package, and desogestrel, a cheap generic available after patent expiry of Cerazette in 2013. Current best practice is to prescribe low-cost generic desogestrel. The measure takes “branded desogestrel” as the numerator, and “all desogestrel” as the denominator, rather than practice population, in order to correct for population use of desogestrel.

**Fig. 1:**
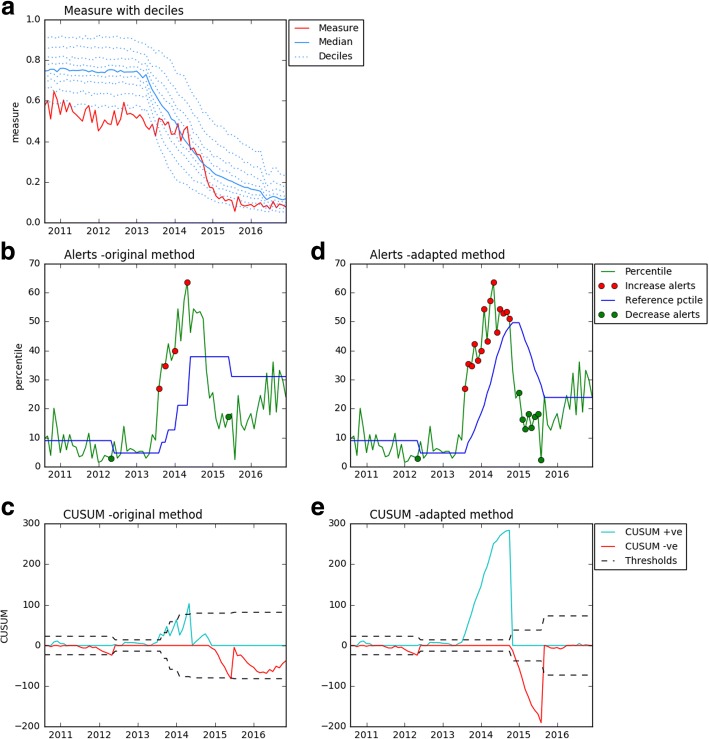
Graphs showing an example measure used to develop the alerts. Showing: **a** the measure as on openprescribing.net: Cerazette vs. desogestrel in 05D, **b** the percentiles with alerts highlighted for the standard method, **c** the cumulative sum, with threshold values for the standard method, **d** the percentiles with alerts highlighted for the continuing change method, **e** the cumulative sum, with threshold values for the continuing change method. Increase alerts all lie above the reference percentile line, while decrease alerts all lie below it

This desogestrel measure demonstrates the value of implementing statistical process control to identify change during periods of transition in practice. Over time, there is a clear trend towards Cerazette falling in comparison with all desogestrel (Fig. 1a). Some practices and CCGs enacted this change in practice faster than others, meaning a practice with previously good performance can change percentile without changing their prescribing practice, because the prescribing behaviour of the population changes around them. Triggering prescribing behaviour alerts to practices or CCGs on the basis of a crude analysis - for example, that they had entered the top 10% for this prescribing measure - would fail to capture this dramatic shift in comparison to peers, and fail to give timely feedback on performance.

### Development and CUSUM implementation

The desogestrel measure was used to develop the prototype for the alerts. Typically the development of thresholds for an implementation of CUSUM is iterative, according to constraints set by the user around the desired frequency of alerts. For our use-case manual setting of thresholds was not possible, due to large variations in noise, caused by large variations in prescribing frequency between different measures and locations. We therefore derived thresholds from standard deviation using the method described below.

We took our CUSUM methodology from that described by Montgomery [16] and slightly adapted it to fit our needs; here we show results from both the standard and adapted (henceforth referred to as “continuing change”) methods. This version uses a two sided CUSUM value (C^+^ and C^−^) which can generate either increase or decrease alerts. Firstly the mean percentile (reference mean, μ_0_) is calculated over the first 12 months, along with the standard deviation for that mean. Then the positive and negative cumulative deviations (C^+^ and C^−^) from the mean are calculated, according to the formula:

$$ {C}_i^{+}=\max \left[0,{x}_i-\left({\mu}_0+K\right)+{C}_{i-1}^{+}\right] $$

$$ {C}_i^{-}=\min \left[0,{x}_i-\left({\mu}_0-K\right)+{C}_{i-1}^{-}\right] $$

Where C_0_ = 0, x_i_ is each monthly observation, K is the ‘allowance’ or ‘slack’ value, which allows values to deviate a small amount without triggering an alert, and is calculated as 0.5x standard deviation). The value for C is calculated over consecutive months until a threshold value (H) is reached, calculated as 5x standard deviation. The multiplier for the threshold value (H) was initially recommended by Montgomery [16], and was finally determined iteratively in collaboration with our clinical team, according to whether detected changes were considered appropriate. At this point an alert is triggered. Then, for the “standard method”: the C value is reset to 0, and the reference mean and standard deviation are calculated as that of the *preceding* 12 months. As this suppressed repeated alerts after an alert is first triggered, we also developed a “continuing change method” for when change persists. The reference mean is re-calculated over the *preceding* 12 months. Then, if the C value continues to increase in relation to the new reference mean, another alert is triggered and the reference mean is again reset to the preceding 12 months. This continues until the C value stops increasing, after which the process is reset as per the standard method. The algorithm was generated in the programming language Python, which also runs the OpenPrescribing website, and then run against live data through the Application Programming Interface (API) of the service [17].

Any months of missing percentile data (usually due to the denominator being 0), were dropped from the analysis. Where there are insufficient data to run the algorithm, no alerts are triggered.

### Summary statistics on alert frequency

To permit visual comparison of alert triggers against underlying trend data we generated an example of each prespecified prescribing measure on openprescribing.net, for one randomly selected CCG (05Y [18]) and practice (G85138 [19]), Additional file 1: Appendix B. We also ran the algorithm for all measures, on all practices and CCGs, and summarised alerts triggered in the last month of available data (November 2016) to check for an appropriate level of alerting. Example CCGs and practices are referred to by national identifier rather than name as they were chosen arbitrarily, and are of no specific clinical interest.

### Reproducibility and code

All data analysis was performed using Python. Code is available in Additional file 1: Appendix A; available online alongside as a Jupyter notebook with data on Github [20]; and shared under an MIT License free for reuse with attribution. All underlying data is shared at FigShare [21]; through NHS Digital [6]; and though the API at OpenPrescribing.net [17].

## Results

### Development example

For our test measure (Cerazette vs. desogestrel) we successfully ran our algorithm on all 209 English CCGs and all but 24 (0.3%) of the 7554 practices. Inability to run the algorithm, was solely due to insufficient data points, where percentiles were missing because the denominator was 0. Figure 1 shows an example of a CCG for the Cerazette vs desogestrel measure. The analysis is shown for both the standard and continuing change methods. In this example, change in percentile initially occurs largely due to change in the population, then subsequently occurs due to change in the individual CCGs prescribing behaviour.

In Fig. 1, the mean percentile over the first 12 months was 8.9%. Initially the algorithm detects a decrease in May 2012, when the CCG is in the 3rd percentile. In August 2013, an increase is detected with both methods, after the percentile has climbed steeply to the 27th. For the standard method (Fig. 1b and c), there are 3 subsequent increases detected over the next 12 months. The continuing change method (Fig. 1 d and e) shows its utility in that it continues to trigger alerts for as long as the change continues to occur, in relation to the previous 12 months. After the increase alerts stop at around the 53rd percentile, for the standard method, a decrease is detected in June 2015, at the 17 h percentile. For the continuing change method this decrease is detected five months earlier, at the 25th percentile and continues for eight consecutive months, until the percentile is close to that of the previous 12 months. In this example, without the use of a change detection method such as this, the CCG in question would not have been notified of the change in prescribing of its peers, aside from when its prescribing is in the lowest (best) decile.

### Additional examples

**Fig. 2:**
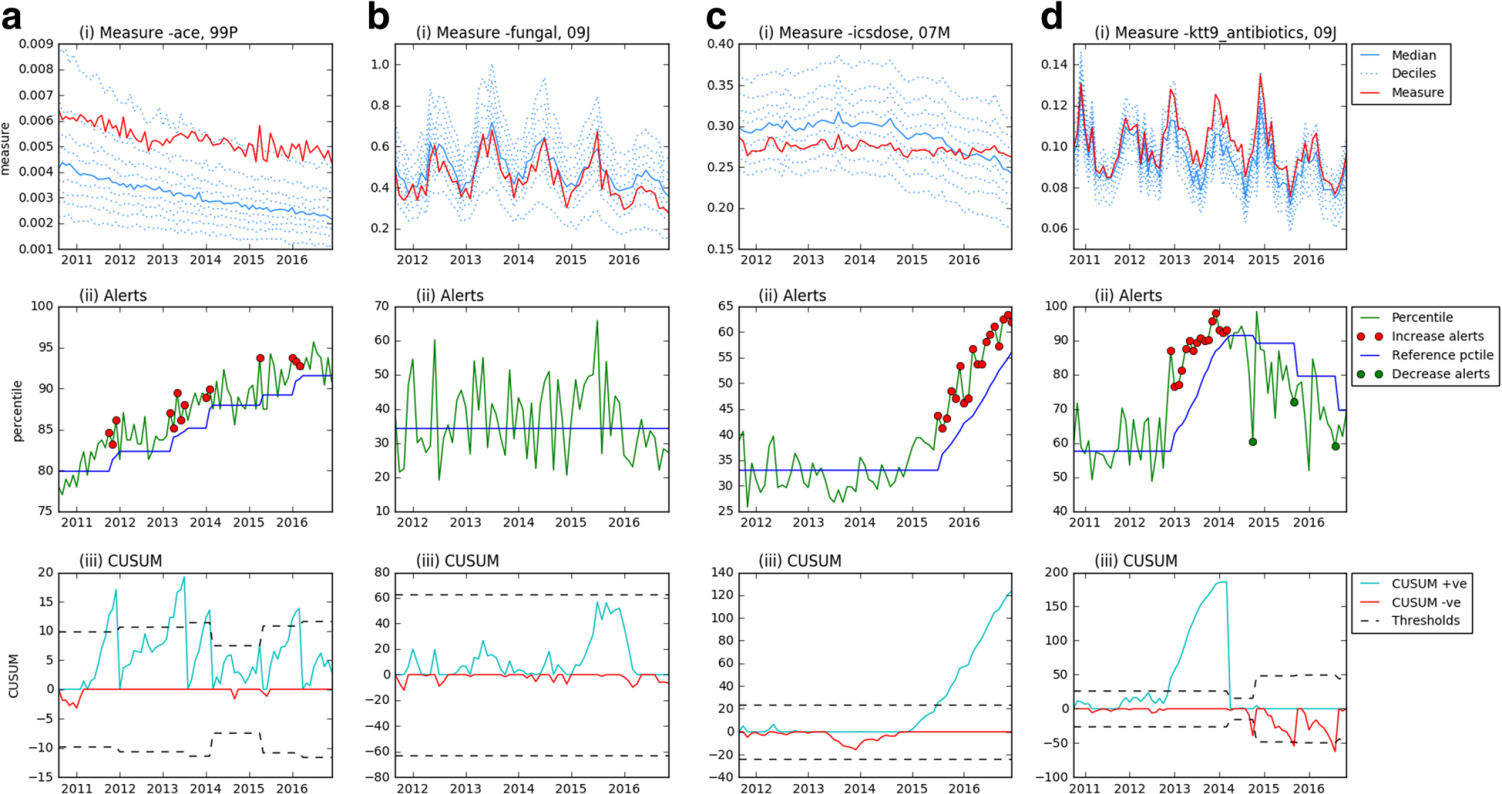
CCG examples. Graphs showing the measures for CCGs as shown on openprescribing.net (i), percentiles with alerts highlighted (ii) and the cumulative sum, with threshold values (iii). Examples are: **a** high-cost ace inhibitors in 99P, **b** topical treatment of fungal nail infections in 09 J, **c** high dose inhaled corticosteroids in 07 M, **d** antibiotic stewardship: volume of antibiotic prescribing (KTT9) in 09 J. Increase alerts all lie above the reference percentile line, while decrease alerts all lie below it

**Fig. 3:**
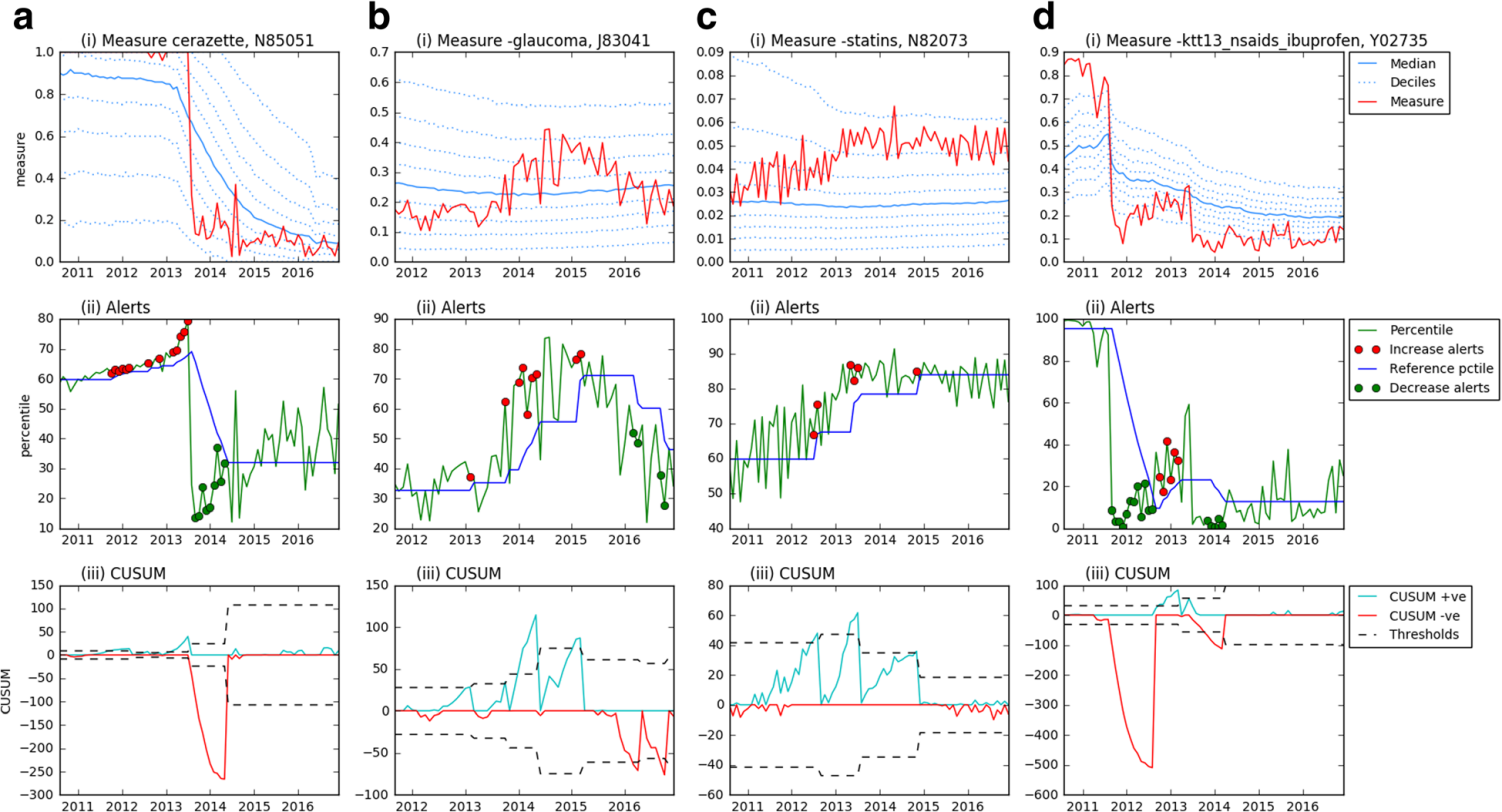
Practice examples. Graphs showing the measures for practices as shown on openprescribing.net (i), percentiles with alerts highlighted (ii) and the cumulative sum, with threshold values (iii). Examples are: **a** Cerazette vs. desogestrel in N85051, **b** glaucoma eye drops prescribed by brand in J83041, **c** high-cost statins in N82073, **d** non-preferred NSAIDs and COX-2 inhibitors (KTT13) in Y02735. Increase alerts all lie above the reference percentile line, while decrease alerts all lie below it

Figures 2 and 3 contain eight further examples of the change detection algorithm, four each for CCGs and practices. These contain a variety of examples including those where alerts are triggered continuously for a change that occurs gradually over a year or more (e.g. Fig. 2c), where change happens within a month or two (Fig. 3a), where an increase is detected, then later a decrease (Fig. 2d) and where no changes are detected (Fig. 2b). For measures that exhibit seasonal variation, such as Figs. 2b and d, this variation is effectively controlled for by using the percentile to determine alerts, assuming that the CCG/practice in question’s prescribing follows a similar seasonal trend.

Further examples are given in Additional file 1: Appendix B (and on Github [20]), where the CUSUM algorithm was run on all measures for a randomly selected CCG and practice (05Y and G85138). Within these results there is substantial heterogeneity in the amount of change that occurs, in the level of noise between different measures and between the CCG and practice, allowing visual comparison of raw data against alerts triggered in a wide range of settings.

### Summary statistics

**Fig. 4:**
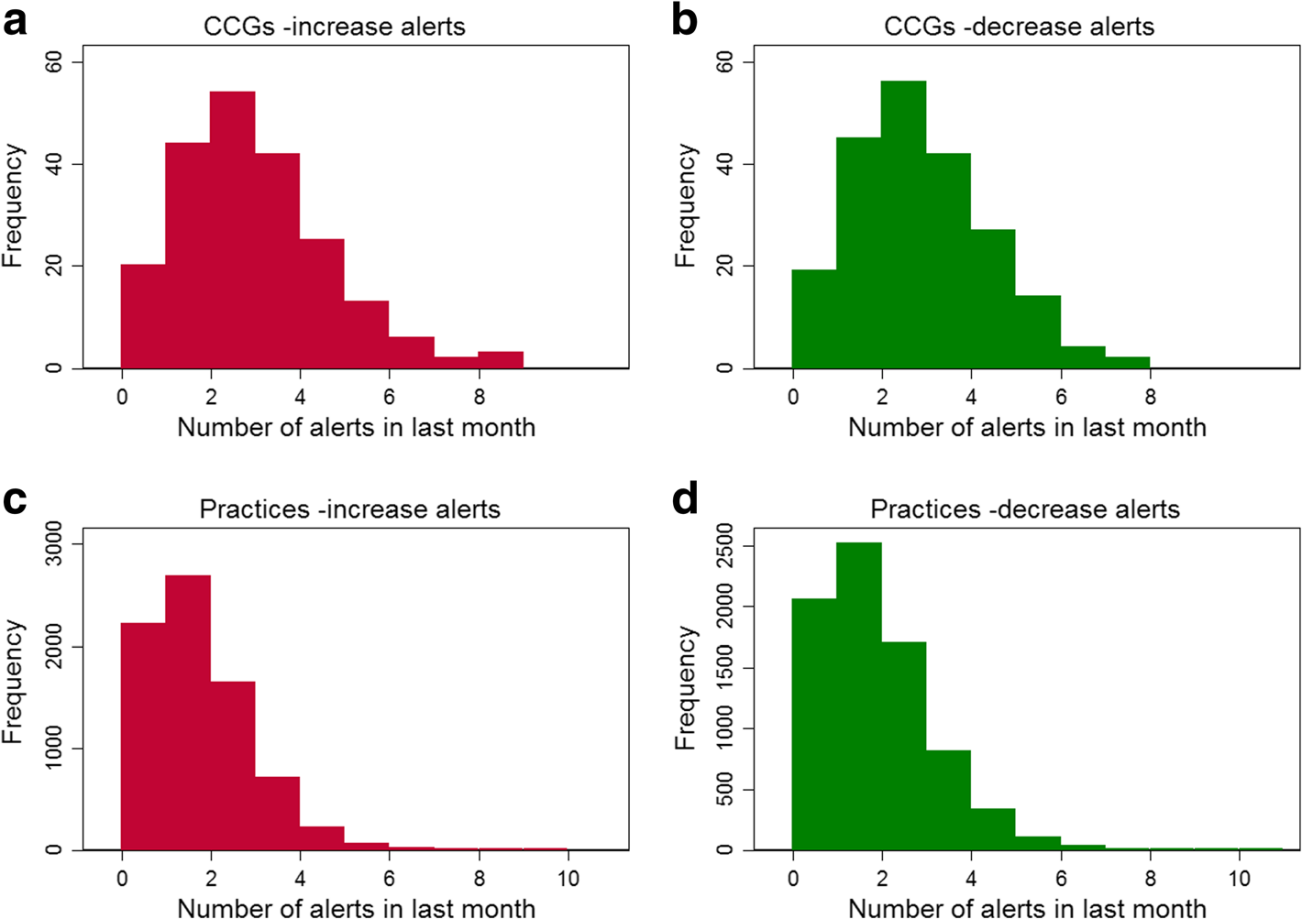
Histograms showing the distribution of the number of alerts received by each CCG (**a** and **b**) and practice (**c** and **d**) in the last month

Across the most recent month of data (November 2016) a mean of 4.9 changes were detected in CCGs, and 2.7 for practices. Figure 4 shows the number of increase and decrease alerts for both CCGs and practices. Table 1 shows the proportion of CCGs and practices where a change was detected, for each measure.

**Table 1:** Percentage of institutions receiving alerts, by measure

	CCGs		Practices	
Measure	Increase alerts, % last month (mean)	Decrease alerts, % last month (mean)	Increase alerts, % last month (mean)	Decrease alerts, % last month (mean)
**Mean alerts per month per CCG/practice**	**(2.5)**	**(2.4)**	**(1.3)**	**(1.4)**
**Mean %, all measures**	**7.7**	**7.5**	**3.9**	**4.3**
Topical treatment of fungal nail infections	5.3	6.2	2.1	2.9
High-cost PPIs	7.2	7.2	3.1	3.9
High cost tramadol preparations	2.4	3.3	4.6	4.4
High-cost drugs for erectile dysfunction	6.2	6.7	3.7	4.4
High-cost ARBs	6.7	4.3	2.7	2.7
High dose inhaled corticosteroids	14.4	12.9	5.8	5.1
Prescribing of dipyridamole	7.7	7.7	5.0	5.0
Methotrexate 10 mg tablets	5.3	7.7	2.7	3.1
Vitamin B complex	16.3	8.6	5.0	5.0
Soluble/effervescent forms of paracetamol and co-codamol	6.7	3.3	4.0	4.2
Non-preferred NSAIDs and COX-2 inhibitors	2.9	4.3	3.4	4.4
Other lipid-modifying drugs	8.1	6.7	4.7	3.9
Direct Oral Anticoagulants (DOACs)	14.4	15.3	4.5	4.9
Silver dressings	5.3	5.7	2.8	3.2
Long-acting insulin analogues	6.7	7.7	3.2	3.9
Volume of antibiotic prescribing	5.3	4.8	4.9	5.0
Prescribing of pregabalin	12.0	12.9	5.6	5.4
Diltiazem preparations prescribed generically	12.0	12.4	4.0	6.6
Co-proxamol	8.1	5.3	1.0	4.6
High dose opioids	4.8	6.2	5.8	5.5
Ciclosporin and tacrolimus oral preparations prescribed generically	2.9	5.7	1.4	2.2
Desogestrel prescribed as a branded product	11.5	12.4	4.6	6.2
Nebivolol 2.5mg tablets	7.2	6.7	3.1	2.6
Three-day antibiotic courses for UTIs	3.8	2.9	2.9	3.5
High-cost ACE inhibitors	2.4	2.4	1.7	3.0
High-cost statins	4.8	8.6	3.4	3.9
Glaucoma eye drops prescribed by brand	12.0	9.1	4.0	4.8
Higher dose Proton Pump Inhibitors	14.8	12.9	7.1	8.3
Co-amoxiclav, cephalosporins & quinolones	11.0	8.6	4.6	4.8
Extended-release quetiapine	11.5	7.7	6.2	5.9
Short acting beta agonist inhalers	3.8	8.6	3.5	3.0
Keppra vs. levetiracetam	5.3	4.3	3.3	2.7

Bold figures are summary numbers for all measures, non-bold are figures for each separate measure

## Discussion

### Summary

We have developed and implemented an adaptation of the CUSUM methodology to detect changes in prescribing for one CCG or practice, in relation to the whole population of CCGs or practices, across a wide range of prescribing measures. Our modification and implementation successfully met various specific requirements of our use case, as discussed below. The method was effective in detecting changes that we determined to be clinically important. Though we did not formally assess the utility and appropriateness of the alerts generated, we plan to assess their impact once sufficient follow-up data has been accrued.

### Strengths & weaknesses

Our modification and implementation of the CUSUM method meets various specific requirements of our use-case. Firstly, in contrast to standard Shewhart control charts [7, 9], the approach described here is able to detect small changes over a period of time that may still be clinically interesting. Secondly, by using a multiple of the standard deviation of the reference mean as the threshold value for detecting changes, the method is able to adapt to our diverse range of measures and across many CCGs and practices. This means that where the level of noise is especially high, the algorithm adjusts such that typical levels of noise do not trigger an alert. Conversely, where the variation in percentile is very low initially, an alert is triggered very quickly once a change occurs, to indicate atypical behaviour.

Thirdly, after an initial alert has been triggered our modification of the standard CUSUM implementation checks for continuing deviation from the mean over the preceding 12 months, and re-triggers an alert if such continued change is detected. This meets an important requirement on OpenPrescribing: the alerts service is open to any user, some of whom may sign up for alerts shortly after an initial trigger has been sent, and may not be aware of historic alerts. This confers the additional benefit of reminding CCGs or practices that do not respond to the initial alert that a change on a measure has both occurred and is ongoing. This adaptation also has the unintended benefit of sometimes selecting a more appropriate reference mean – often after the change has largely stopped – which then reduces the chance of unnecessary alerts being generated after the change has taken place. Another advantage of the approach that we have taken is that it is easy to modify the parameters of the CUSUM algorithm, in order to alter how sensitive it is to change. We set these parameters according to recommendations by Montgomery [16], and in our view, the algorithm triggered alerts at times that we considered clinically appropriate.

Through informal user testing (not reported here) and iteration, we think that an appropriate balance has been found in the level and suitability of alerting. An interesting point to note is that CCGs tended to have more detected changes than practices. This is likely due a higher level of statistical noise in practices, due to generally lower prescribing numbers. It is not necessarily a problem for CCGs to receive a higher volume of alerts, given that they often have a dedicated medicines optimisation team who can investigate alerts appropriately.

Occasionally, small changes in the percentile are detected as alerts. This occurs where the percentile is especially consistent and occurs more commonly at extreme percentiles, where the percentiles are more spaced out. However, such small changes in percentile can correspond to substantial absolute changes in prescribing. For example, for the example given in Fig. 1, between May and June 2016, the CCG moves from the 100th to the 99th percentiles, but this change corresponds to a change from 62.2 to 34.8% in the proportion of Cerazette prescribing. It is therefore not useful to set universal limits for the size of percentile change that should trigger an alert.

In a few cases, the algorithm detects a change in a somewhat arbitrary place (e.g. high-cost ACE inhibitors for CCG 05Y in Additional file 1: Appendix A). This is possible when the level of noise within the percentiles changes over time. For example, if the level of noise is low initially, a low trigger threshold will be set, if the noise then increases (perhaps due to a reduction in overall prescribing for that measure), this may occasionally trigger an alert when there is no underlying shift in the measure. This also occurs where prescribing numbers are especially small (low single figure denominators. This is more common in small practices and can cause the percentile to change very erratically. Though this does not always trigger an inappropriate alert, there may be some utility in filtering out alerts where changes are detected based on very small numbers; we will consider and respond to user-feedback on this issue.

These examples highlight some potential pitfalls in applying the same method to a diverse array of data, but do not negate the utility of these methods; rather they emphasize the need for users to investigate alerts individually. Indeed, these limitations are mostly restricted to situations where the underlying data are not sufficient to make a meaningful judgement about a CCG or practice’s prescribing, even with careful clinical consideration. Given the lack of formal testing here, it is currently left to the reader and user to determine how useful the generated alerts are. Here we set out to describe the development of the method, such that users can understand how alerts are generated and that others may use the same implementation.

### Context of other findings

There are many examples of the use of SPC, and even CUSUM in medicine. The most comparable study that we know of [22] used similar prescribing data and used the CUSUM methodology to detect a change of one clinical entity in relation to others in the local area, for a prespecified prescribing intervention. This is a good initial demonstration of the utility of CUSUM in detecting changes against background noise. We go further by creating an automated tool that is effective across many diverse prescribing measures, and diverse sizes of centre, across the health service of a whole country.

Additionally, SPC is being used increasingly in medical research generally. For example, for monitoring surgical outcomes [23–25], monitoring emergency medical outcomes [26] and even monitoring physiological response to antihypertensive treatments [27]. These different studies have used various different CUSUM implementations (summarised in [28, 29]) according to their different needs.

We used a two-sided implementation as described by Montgomery [16] because we are interested in notifying practices when their prescribing behaviour changes in either direction. We do not know of any other studies that have used our retriggering adaptation, where we determine whether an increase is persistently occurring. However, the adaptation bears some mathematical resemblance to the manner in which the V-mask CUSUM method is calculated [30]. Other adaptations to the CUSUM method are unlikely to be useful for our needs. For example, Novick et al. [24] compare a risk adjusted CUSUM implementation to an unadjusted one. The risk adjustment is used in this case to correct for the baseline risk changing over time in surgical outcomes. Additionally, a Bernoulli CUSUM can be used for situations where a binary outcome is being measured [31]. Though the prescribing measures used here could be described in terms of binary prescribing choices, we believe that it is simpler and more elegant to use the percentile for our needs.

### Policy implications and further research

The intention of this implementation of the CUSUM algorithm is to notify interested users (i.e. those who subscribe to the alerts) of clinically important changes to their prescribing patterns in relation to the prescribing of peers. It is clear from the user testing that in order for the alerts to have the maximum positive impact, the manner in which they are communicated must be carefully considered. The user testing highlighted the need to communicate the size and duration of the change that has occurred along with the notification. Although we have considered detecting increase and decrease changes in the same way methodologically here, they clearly have different implications. A detected increase in percentile may (for most measures) highlight a need for action by the CCG or practice to bring prescribing back into line with their peers, whereas a detected decrease might indicate that a recent change that was made was effective in improving prescribing. There are two prescribing measures in the current set on OpenPrescribing (DOACs [13] and pregabalin [14]) where no value judgement is made over an increase or decrease in the measure, but change in relation to peers is noteworthy regardless, so these will be communicated in alerts differently to other measures. Additionally, while there are many examples of practices getting worse as defined by our measures, in some cases there are some legitimate underlying reasons for this. It is therefore important to stress that the alerts are intended as an initial signpost that something has changed, and it is important that each CCG, practice, or other user investigates any underlying reasons for a change identified.

There are two mechanisms for collecting further information on the impact and quality of this analytic approach. Firstly, within the OpenPrescribing project, prescribing behaviour can be monitored over time after changes are detected. As we know from the OpenPrescribing dataset who is receiving alerts and who has interacted with the emails in various ways (e.g. clicked links to investigate an alert further), we will be able to assess the impact of alerts by comparing the change in prescribing in the months after an alert by subscribing versus non-subscribing institutions. Secondly, this service is now generating alerts to users, and will shortly be presented on the OpenPrescribing “labs” page. We encourage users to review the triggering of alerts on a measure at any CCG/practice of interest and give feedback on whether they view the alerts and thresholds as clinically useful, or any other aspect of the OpenPrescribing project, by emailing feedback@openprescribing.net.

## Conclusions

We have developed and implemented an adaptation of the CUSUM methodology to detect changes across a range of measures of prescribing in NHS primary care. We will be refining the implementation and monitoring change in prescribing in response to these alerts.

## Additional file

Additional file 1: Appendix A. Code. Appendix B. Example graphs. (PDF 4946 kb)

## Abbreviations

BNF: British National Formulary
CCG: Clinical Commissioning Group
CUSUM: Cumulative sum
SPC: Statistical Process Control
