# Supplementary material for: Detecting change in comparison to peers in NHS prescribing data: a novel application of cumulative sum methodology
Source: BMC Med Inform Decis Mak. 2018 Jul 9;18:62. doi: 10.1186/s12911-018-0642-6 (PMC6038291; doi:10.1186/s12911-018-0642-6)

Detecting change in prescribing data using CUSUM

March 13, 2017

1 Appendix A - Code

1.1 Get data from Open Prescribing API

```
In [1]: import json
        import urllib2
        import numpy as np
        import matplotlib.pyplot as plt
        import datetime

        def get_percentiles_for(measure, location):
            percentiles = []
            dates = []
            calc_value = []
            #for CCGs
            if len(location) == 3:
                # Create a request to the API
                url = ('https://openprescribing.net/'
                   'api/1.0/measure_by_ccg/?format=json&measure=' + measure)
                request = urllib2.Request(url)

                # Pretend to be a real web browser
                request.add_header(
                    'User-Agent',
                    'Mozilla/5.0 (X11; U; Linux i686) '
                    'Gecko/20071127 Firefox/2.0.0.11')
                opener = urllib2.build_opener()
                response = opener.open(request).read()

                # Convert the JSON string into a python dictionary
                data = json.loads(response)

                # Extract the data about the CCG we're interested in
                for datum in data['measures'][0]['data']:
                    if datum['pct_id'] == location:
                        percentiles.append(datum['percentile'])
                        dates.append(datum['date'])
```

```
            calc_value.append(datum['calc_value'])
    #for practices:
    else:
        # Create a request to the API
        url = ('https://openprescribing.net/'
           'api/1.0/measure_by_practice/?format=json&org=' + location)
        request = urllib2.Request(url)

        # Pretend to be a real web browser
        request.add_header(
            'User-Agent',
            'Mozilla/5.0 (X11; U; Linux i686) '
            'Gecko/20071127 Firefox/2.0.0.11')
        opener = urllib2.build_opener()
        response = opener.open(request).read()

        # Convert the JSON string into a python dictionary
        data = json.loads(response)

        # Extract the data about the measure we're interested in
        for n in range(len(data['measures'])):
            if data['measures'][n]['id'] == measure:
                for datum in data['measures'][n]['data']:
                    #print datum
                    percentiles.append(datum['percentile'])
                    dates.append(datum['date'])
                    calc_value.append(datum['calc_value'])
    #convert missing percentile values from None to numpy nan
    percentiles2 = np.array(map(lambda x: np.nan
                                if x==None else x, percentiles))
    return {'percentiles':percentiles2,
            'dates':dates,
            'calc_value':calc_value}

def get_measures():
    measures = []
    url = ('https://openprescribing.net/'
       'api/1.0/measure_by_practice/?format=json&org=G82105')
    request = urllib2.Request(url)
    request.add_header(
        'User-Agent',
        'Mozilla/5.0 (X11; U; Linux i686) '
        'Gecko/20071127 Firefox/2.0.0.11')
    opener = urllib2.build_opener()
    response = opener.open(request).read()
    data = json.loads(response)

    # Extract the data about the measure we're interested in
```

```
        #for n in range(len(data['measures'])):
        for datum in data['measures']:
            #print datum
            measures.append(datum['id'])
        return measures
```

```
In [2]: def get_ccgs_practices():
            with open('Patient_List_Size_CCG_2016_09.csv') as csvfile:
                ccgs = []
                ccgs_import = csv.reader(csvfile, delimiter=',')
                for row in ccgs_import:
                    ccgs.append(row[0])
            with open('gp-reg-patients-prac-quin-age_2019_09.csv') as csvfile:
                practices = []
                practices_import = csv.reader(csvfile, delimiter=',')
                for row in practices_import:
                    practices.append(row[0])
            return {'ccgs':ccgs, 'practices':practices}
```

1.2 Import deciles from csv

```
In [8]: import csv
        def get_deciles(measure, location):
            with open('allmeasuresdeciles_12_16.csv') as csvfile:
                deciles_import = csv.reader(csvfile, delimiter=',')
                date = []
                d_10 = []
                d_20 = []
                d_30 = []
                d_40 = []
                d_50 = []
                d_60 = []
                d_70 = []
                d_80 = []
                d_90 = []
                for row in deciles_import:
                    if row[1] == measure:
                        date.append(row[0])
                        if len(location) == 3:
                            d_10.append(float(row[2]))
                            d_20.append(float(row[3]))
                            d_30.append(float(row[4]))
                            d_40.append(float(row[5]))
                            d_50.append(float(row[6]))
                            d_60.append(float(row[7]))
                            d_70.append(float(row[8]))
                            d_80.append(float(row[9]))
                            d_90.append(float(row[10]))
```

```
                    else:
                        d_10.append(float(row[11]))
                        d_20.append(float(row[12]))
                        d_30.append(float(row[13]))
                        d_40.append(float(row[14]))
                        d_50.append(float(row[15]))
                        d_60.append(float(row[16]))
                        d_70.append(float(row[17]))
                        d_80.append(float(row[18]))
                        d_90.append(float(row[19]))
            dates = [datetime.datetime.strptime(date,
                               '%d/%m/%Y').date() for date in date]
        return {'dates':dates,
                'd_10':d_10,'d_20':d_20,'d_30':d_30,
                'd_40':d_40,'d_50':d_50,'d_60':d_60,
                'd_70':d_70,'d_80':d_80,'d_90':d_90}
```

1.3 CUSUM algorithm

```
In [9]: def cusum(data,months_smoothing,sensitivity):
        """performs the CUSUM algorithm on input data string"""
        smax = [0]
        smin = [0]
        # set reference_percentile and threshold at start
        # & if threshold reached
        non_missing_percentiles = data[~np.isnan(data)]
        reference_percentile = [np.mean(non_missing_percentiles
                                        [0:months_smoothing])]
        threshold = [np.std(non_missing_percentiles[0:months_smoothing])
                     * sensitivity] # +remove nan values
        alert = []
        alert_percentile_pos = [None]
        alert_percentile_neg = [None]
        for i in range(1,len(data)):

            if smax[i-1] > threshold[i-1] or smin[i-1] < -threshold[i-1]:
                #generate temp smax/smin
                reference_percentile.append \
                        (np.mean(data[i-months_smoothing:i]
                        [~np.isnan(data[i-months_smoothing:i])]))
                smax_temp = (max(0, data[i] - (reference_percentile[i] +
                                            (0.5 * threshold[i-1] /
                                             sensitivity)) + smax[i-1]))
                smin_temp = (min(0, data[i] - (reference_percentile[i] -
                                            (0.5 * threshold[i-1] /
                                             sensitivity)) + smin[i-1]))

                #test whether change still occuring *IN THE SAME DIRECTION*
```

```
        ## positive change
        if smax[i-1] < smax_temp and smax[i-1] > threshold[i-1]:
            threshold.append(threshold[i-1])
            smax.append(smax_temp)
            smin.append(smin_temp)
            alert.append(i-1)
            alert_percentile_pos.append(data[i])
            alert_percentile_neg.append(None)

        ## negative change
        elif smin[i-1] > smin_temp and smin[i-1] < -threshold[i-1]:
            threshold.append(threshold[i-1])
            smax.append(smax_temp)
            smin.append(smin_temp)
            alert.append(i-1)
            alert_percentile_pos.append(None)
            alert_percentile_neg.append(data[i])

        ## if not, reset
        else:
            alert.append(i-1)
            threshold.append(np.std(data[i-months_smoothing:i]
                                [~np.isnan(data[i-months_smoothing:i])])
                                 * sensitivity) # +remove nan values
            #reset smax/smin to 0
            #modified to include the value for the current month
            smax.append(max(0, data[i] - (reference_percentile[i] +
                                          (0.5 * threshold[i] /
                                           sensitivity))))
            smin.append(min(0, data[i] - (reference_percentile[i] -
                                          (0.5 * threshold[i] /
                                           sensitivity))))
            alert_percentile_pos.append(None)
            alert_percentile_neg.append(None)

    #else append previous values
    else:
        reference_percentile.append(reference_percentile[i-1])
        threshold.append(threshold[i-1])

        #calculate smax/smin
        smax.append(max(0, data[i] - (reference_percentile[i] +
                                      (0.5 * threshold[i] /
                                       sensitivity)) + smax[i-1]))
        smin.append(min(0, data[i] - (reference_percentile[i] -
                                      (0.5 * threshold[i] /
                                       sensitivity)) + smin[i-1]))
```

```
                if smax[i] > threshold[i]:
                    alert_percentile_pos.append(data[i])
                    alert_percentile_neg.append(None)
                elif smin[i] < -threshold[i]:
                    alert_percentile_neg.append(data[i])
                    alert_percentile_pos.append(None)
                else:
                    alert_percentile_pos.append(None)
                    alert_percentile_neg.append(None)
        return {'smax':smax, 'smin':smin,
                'reference_percentile':reference_percentile,
                'threshold':threshold,
                'alert':alert,
                'alert_percentile_pos':alert_percentile_pos,
                'alert_percentile_neg':alert_percentile_neg}
```

1.4 Draw plots

```
In [10]: def draw_plots(cusum_out, percentiles, dates, deciles):
             """draws 3 plots containing measure, alerts and CUSUM"""
             #plot deciles and percentile values
             negthreshold = [ -x for x in cusum_out['threshold']]
             plt.figure(figsize=(5, 10))
             plt.subplot(311)
             plt.plot(deciles['dates'],deciles['d_10'],
                      color = '#298CFF', linestyle = ':')
             plt.plot(deciles['dates'],deciles['d_20'],
                      color = '#298CFF', linestyle = ':')
             plt.plot(deciles['dates'],deciles['d_30'],
                      color = '#298CFF', linestyle = ':')
             plt.plot(deciles['dates'],deciles['d_40'],
                      color = '#298CFF', linestyle = ':')
             plt.plot(deciles['dates'],deciles['d_50'],
                     color = '#298CFF', label = 'Median')
             plt.plot(deciles['dates'],deciles['d_60'],
                      color = '#298CFF', linestyle = ':')
             plt.plot(deciles['dates'],deciles['d_70'],
                      color = '#298CFF', linestyle = ':')
             plt.plot(deciles['dates'],deciles['d_80'],
                      color = '#298CFF', linestyle = ':')
             plt.plot(deciles['dates'],deciles['d_90'],
                      color = '#298CFF', linestyle = ':', label = 'Deciles')
             plt.plot(dates,percentiles['calc_value'],
                      'r', label = 'Measure')
             plt.ylabel('measure')
             plt.legend(bbox_to_anchor=(1.02, 1),
                        loc=2, borderaxespad=0.,fontsize=10)
```

```
        #plot percentile and determined percentile
        plt.subplot(312)
        plt.plot(dates,percentiles['percentiles'],
                 'g', label = 'Percentile')
        plt.plot(dates,cusum_out['alert_percentile_pos'],
                 'ro', label = 'Increase alerts')
        plt.plot(dates,cusum_out['reference_percentile'],
                 'b', label = 'Reference percentile')
        plt.plot(dates,cusum_out['alert_percentile_neg'],
                 'go', label = 'Decrease alerts')
        plt.ylabel('percentile')
        plt.legend(bbox_to_anchor=(1.02, 1),
                   loc=2, borderaxespad=0.,fontsize=10)

        #plot CUSUM/threshold graph
        plt.subplot(313)
        plt.plot(dates,cusum_out['smax'],
                 'c', label = 'CUSUM +ve')
        plt.plot(dates,cusum_out['smin'],
                 'r', label = 'CUSUM -ve')
        plt.plot(dates,cusum_out['threshold'],
                 'k--', label = 'Thresholds')
        plt.plot(dates,negthreshold,
                 'k--')
        plt.ylabel('CUSUM')
        plt.legend(bbox_to_anchor=(1.02, 1),
                   loc=2, borderaxespad=0.,fontsize=10)

        plt.show()
        print ''
        print ''
        print ''
```

1.5 Testing

```
In [18]: #Analyse
         def analyse(measure, location):
             if len(location) == 3:
                 print "Measure: https://openprescribing.net/ccg/%s/#%s" % (locatio
             else:
                 print "Measure: https://openprescribing.net/practice/%s/#%s" % (lo
             #CHANGE THESE to alter the MEASURE and/or CCG/PRACTICE
             percentiles_dates = get_percentiles_for(measure, location)
             dates = [datetime.datetime.strptime(date, '%Y-%m-%d').date()
                      for date in percentiles_dates['dates']]
             deciles = get_deciles(measure, location)
```

```
#CHANGE THESE NUMBERS to adjust SMOOTHING PERIOD and SENSITIVITY
cusum_out = cusum(percentiles_dates['percentiles'],12,5)
draw_plots(cusum_out, percentiles_dates, dates, deciles)
```

2 Appendix B -Example graphs

```
In [19]: #RUN ANALYIS AND DRAW PLOTS -for all measures for 05Y and G85138
         for measure in get_measures():
             for location in ['05Y','G85138']:
                 analyse(measure, location)
```

```
Measure: https://openprescribing.net/ccg/05Y/#fungal
```

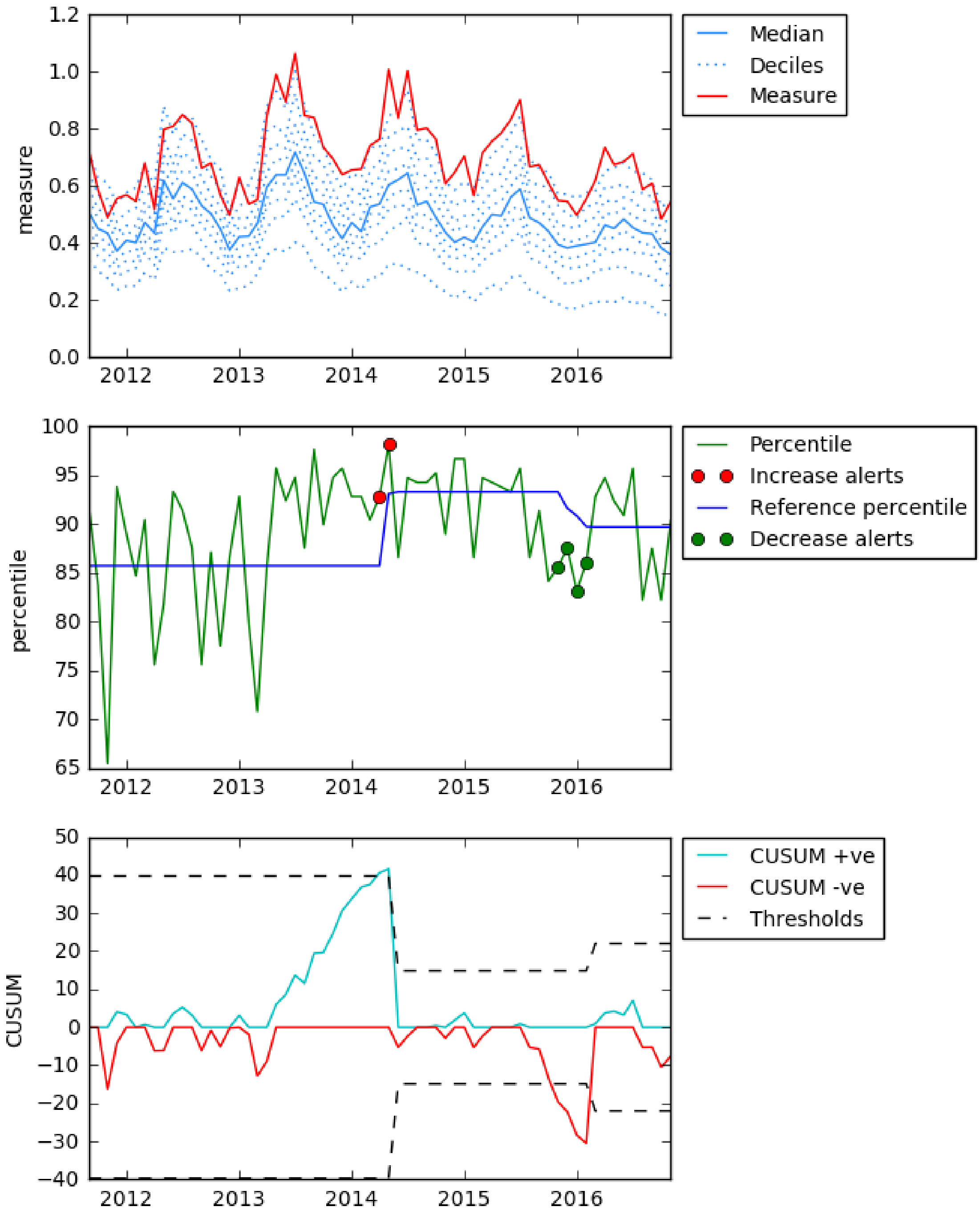

Measure: https://openprescribing.net/practice/G85138/#fungal

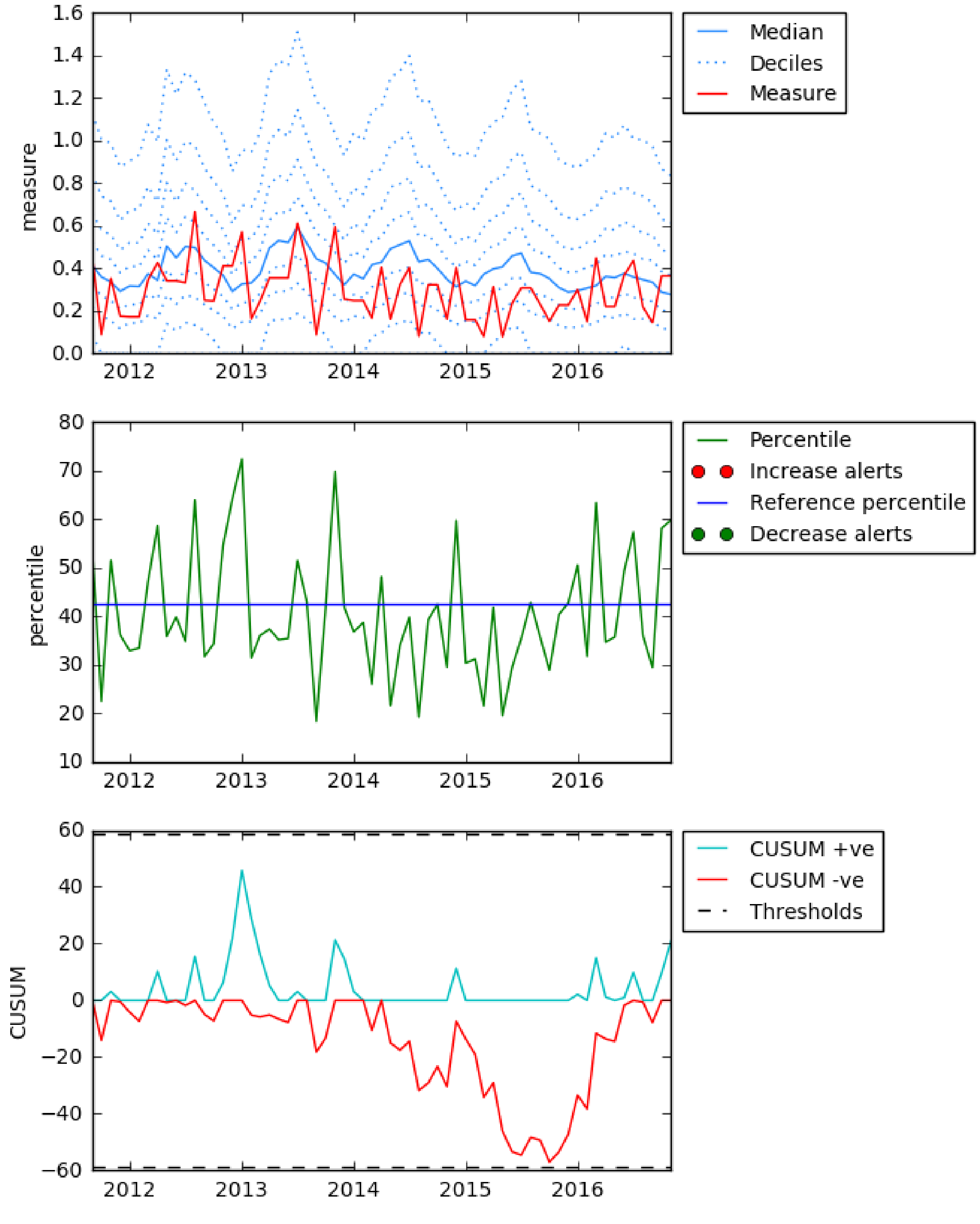

Measure: https://openprescribing.net/ccg/05Y/#ppi

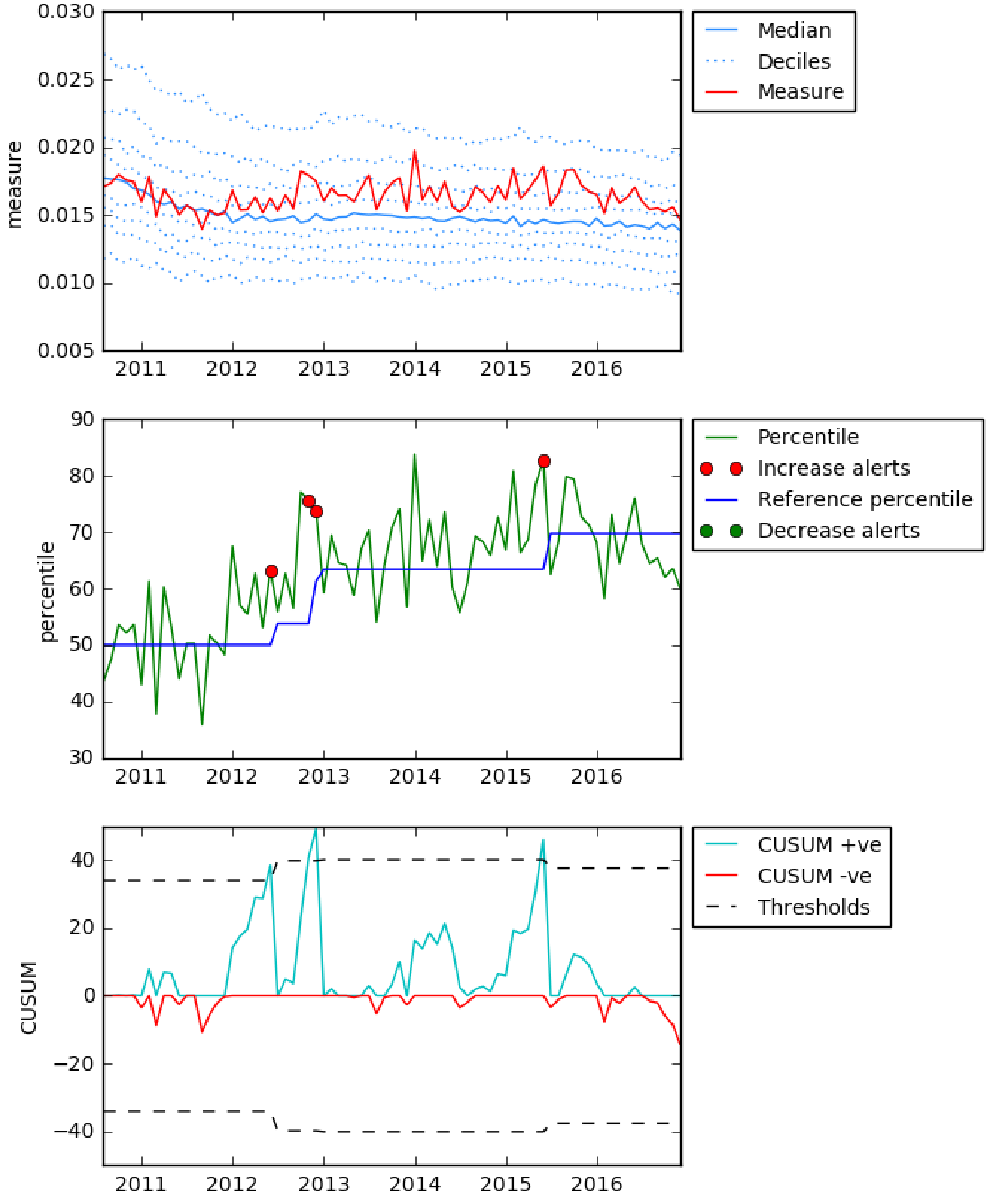

Measure: https://openprescribing.net/practice/G85138/#ppi

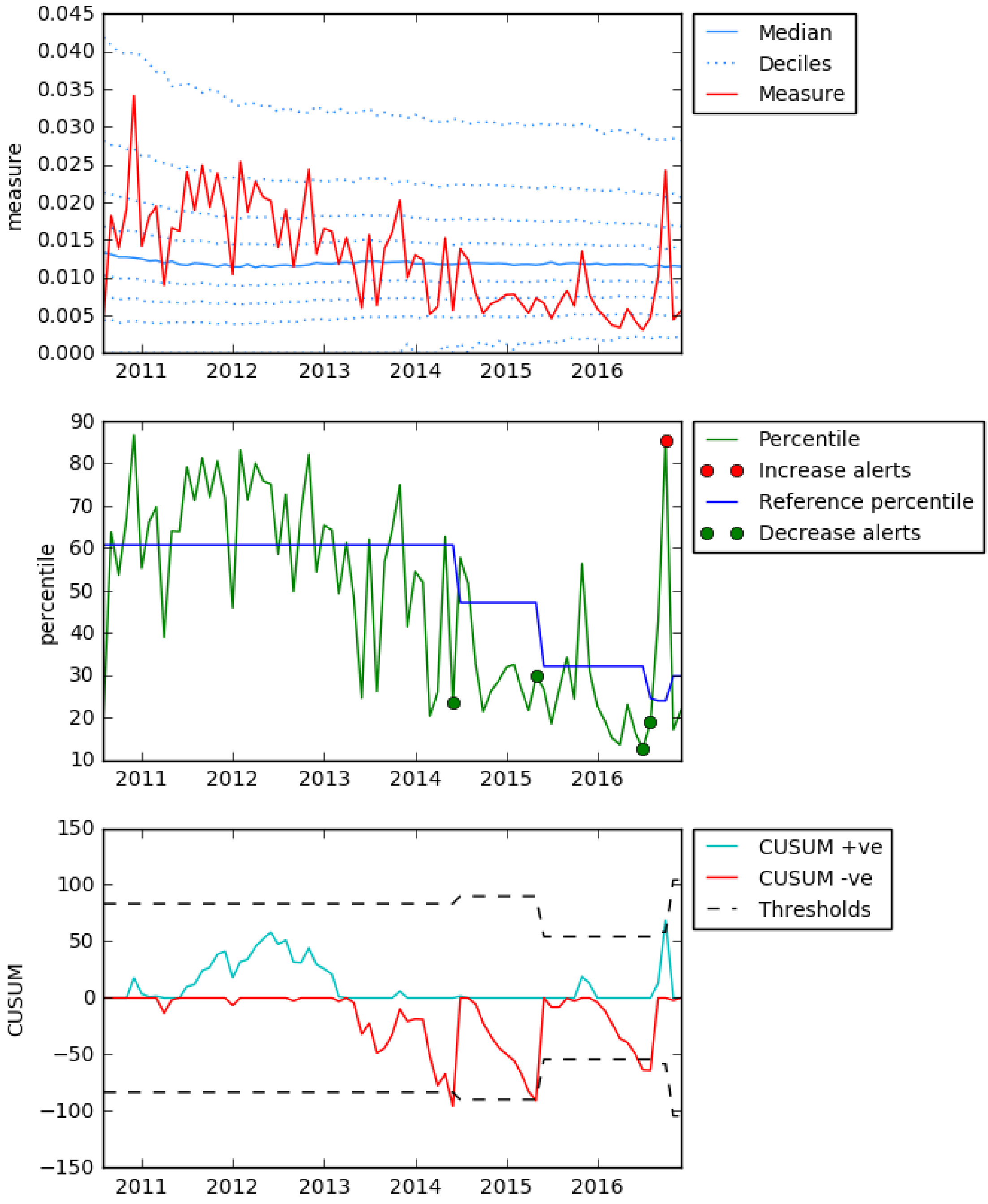

Measure: https://openprescribing.net/ccg/05Y/#tramadol

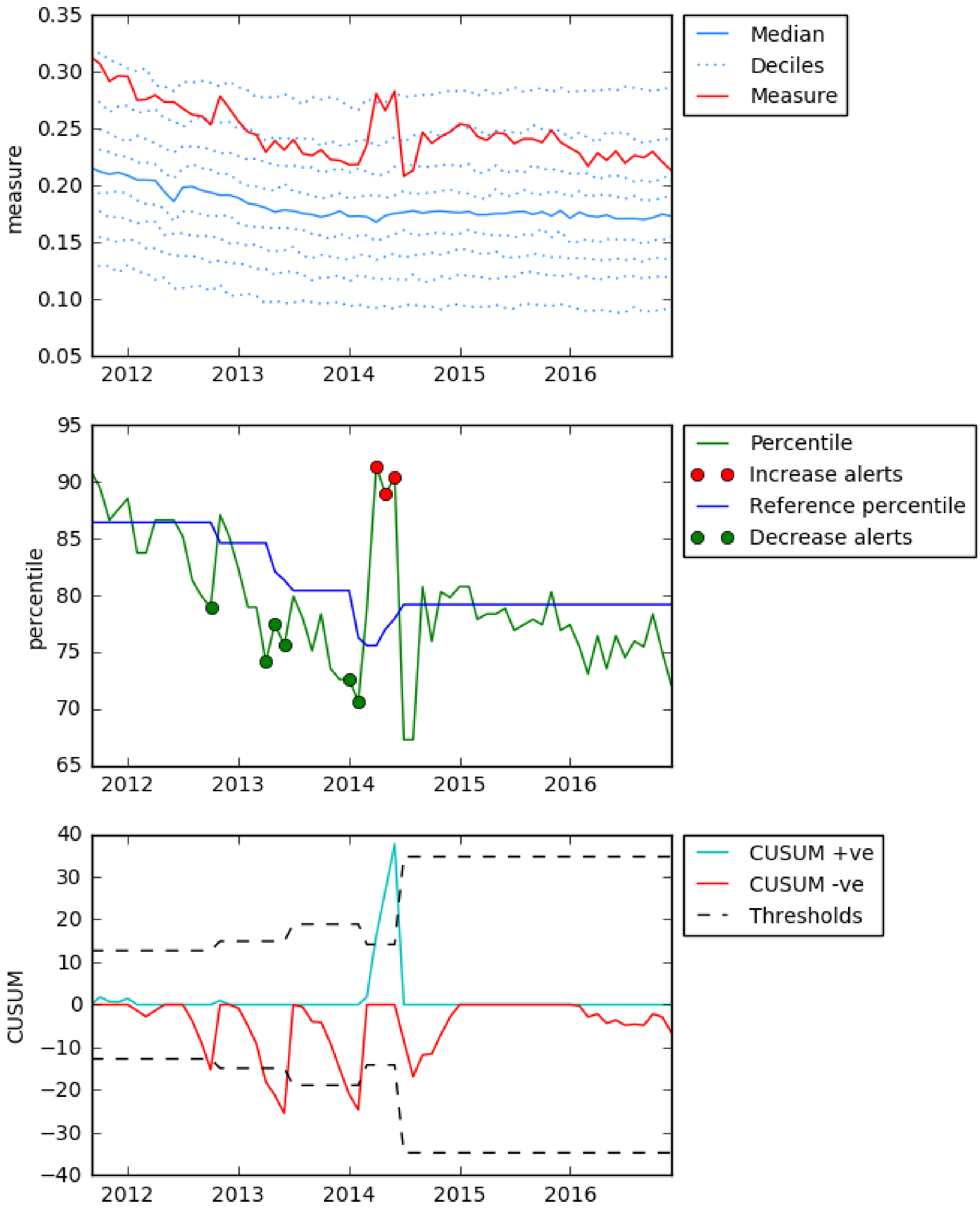

Measure: https://openprescribing.net/practice/G85138/#tramadol

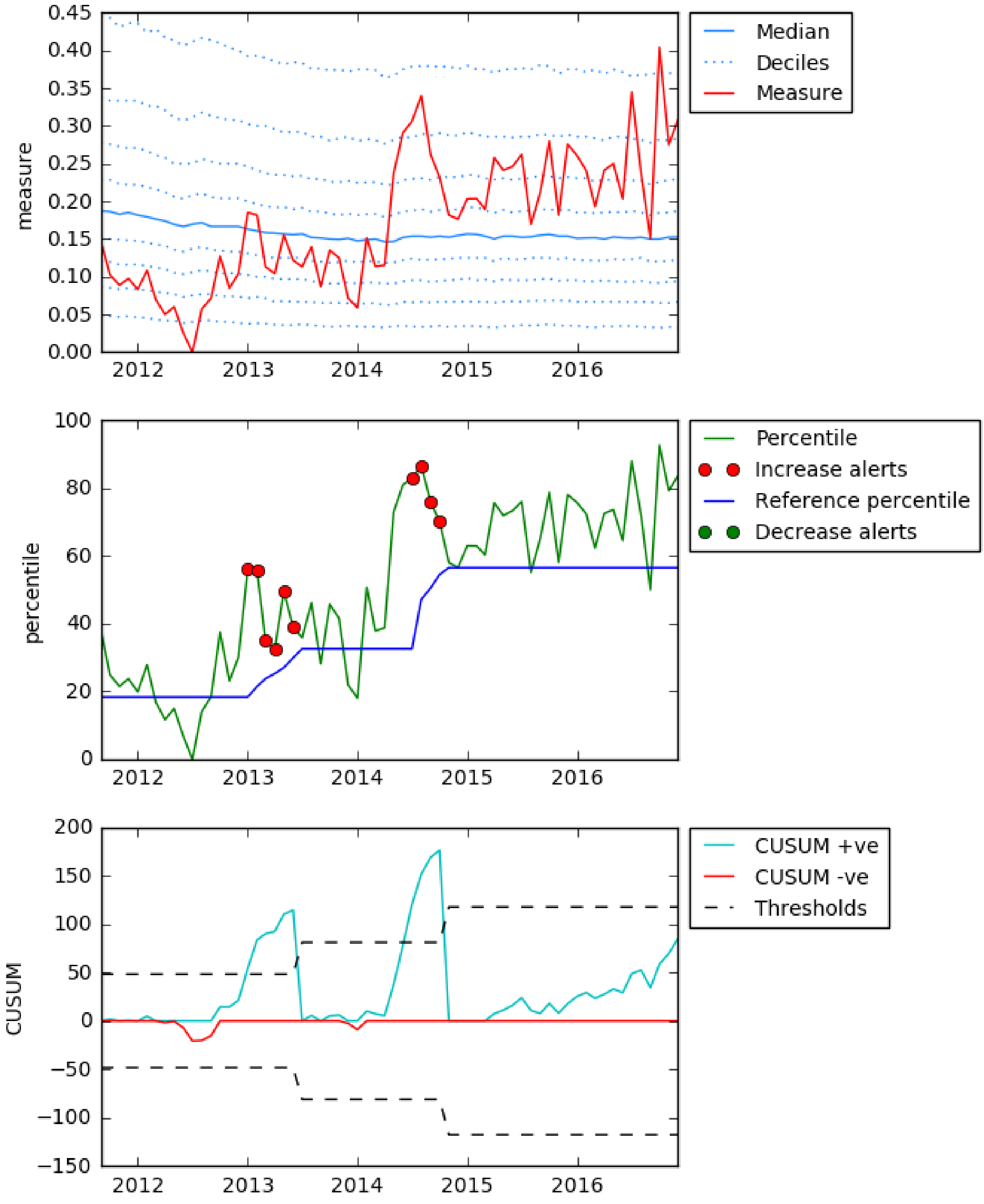

Measure: https://openprescribing.net/ccg/05Y/#sildenafil

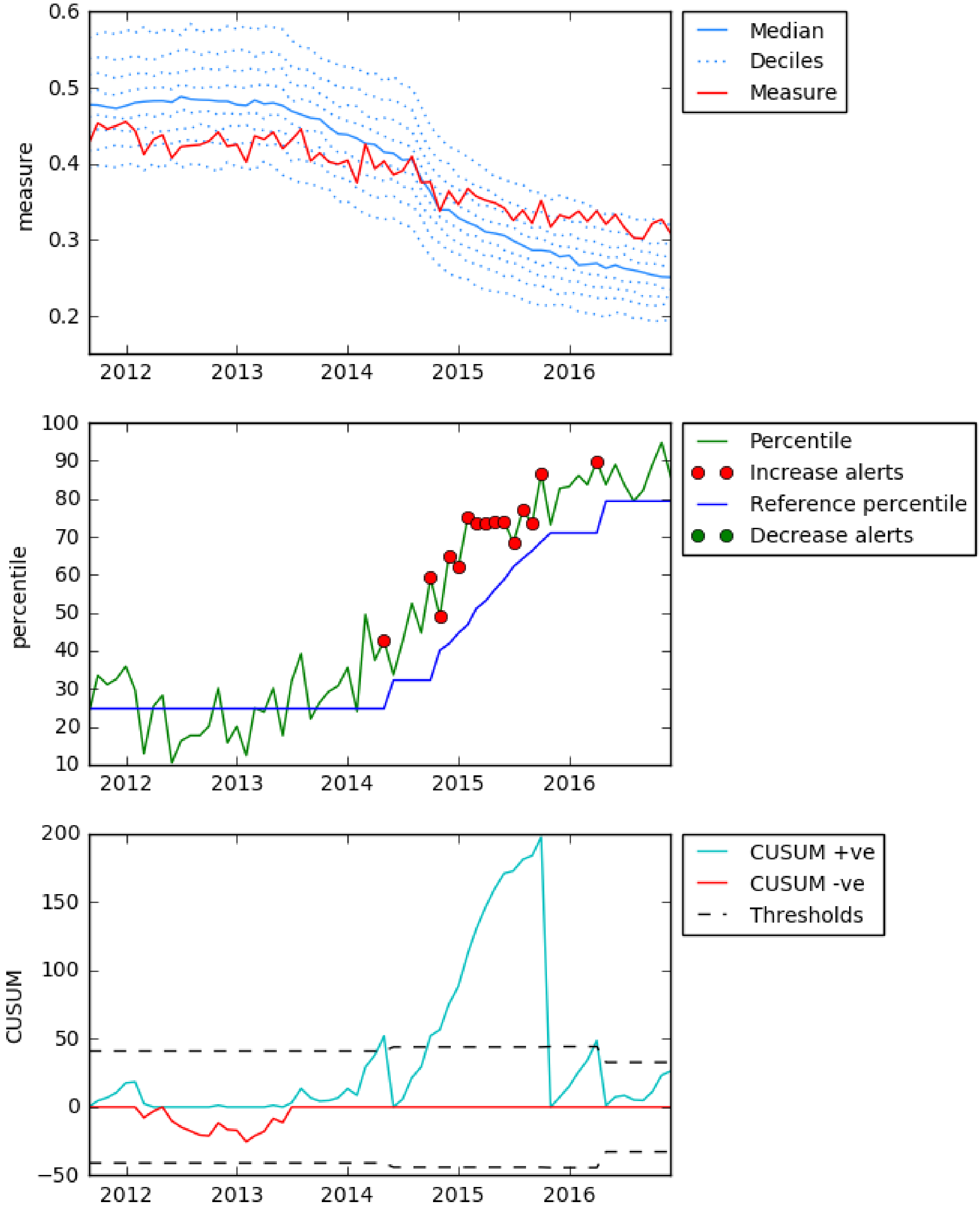

Measure: https://openprescribing.net/practice/G85138/#sildenafil

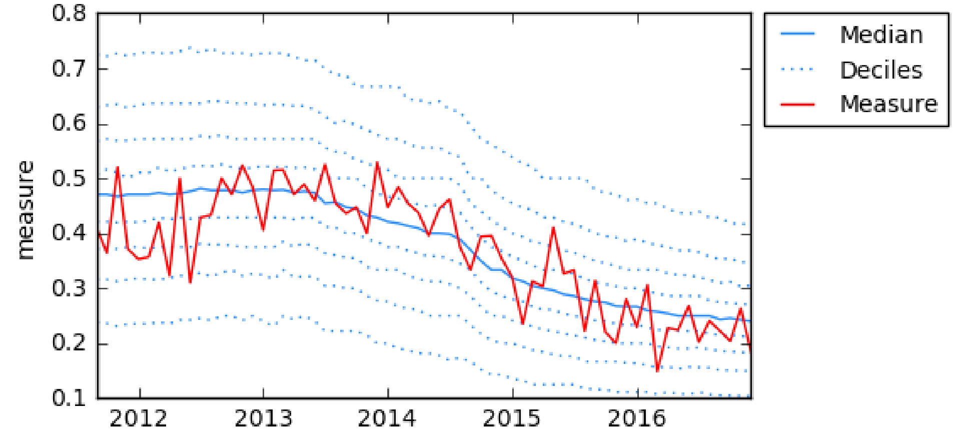

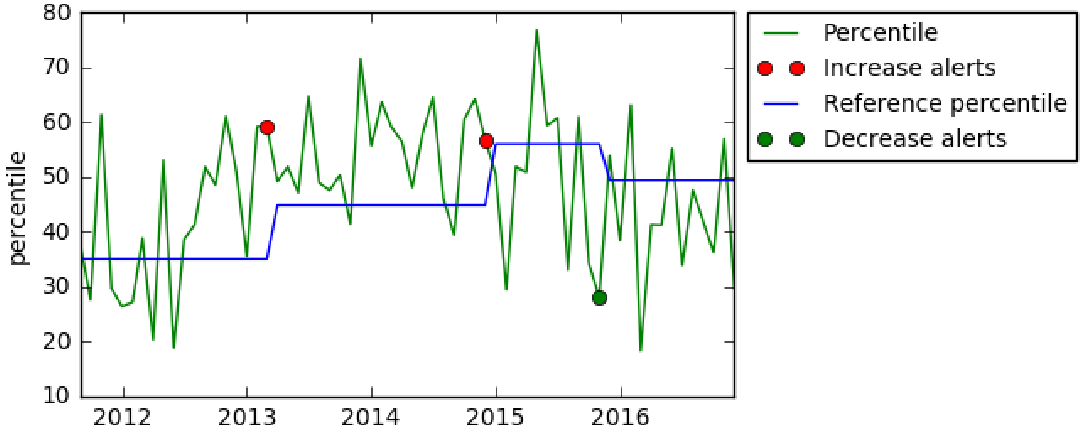

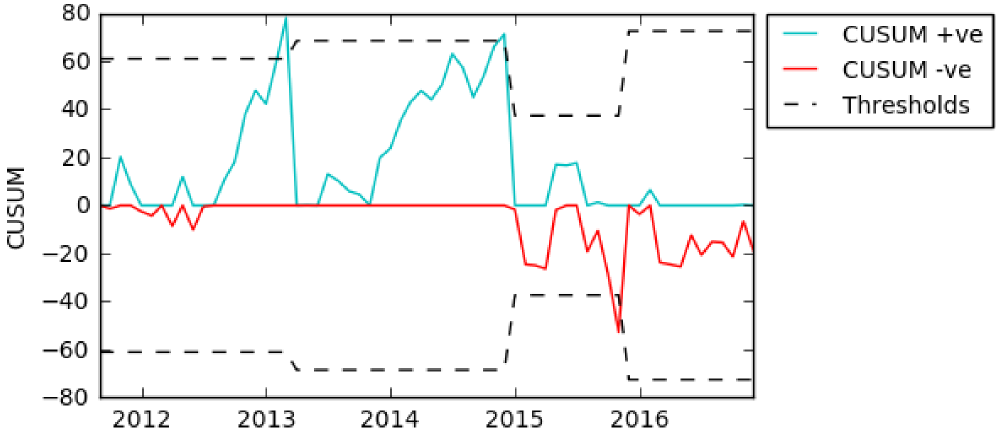

Measure: https://openprescribing.net/ccg/05Y/#arb

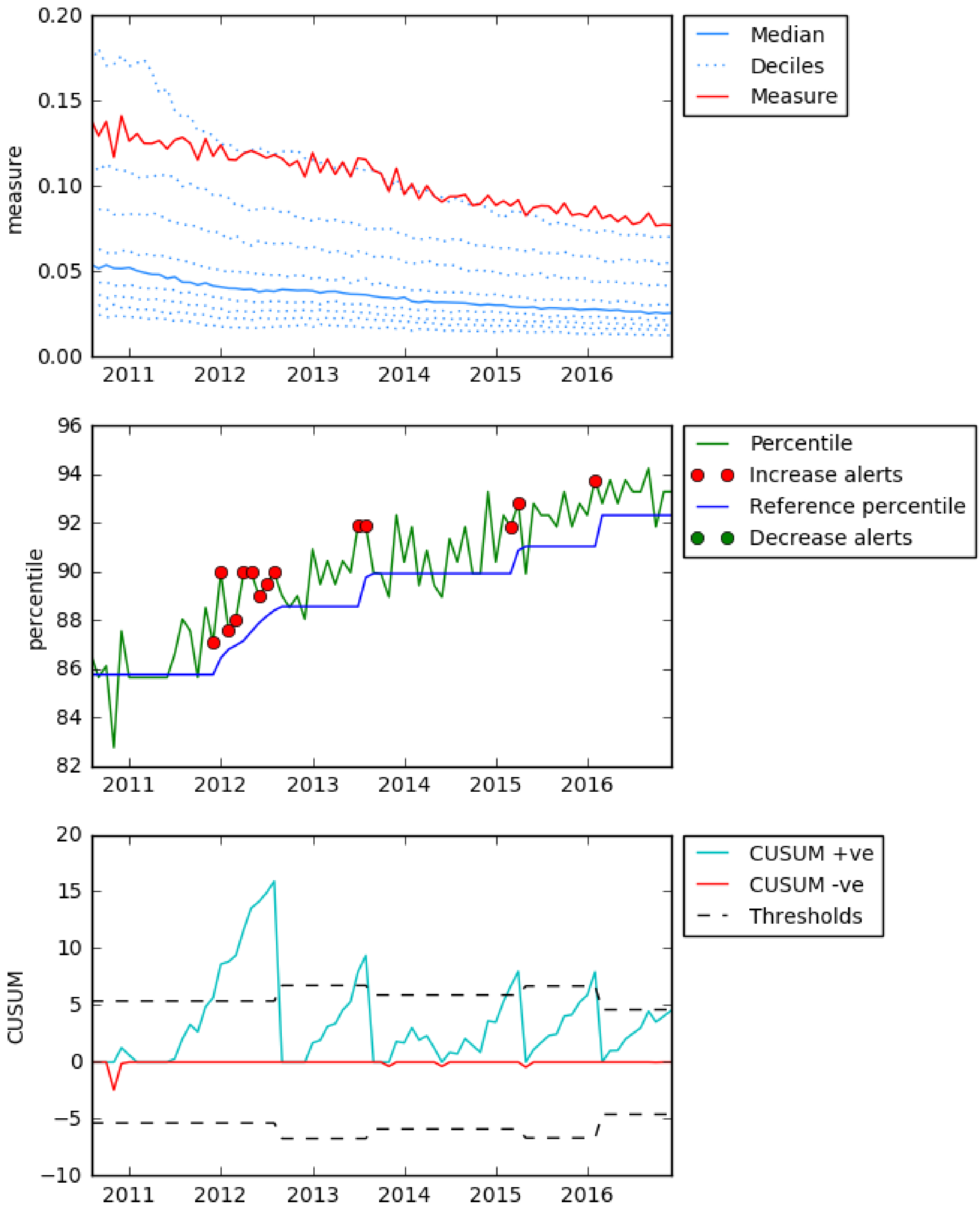

Measure: https://openprescribing.net/practice/G85138/#arb

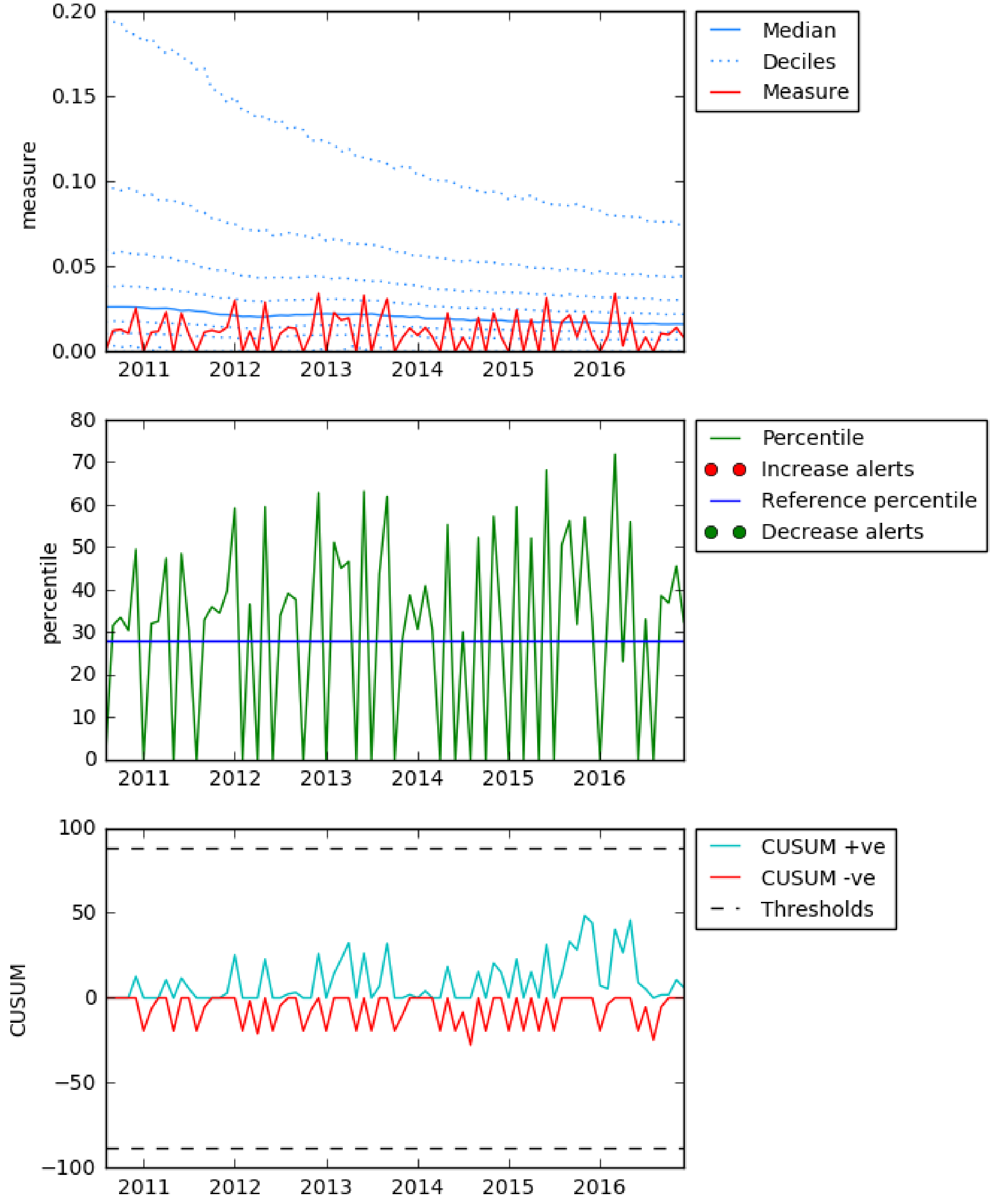

Measure: https://openprescribing.net/ccg/05Y/#icsdose

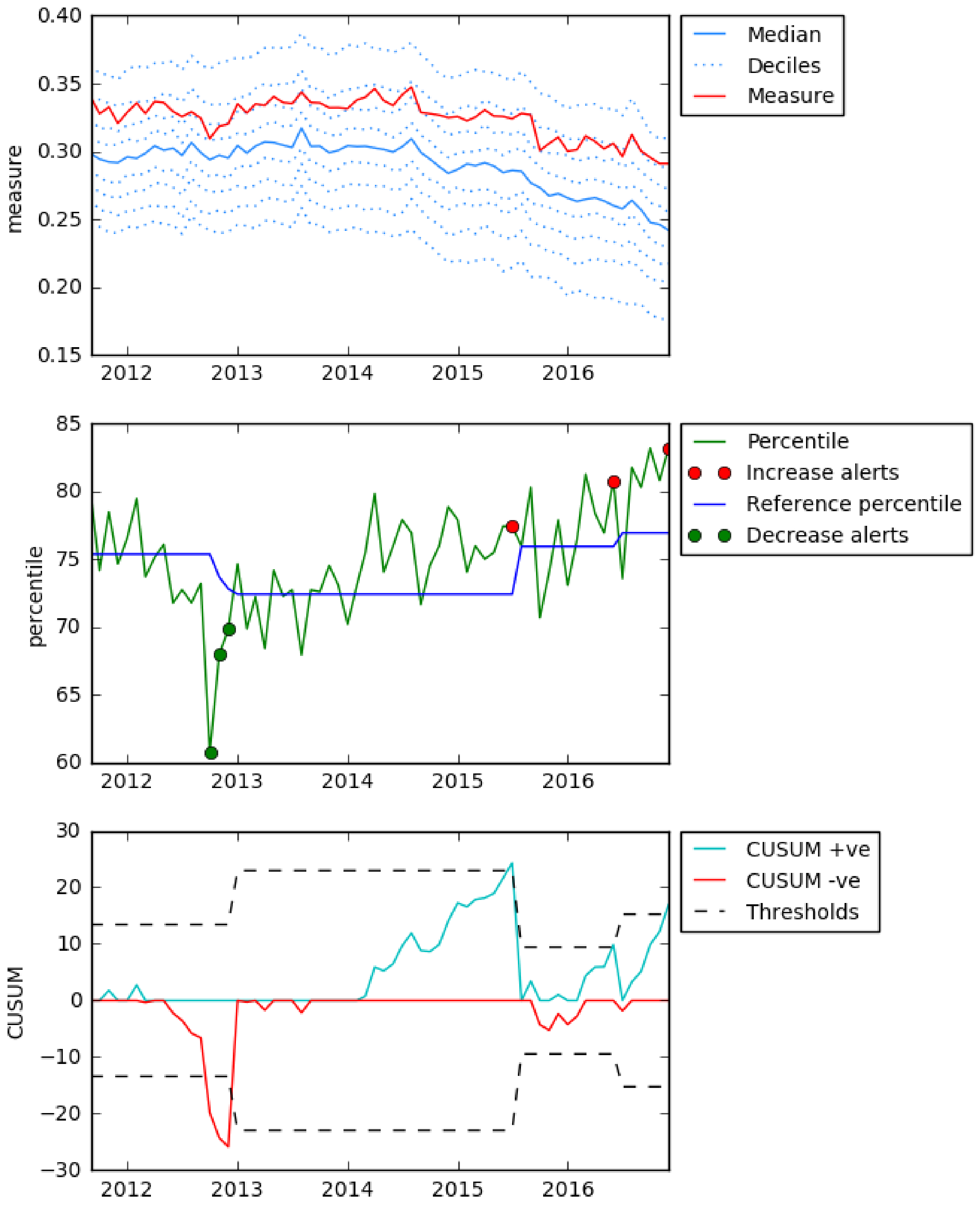

Measure: https://openprescribing.net/practice/G85138/#icsdose

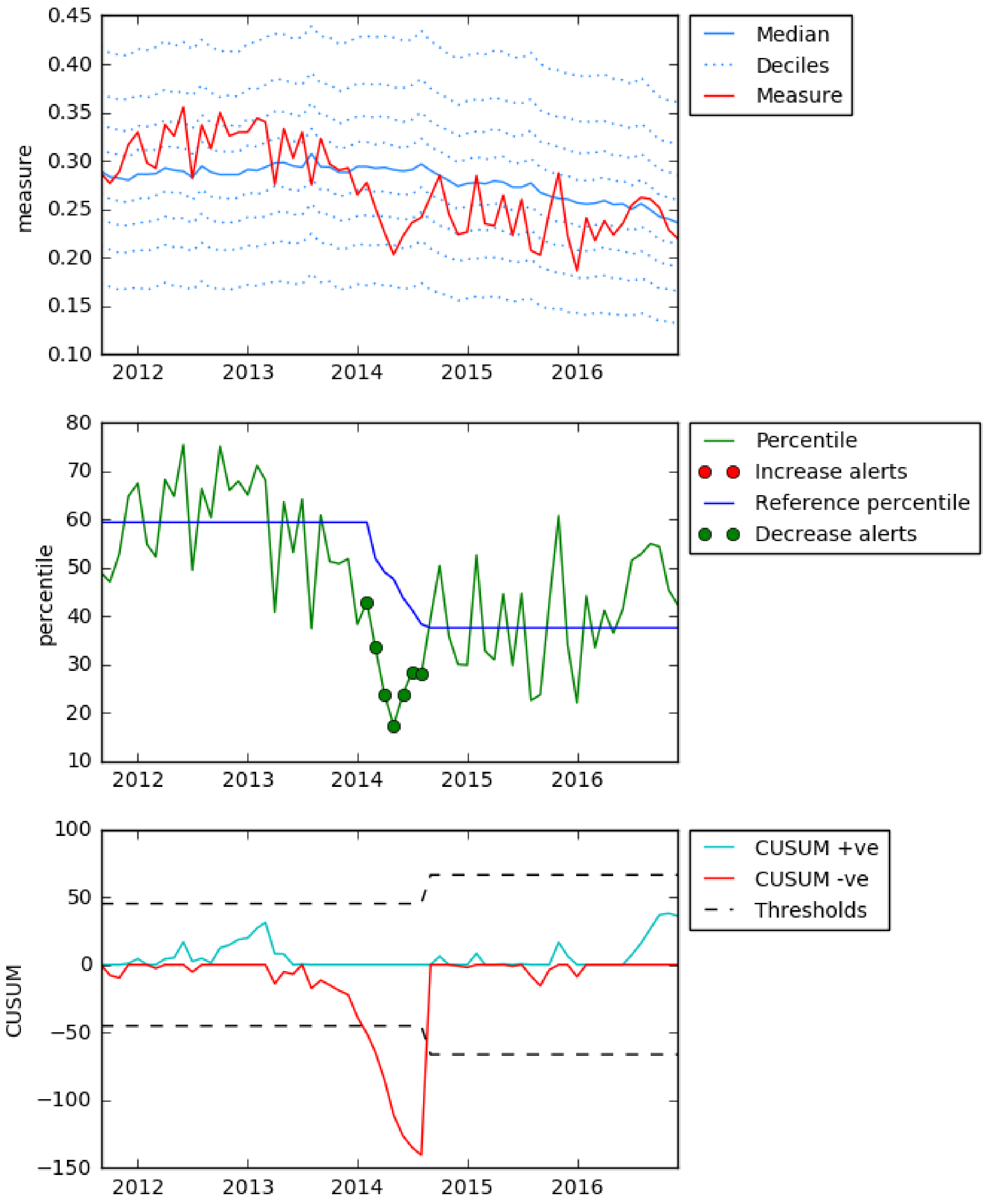

Measure: https://openprescribing.net/ccg/05Y/#dipyridamole

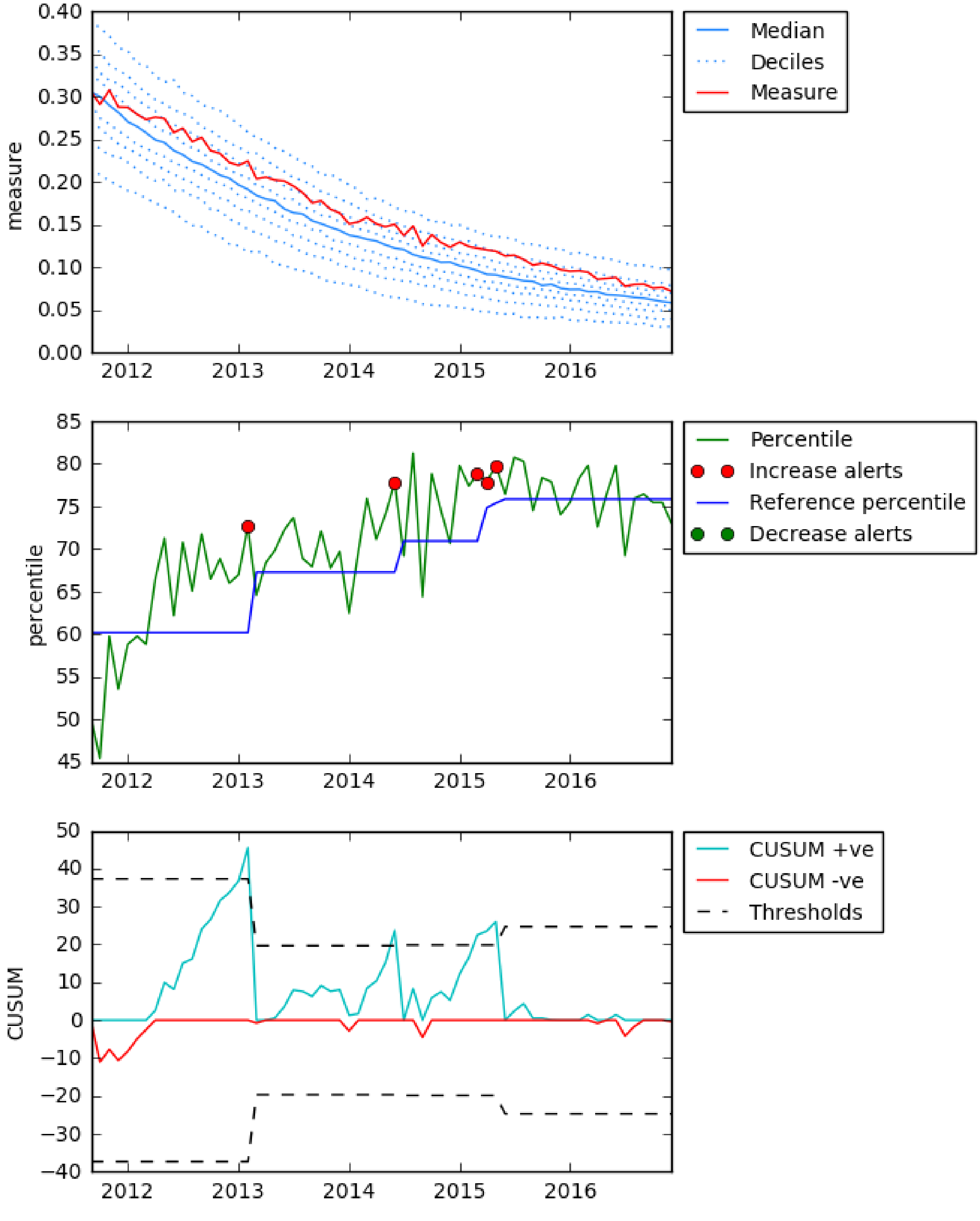

Measure: https://openprescribing.net/practice/G85138/#dipyridamole

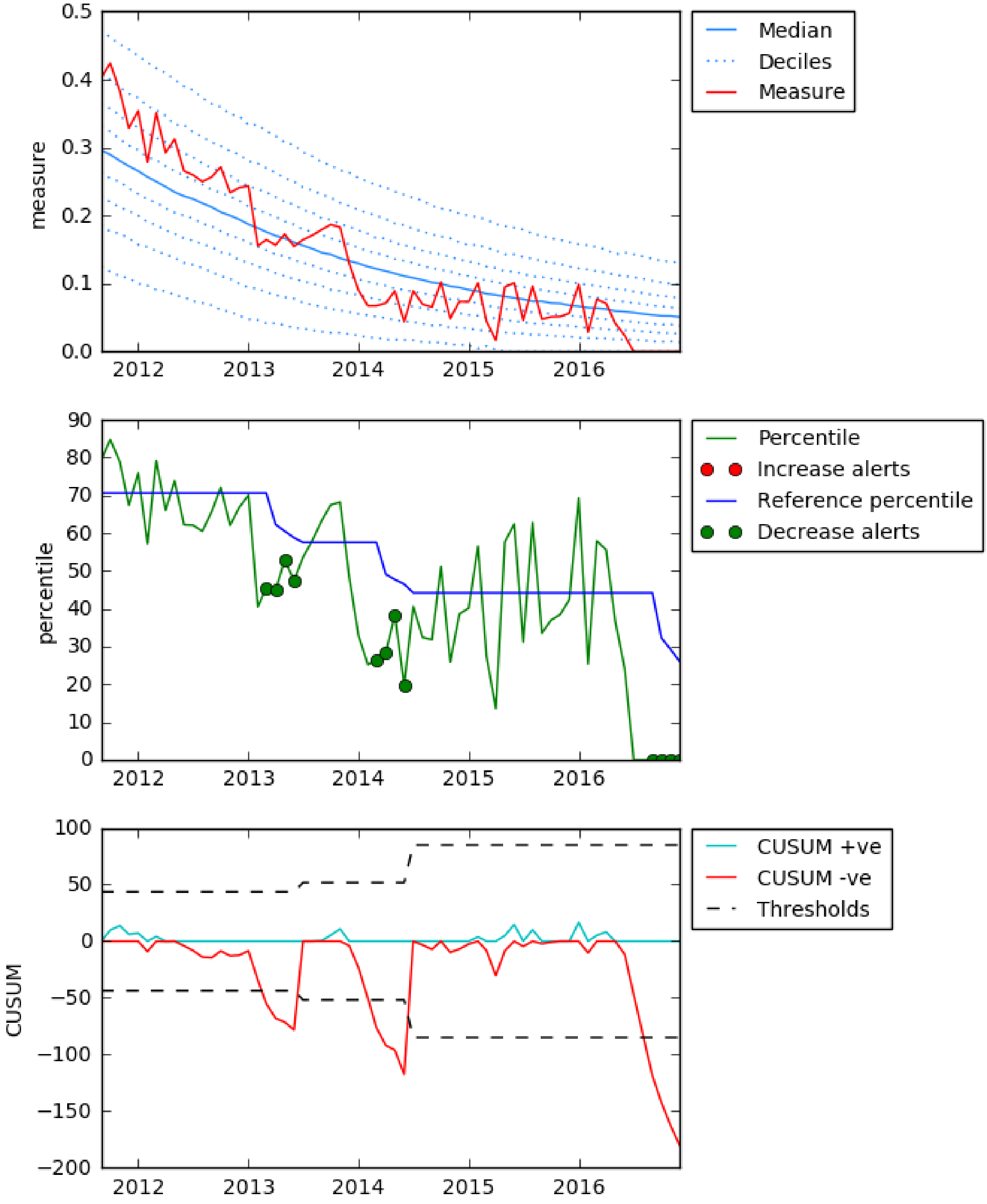

Measure: https://openprescribing.net/ccg/05Y/#methotrexate

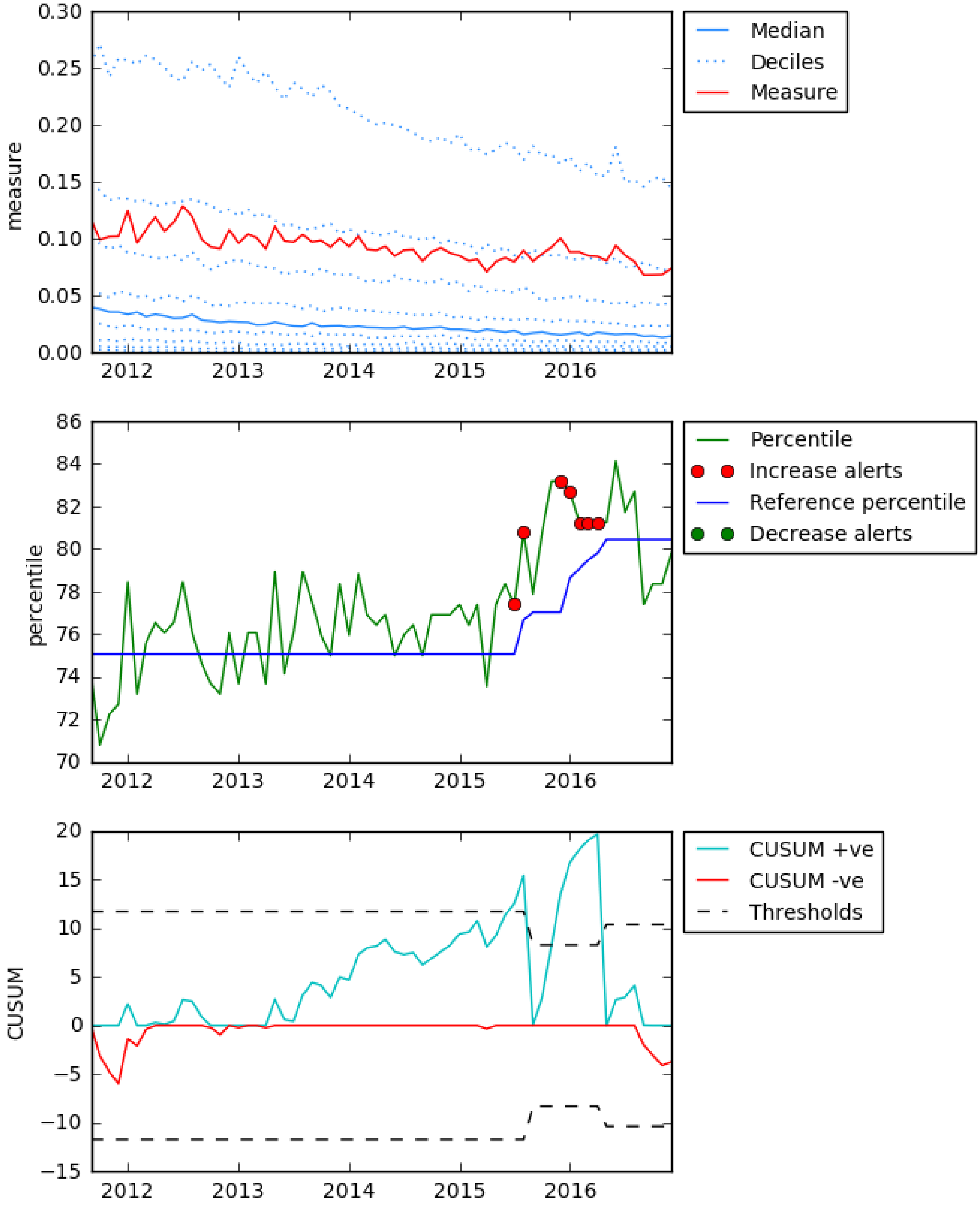

Measure: https://openprescribing.net/practice/G85138/#methotrexate

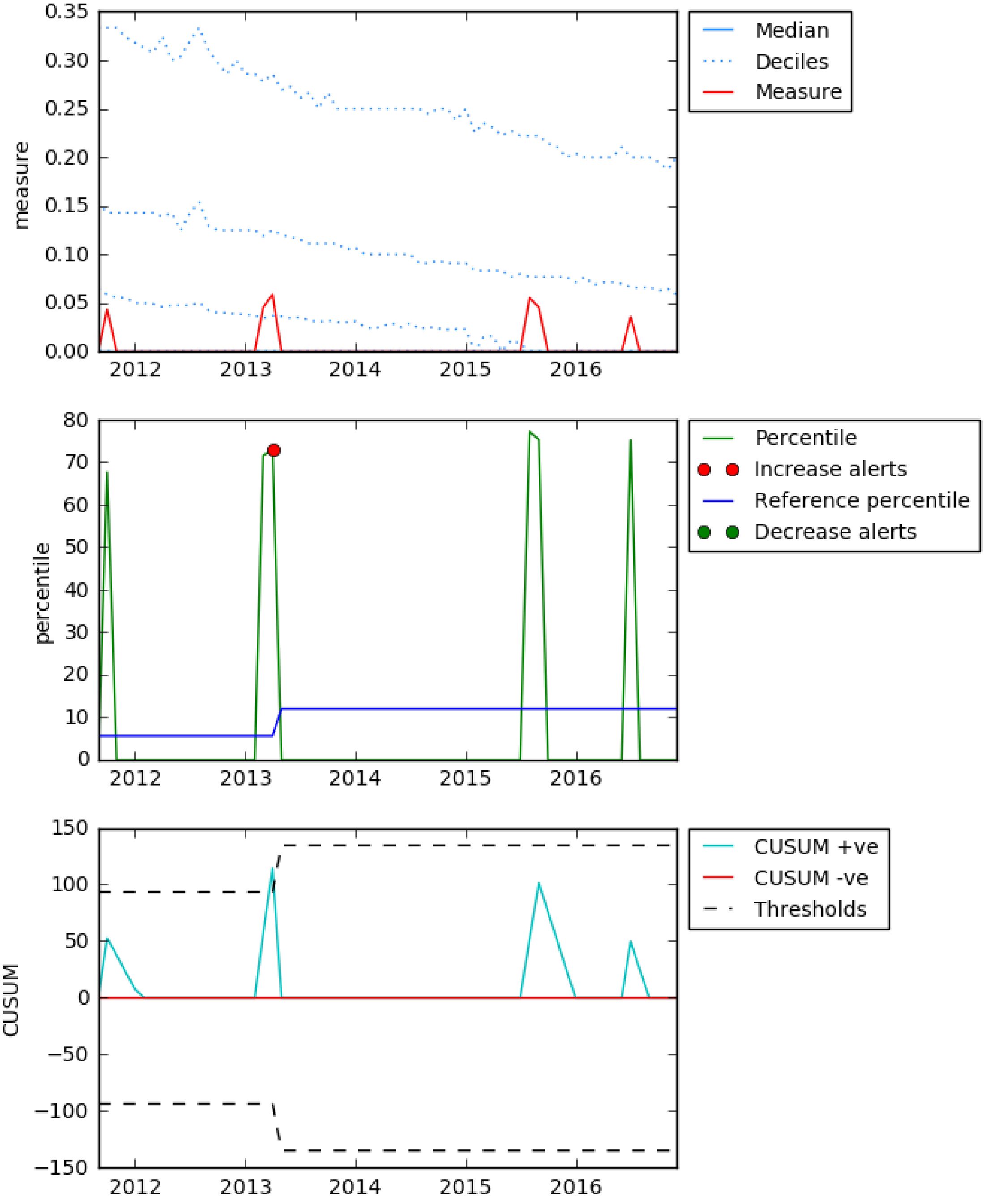

Measure: https://openprescribing.net/ccg/05Y/#vitb

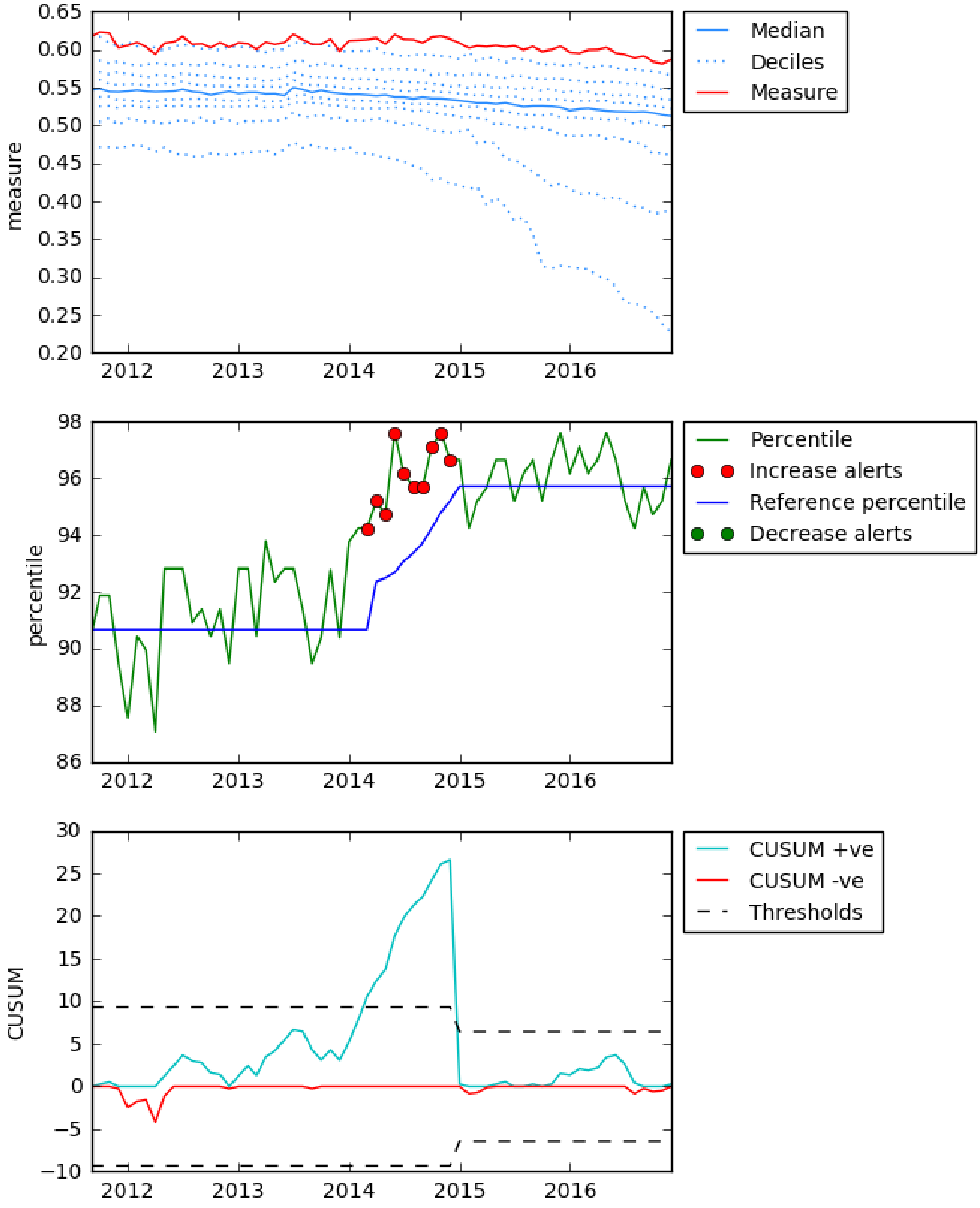

Measure: https://openprescribing.net/practice/G85138/#vitb

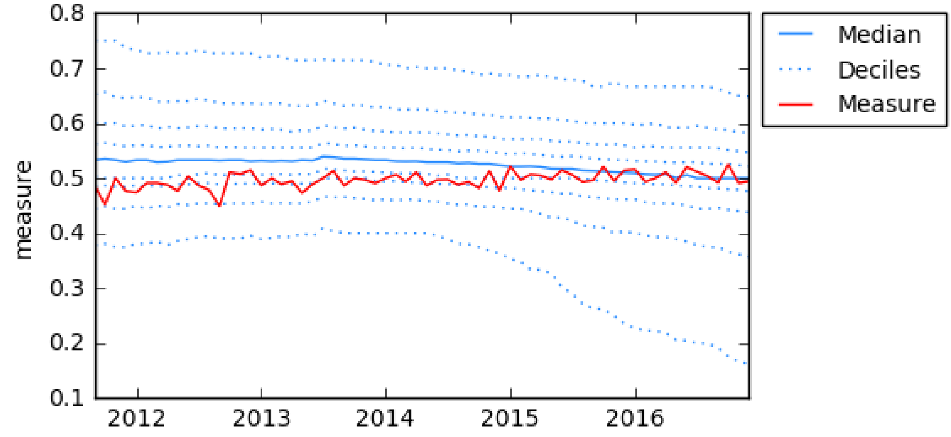

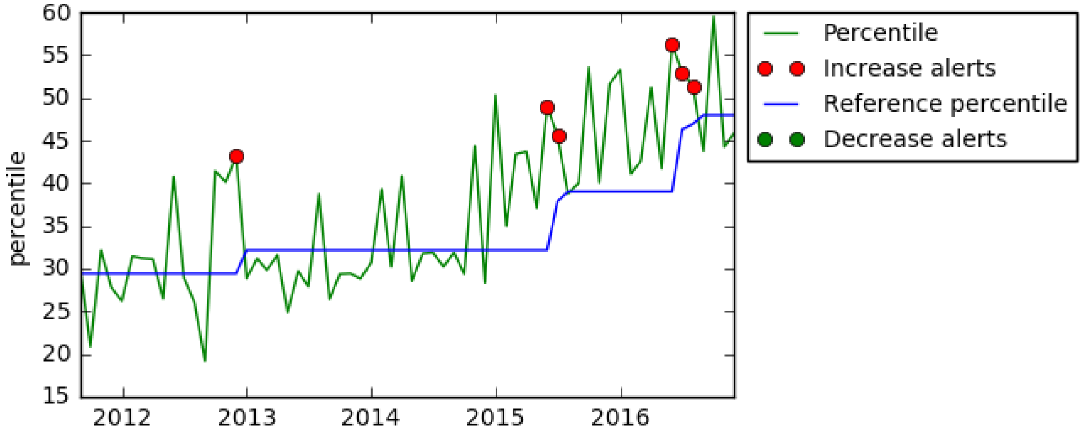

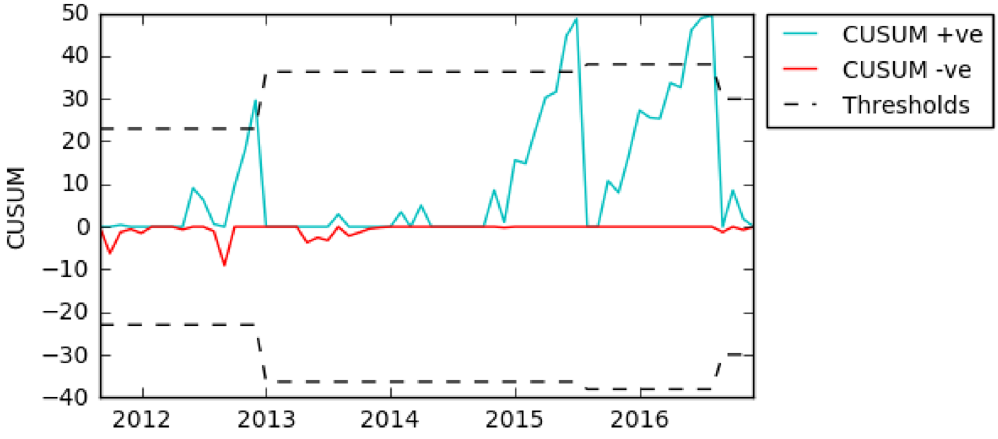

Measure: https://openprescribing.net/ccg/05Y/#solublepara

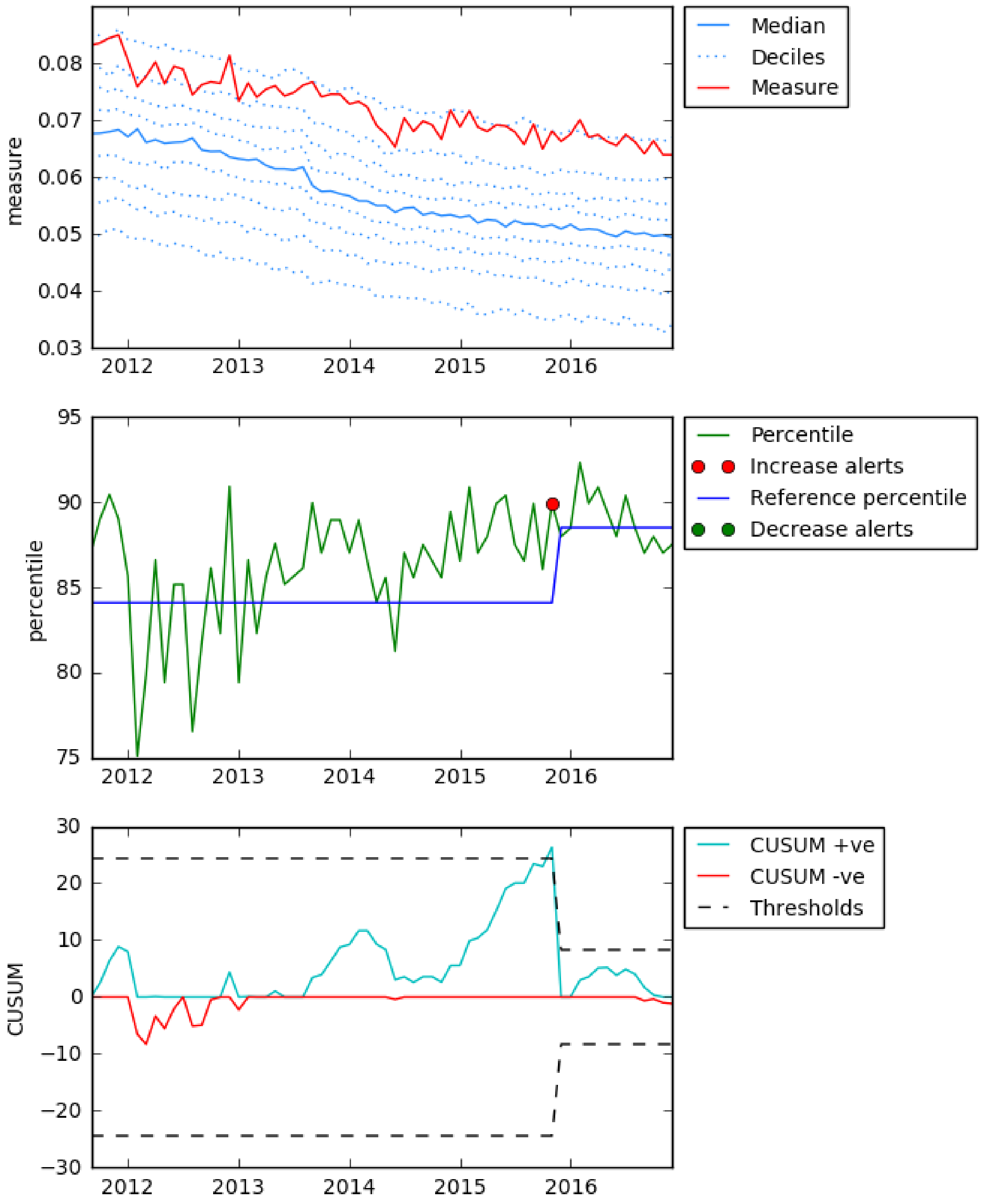

Measure: https://openprescribing.net/practice/G85138/#solublepara

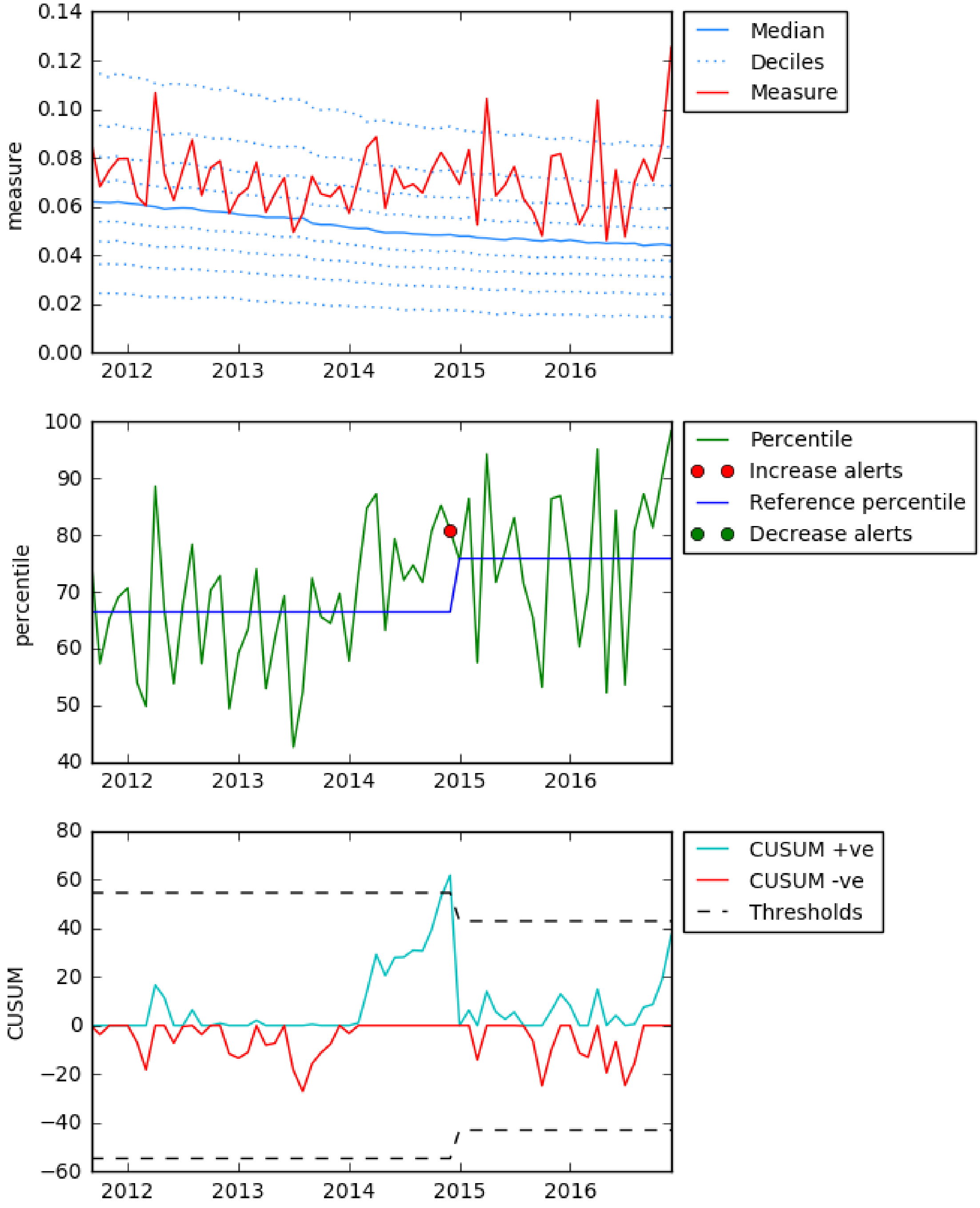

```
Measure: https://openprescribing.net/ccg/05Y/#ktt13_nsaids_ibuprofen
```

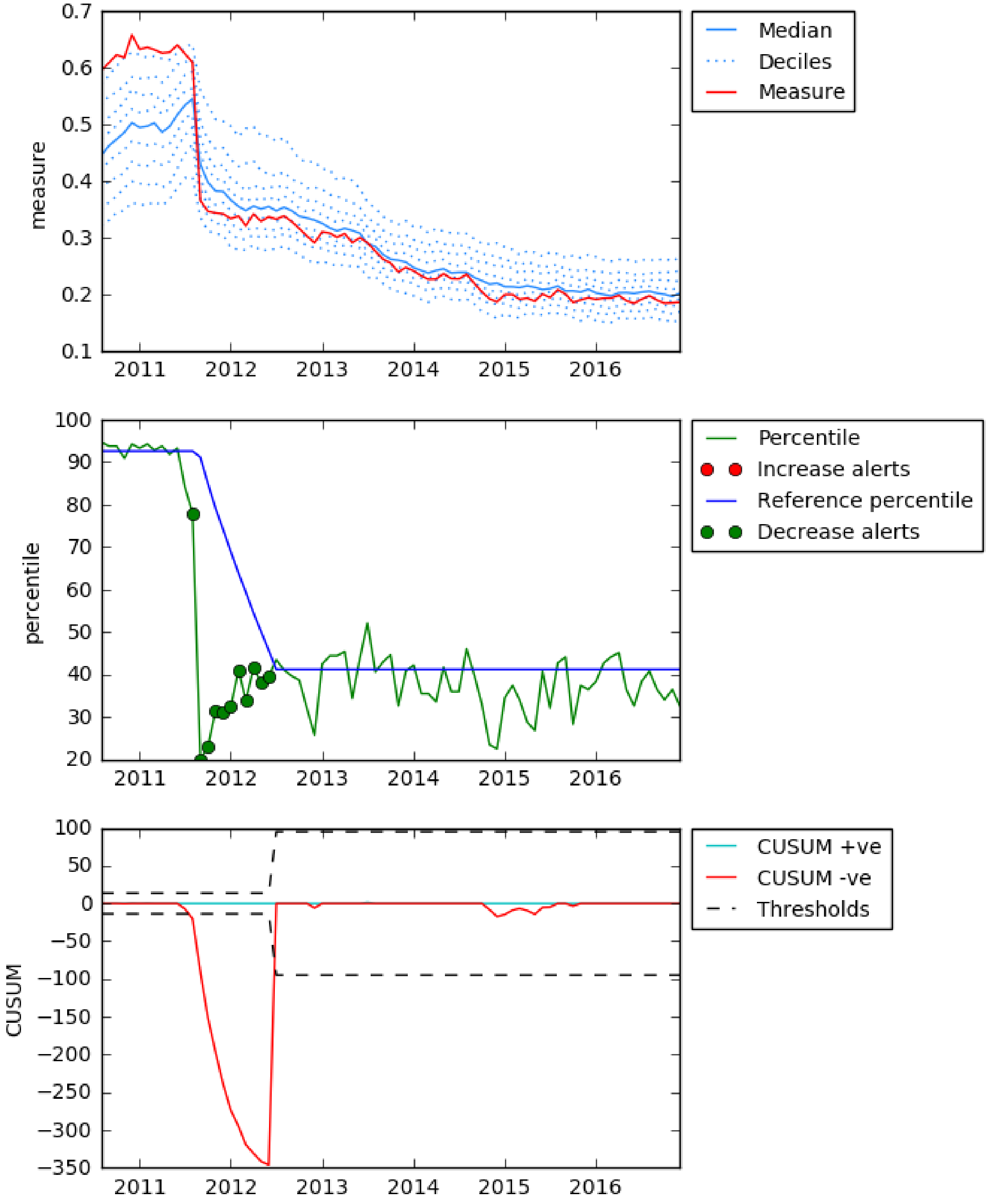

Measure: https://openprescribing.net/practice/G85138/#ktt13_nsaids_ibuprofen

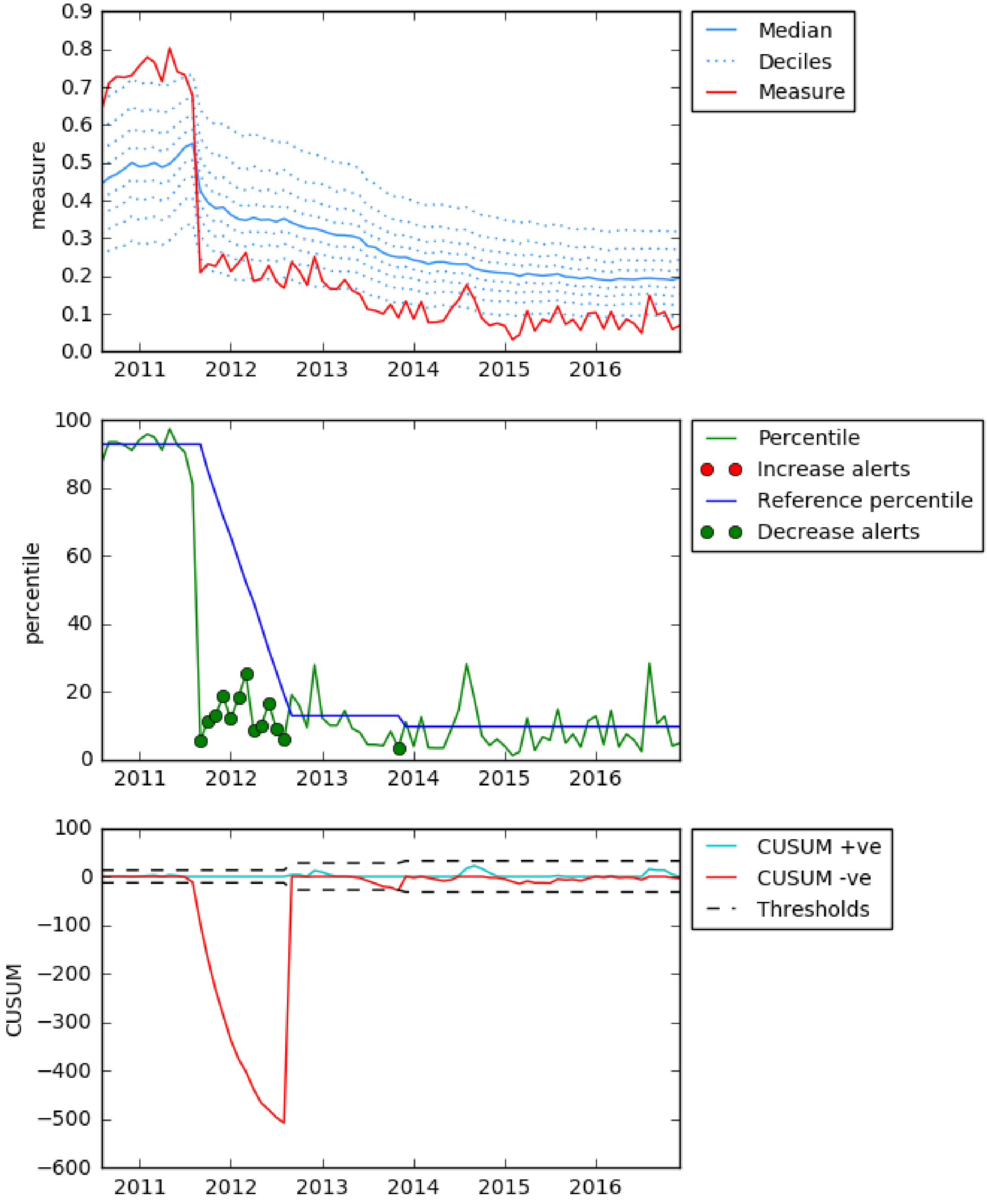

Measure: https://openprescribing.net/ccg/05Y/#ktt3_lipid_modifying_drugs

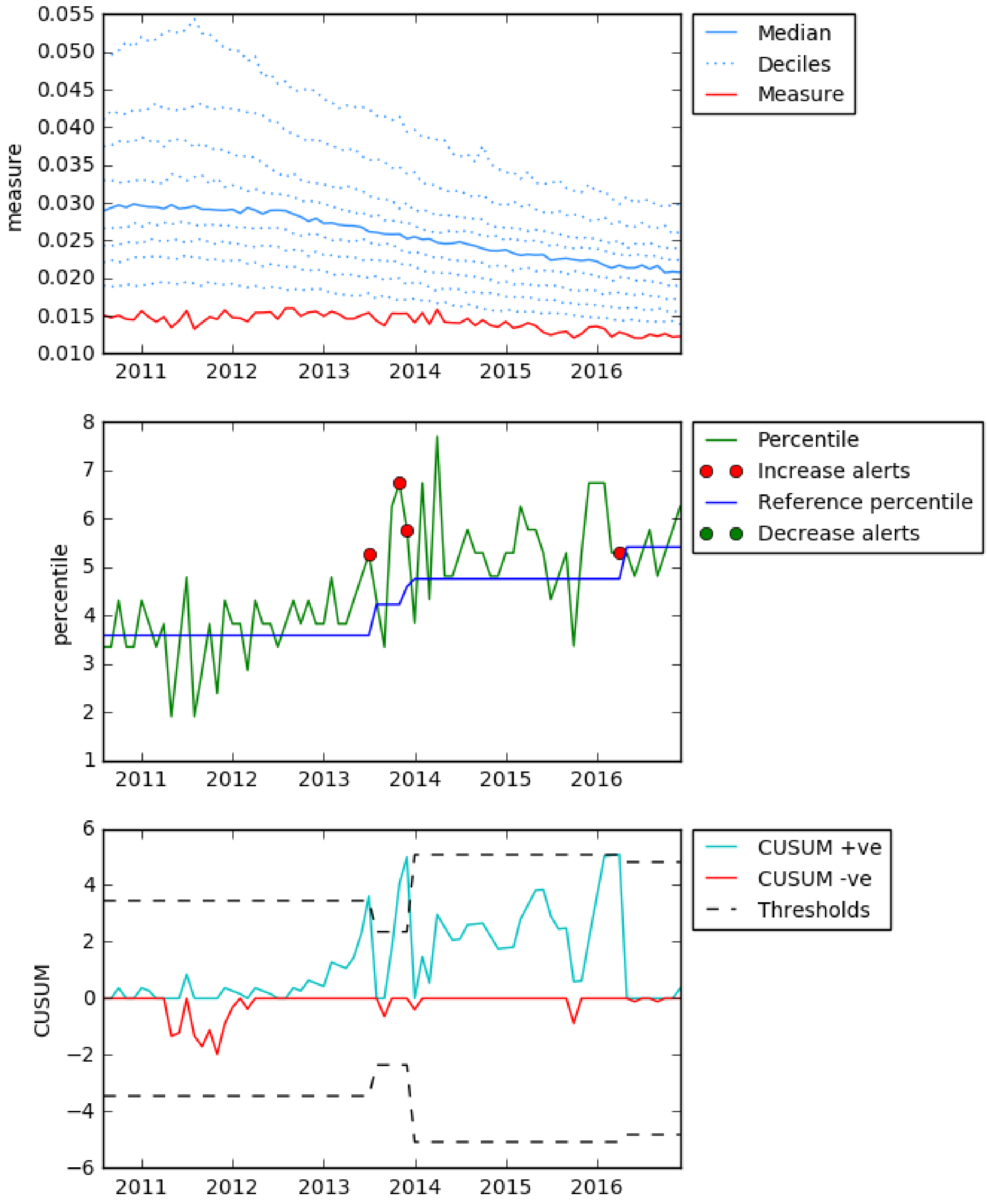

Measure: https://openprescribing.net/practice/G85138/#ktt3_lipid_modifying_drugs

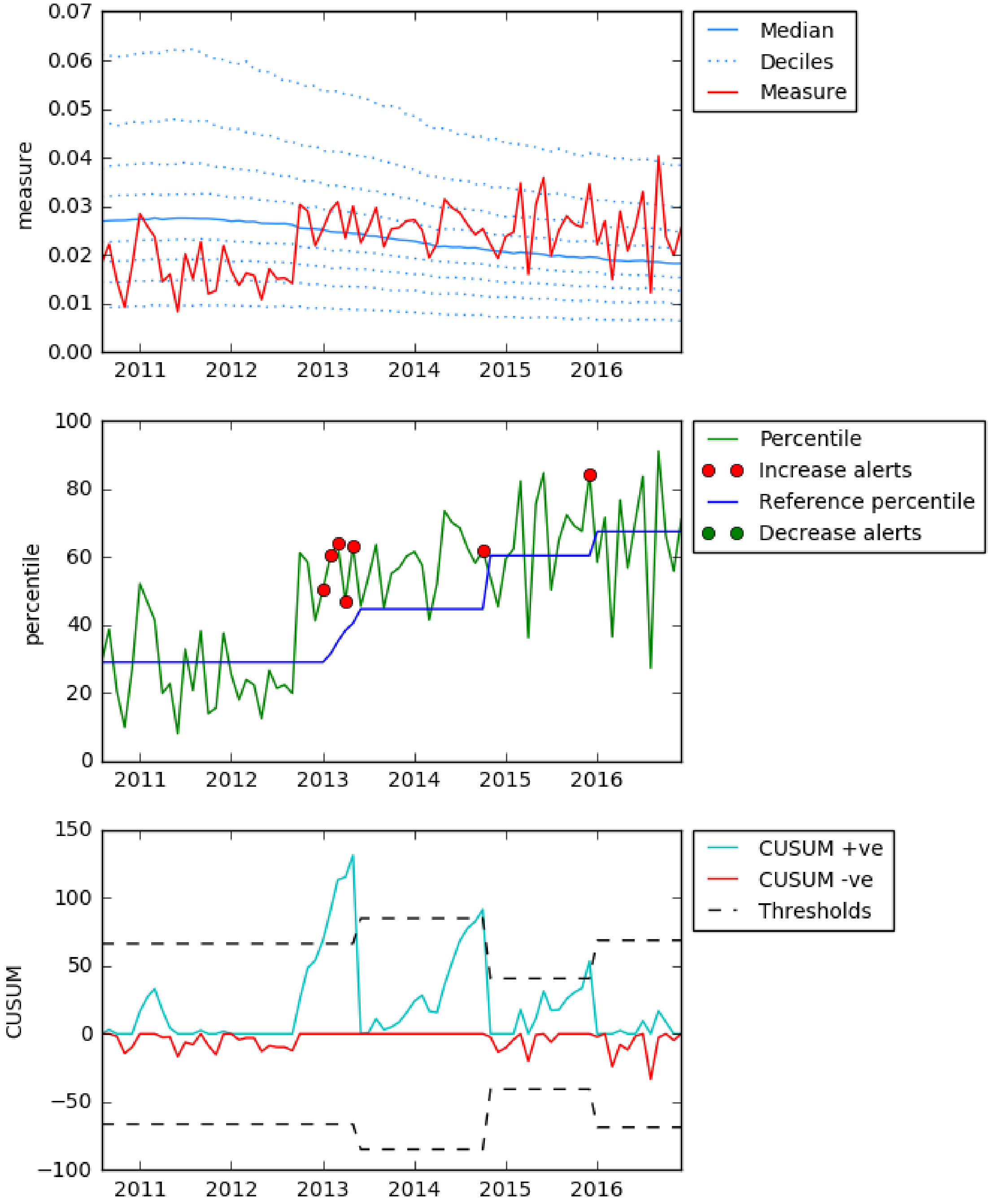

Measure: https://openprescribing.net/ccg/05Y/#doacs

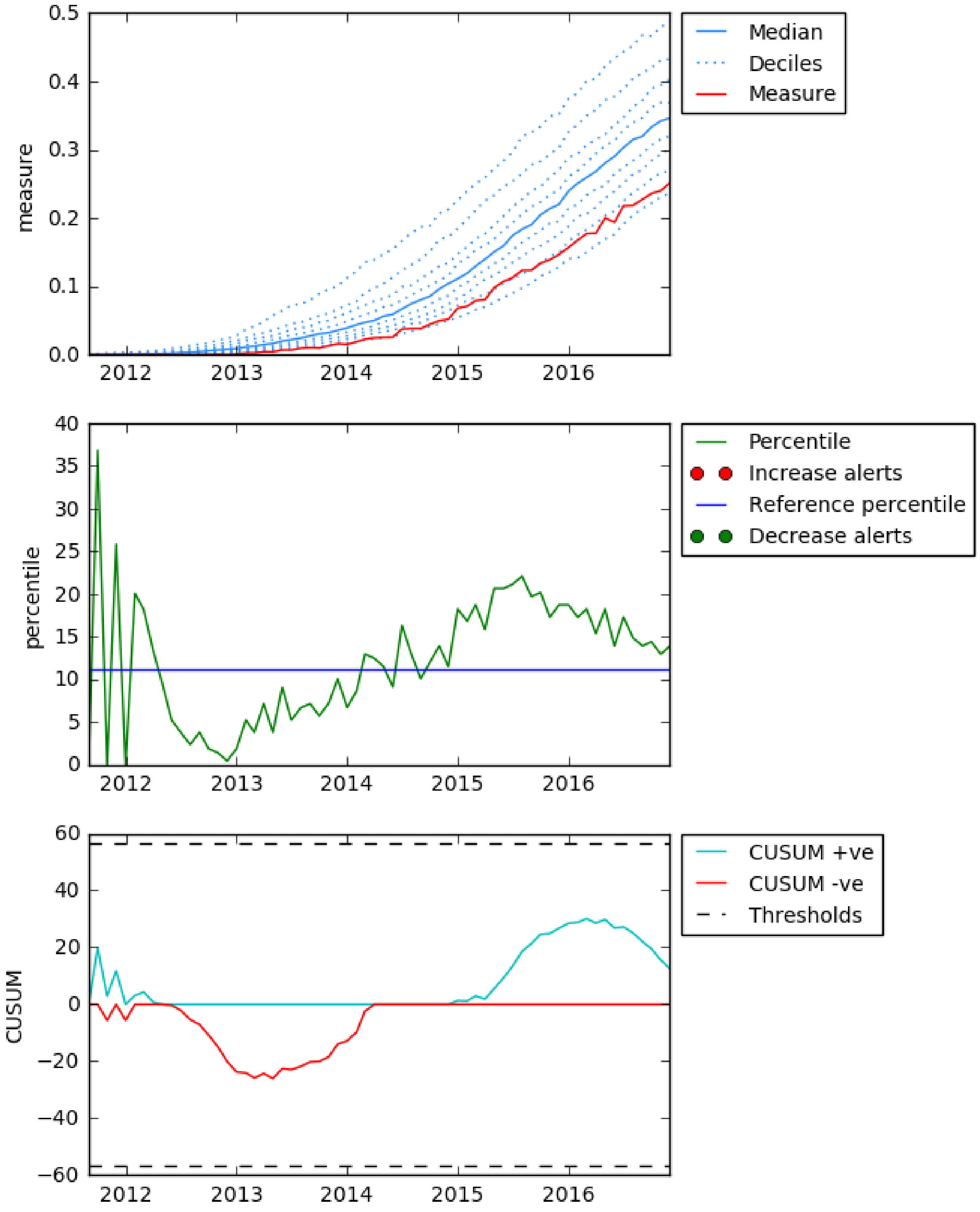

Measure: https://openprescribing.net/practice/G85138/#doacs

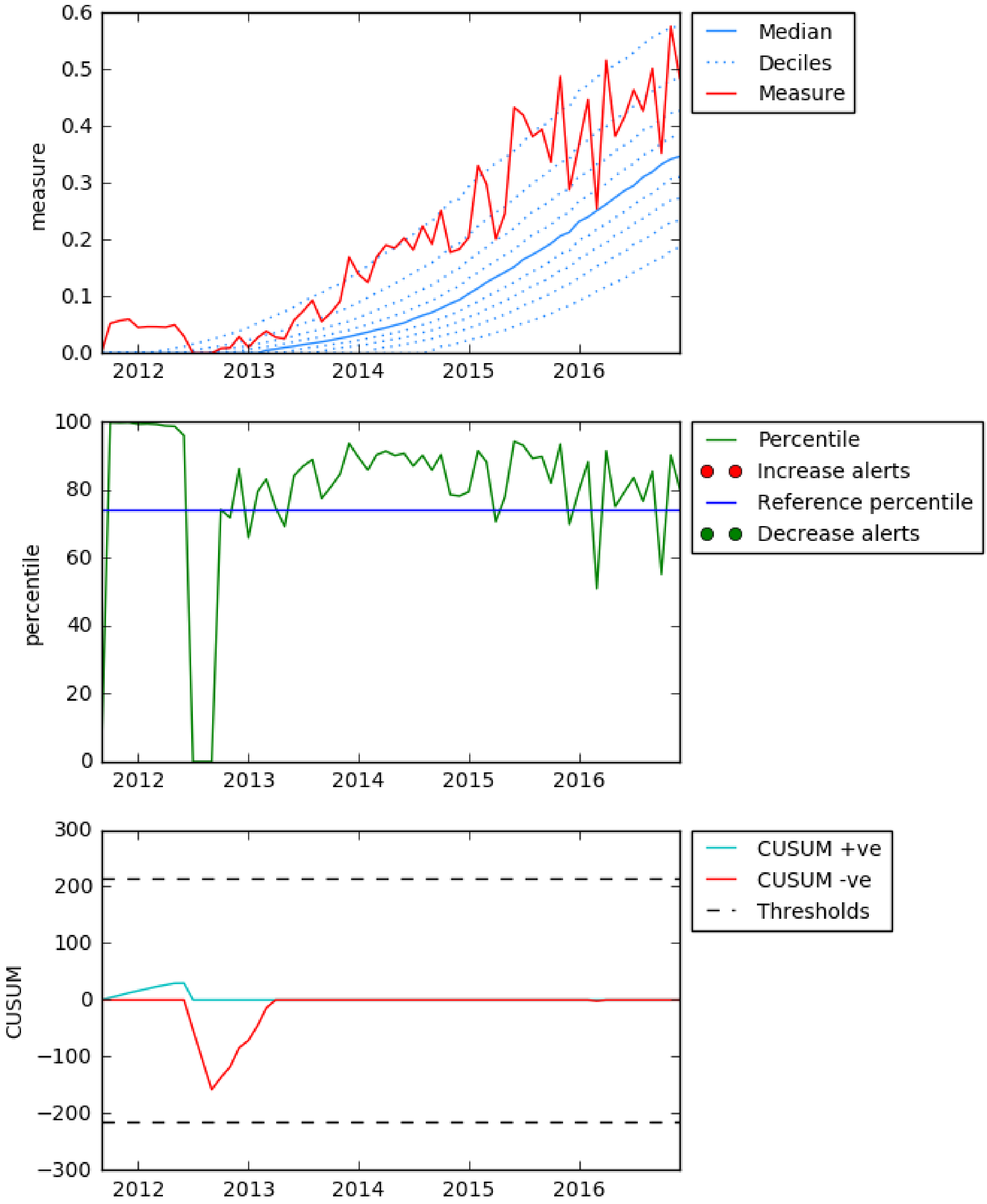

Measure: https://openprescribing.net/ccg/05Y/#opioidper1000

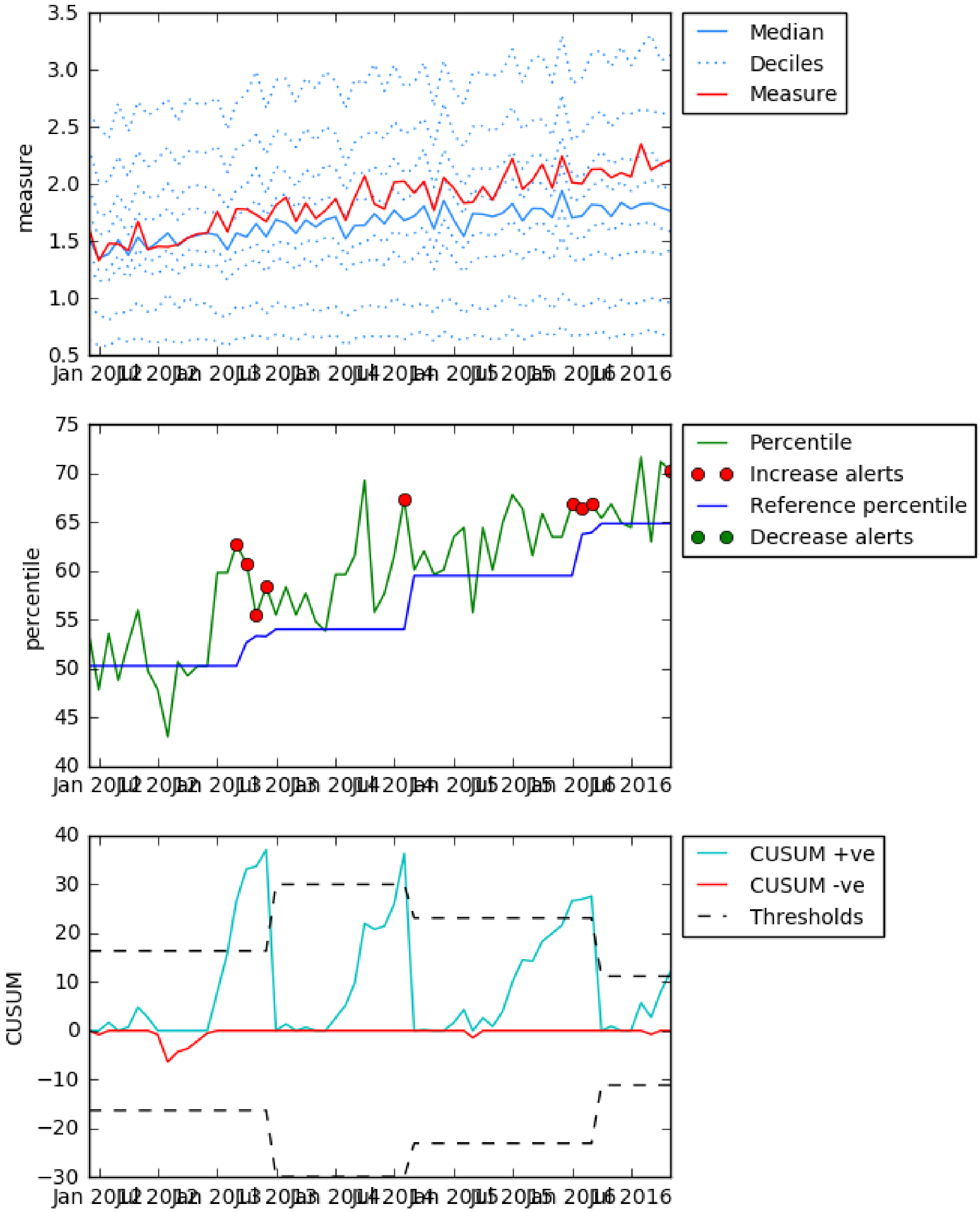

Measure: https://openprescribing.net/practice/G85138/#opioidper1000

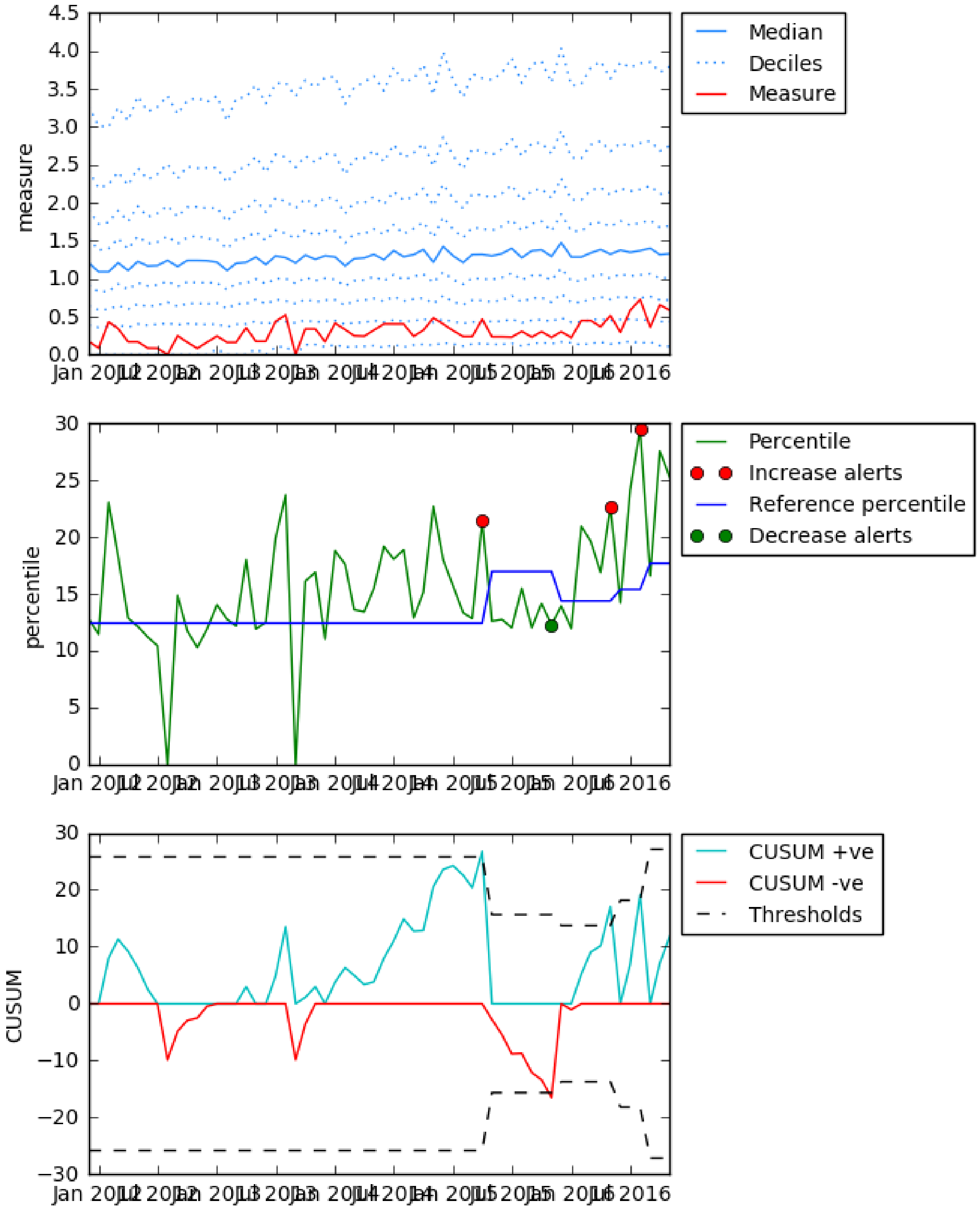

Measure: https://openprescribing.net/ccg/05Y/#ktt12_diabetes_insulin

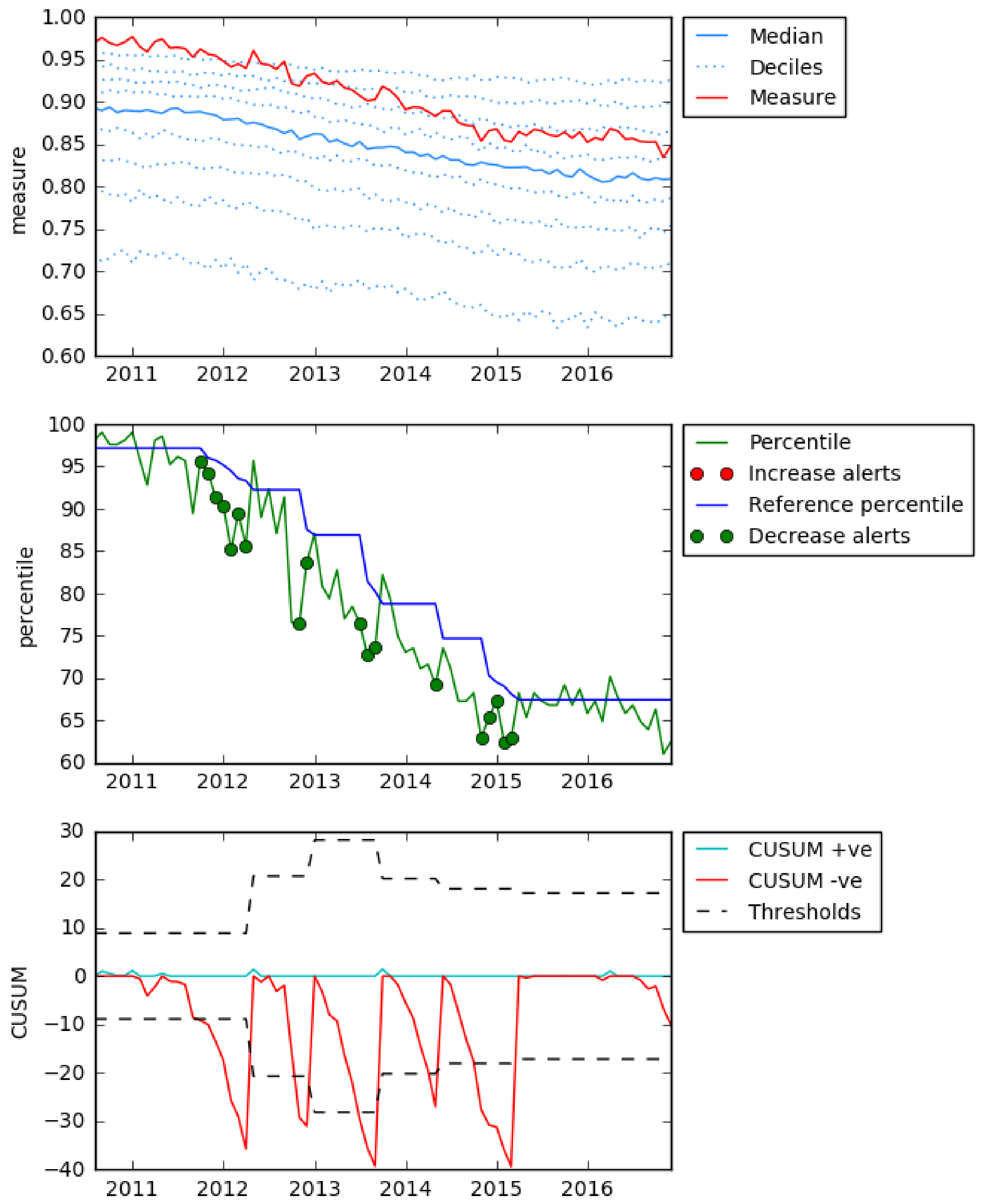

Measure: https://openprescribing.net/practice/G85138/#ktt12_diabetes_insulin

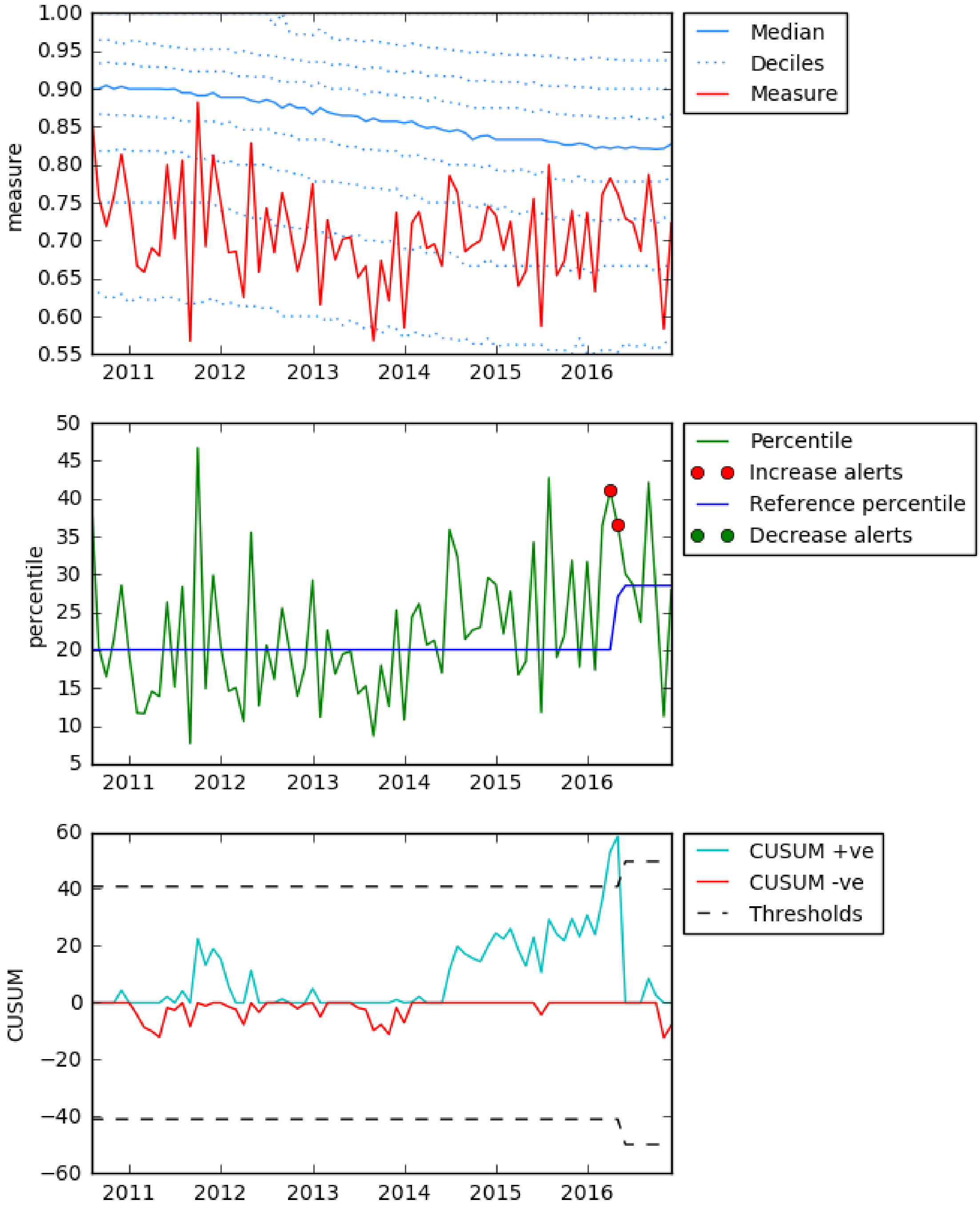

Measure: https://openprescribing.net/ccg/05Y/#ktt9_antibiotics

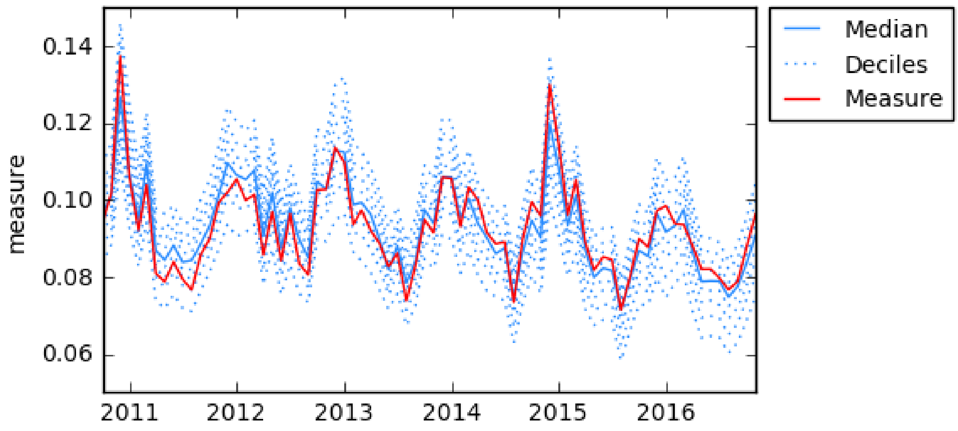

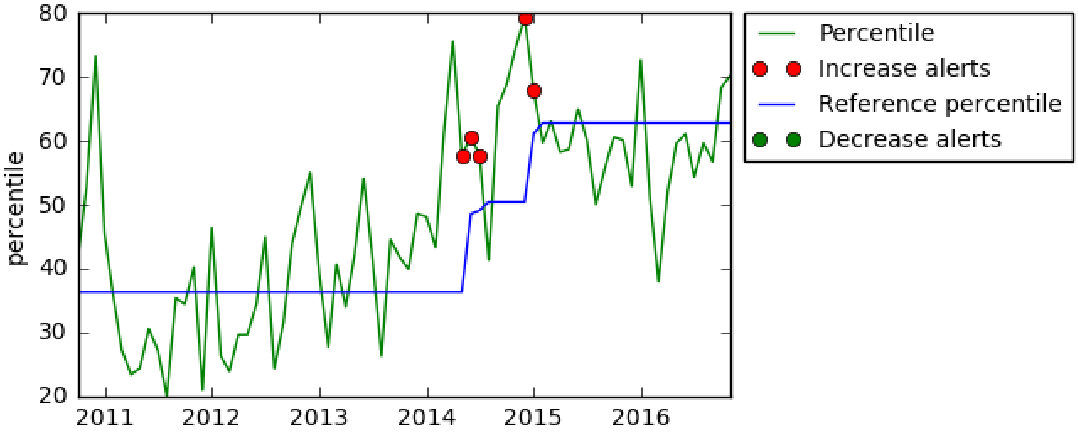

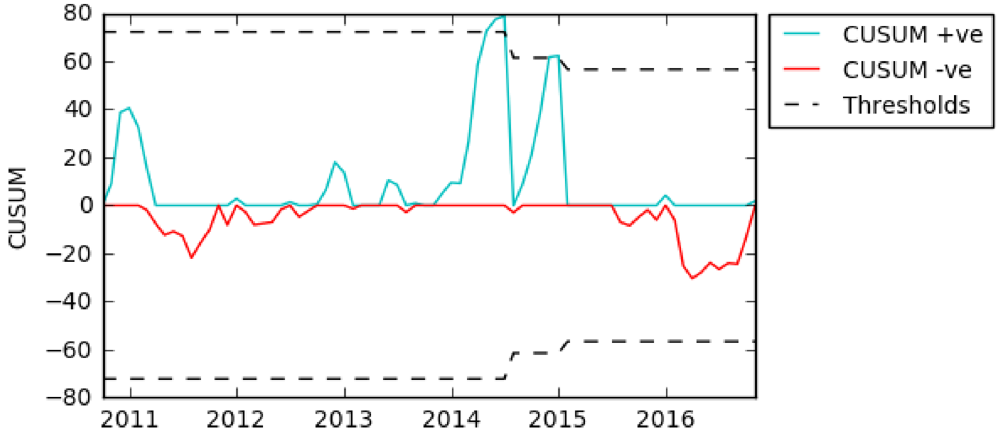

Measure: https://openprescribing.net/practice/G85138/#ktt9_antibiotics

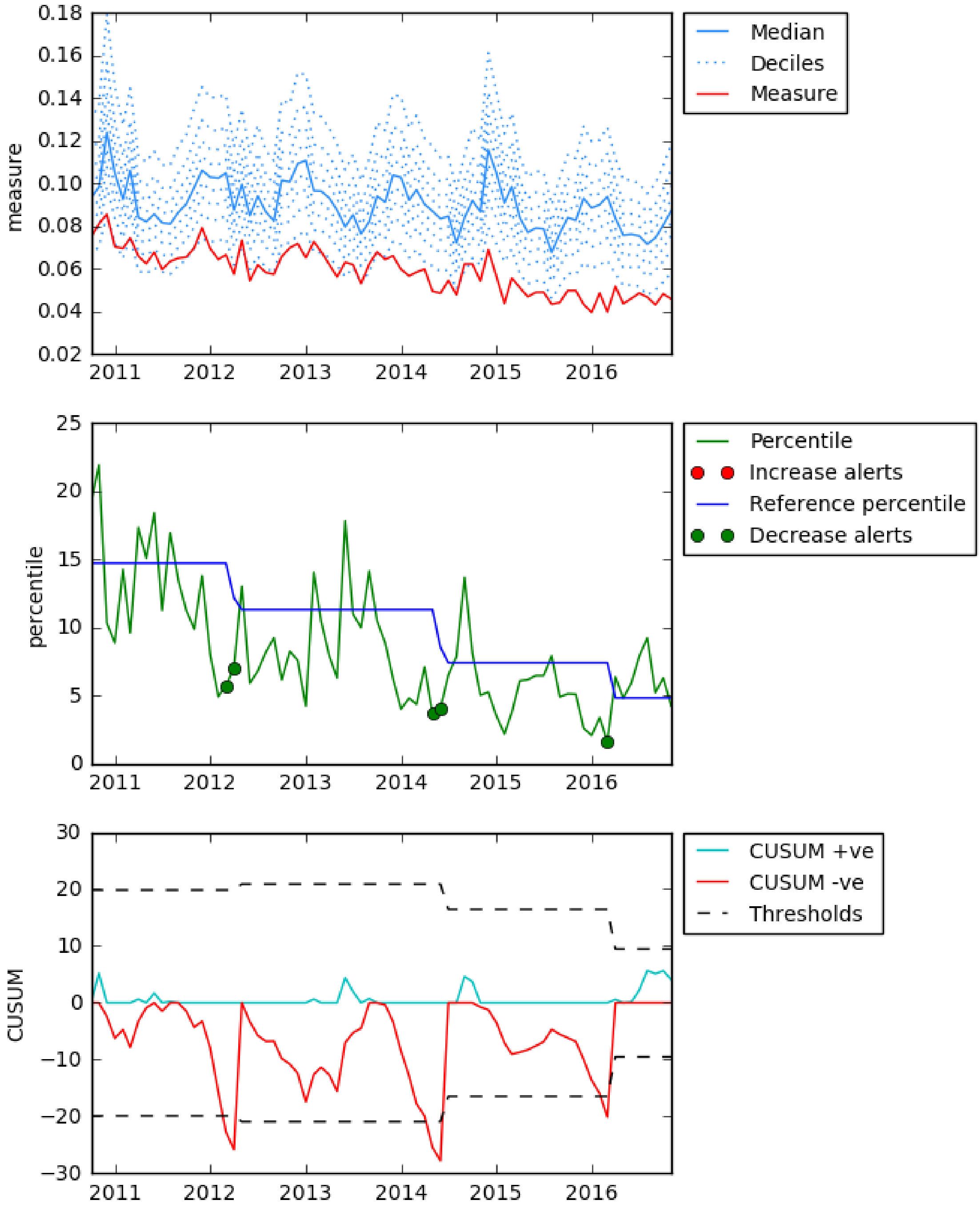

Measure: https://openprescribing.net/ccg/05Y/#pregabalin

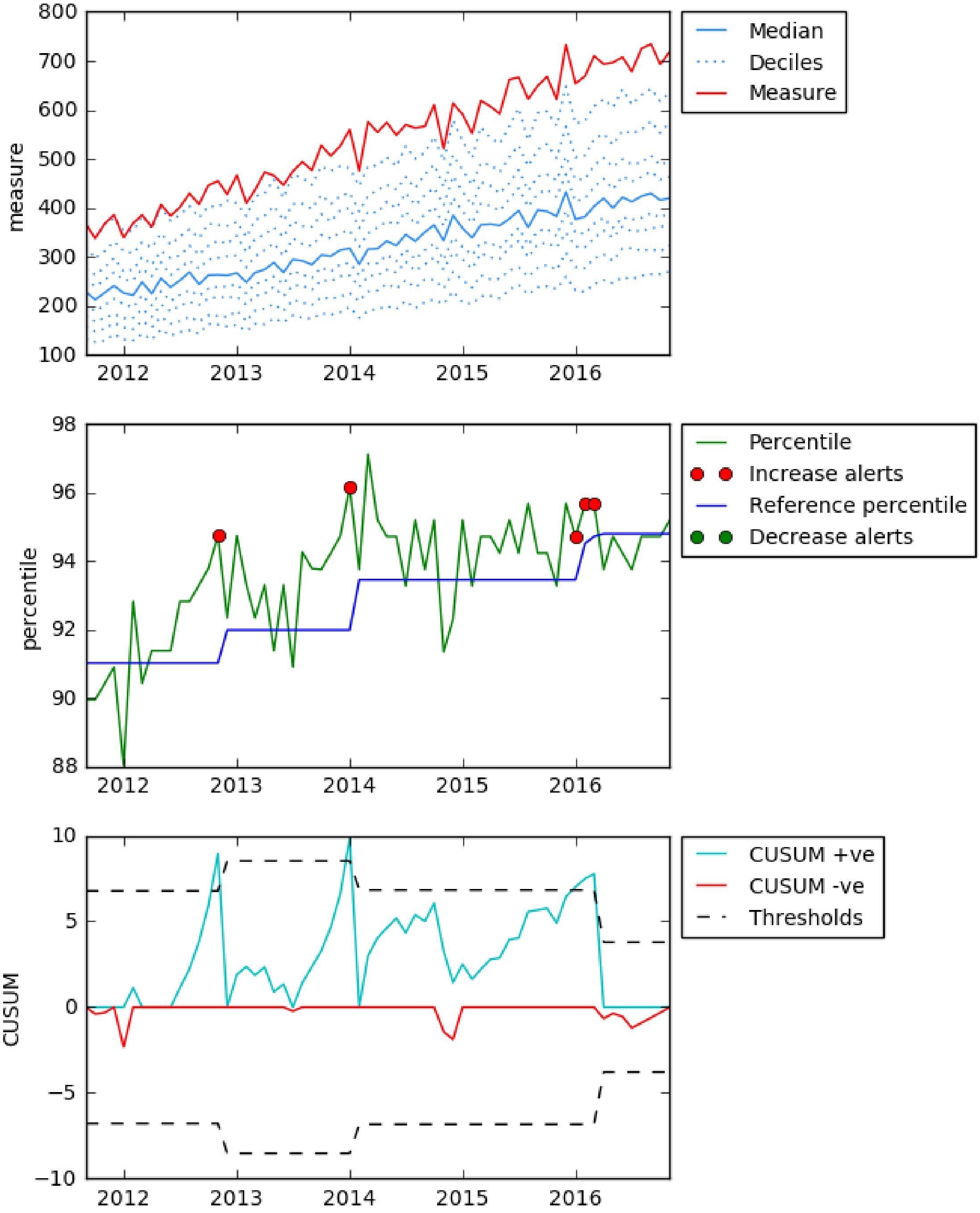

Measure: https://openprescribing.net/practice/G85138/#pregabalin

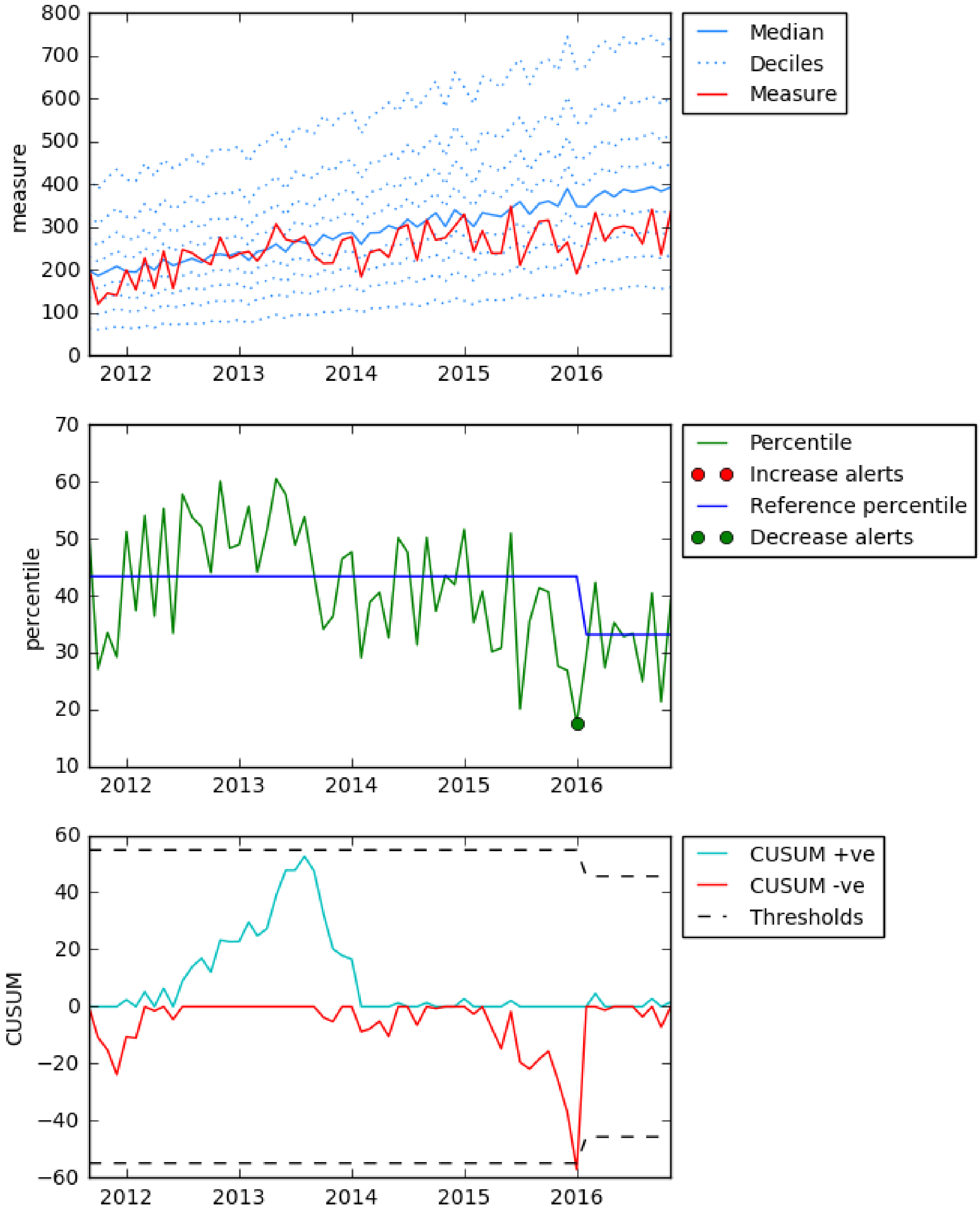

Measure: https://openprescribing.net/ccg/05Y/#diltiazem

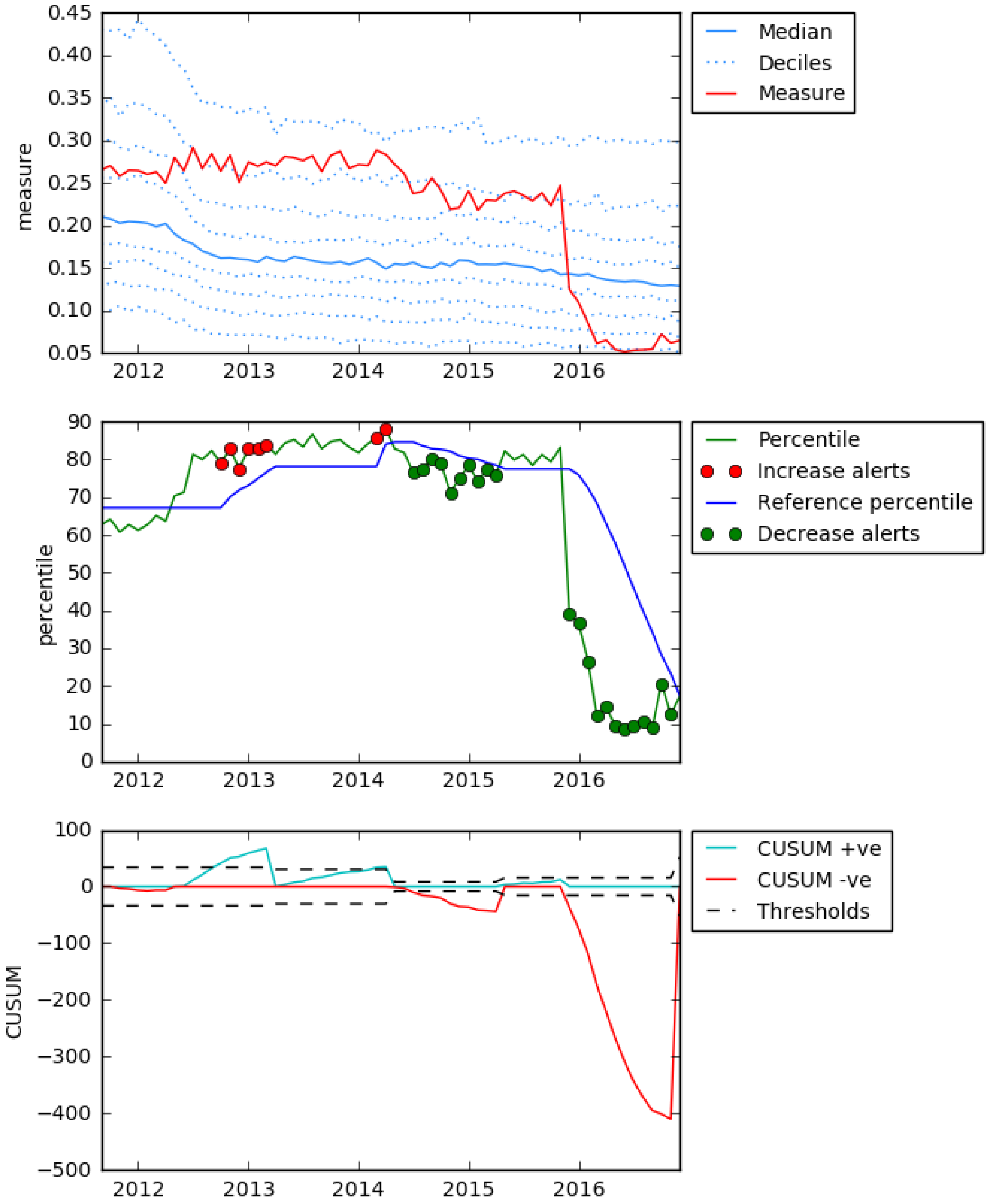

Measure: https://openprescribing.net/practice/G85138/#diltiazem

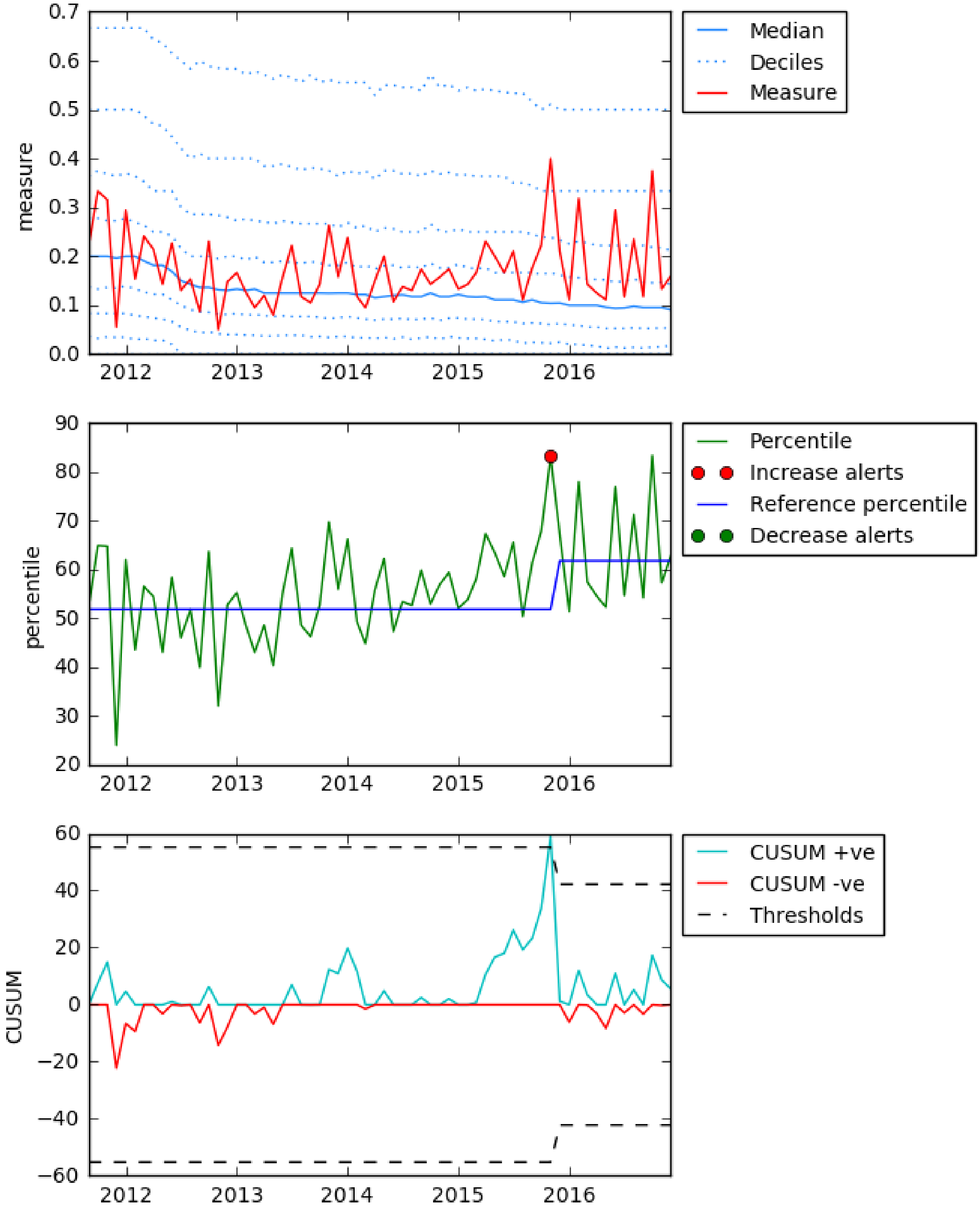

Measure: https://openprescribing.net/ccg/05Y/#coproxamol

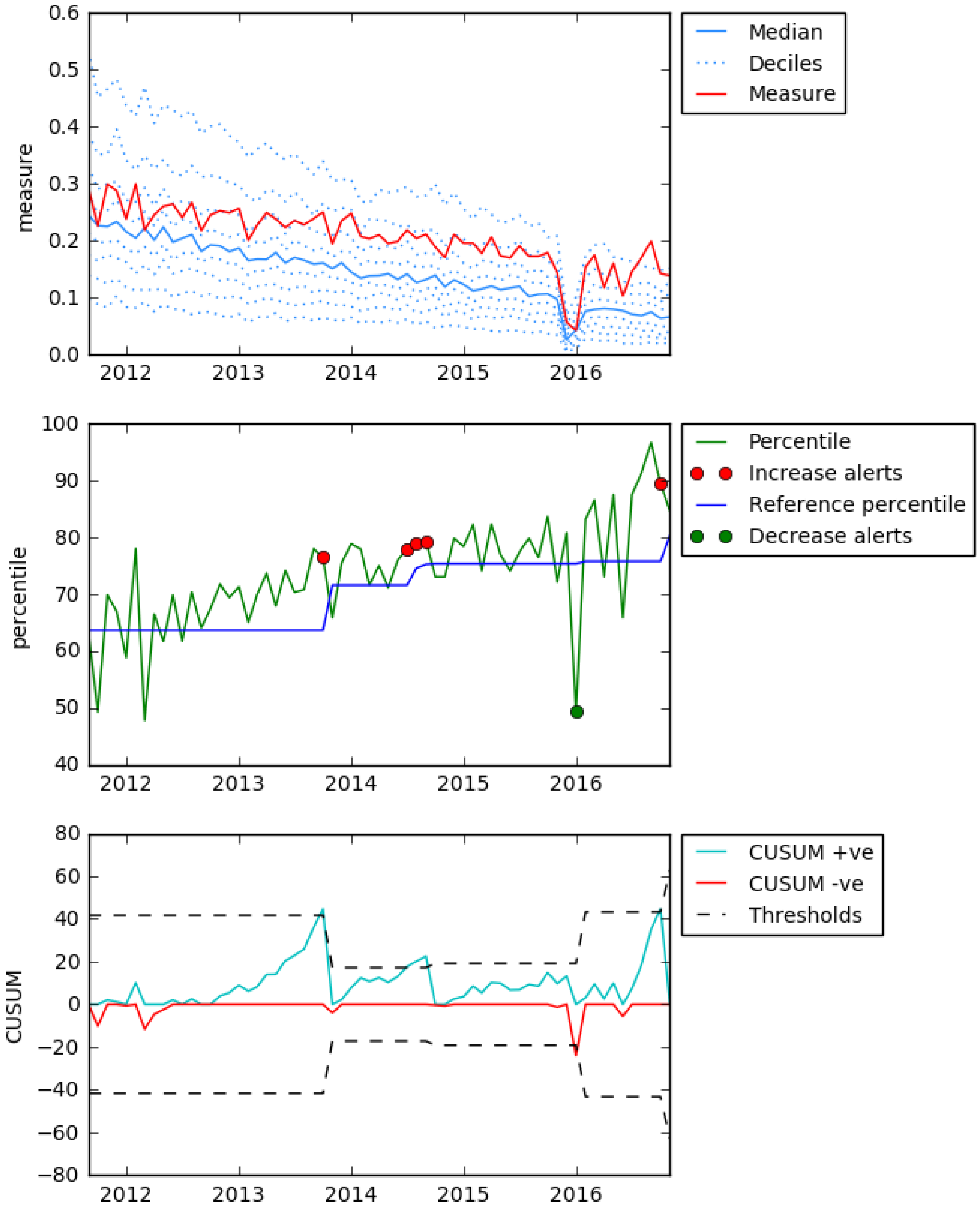

Measure: https://openprescribing.net/practice/G85138/#coproxamol

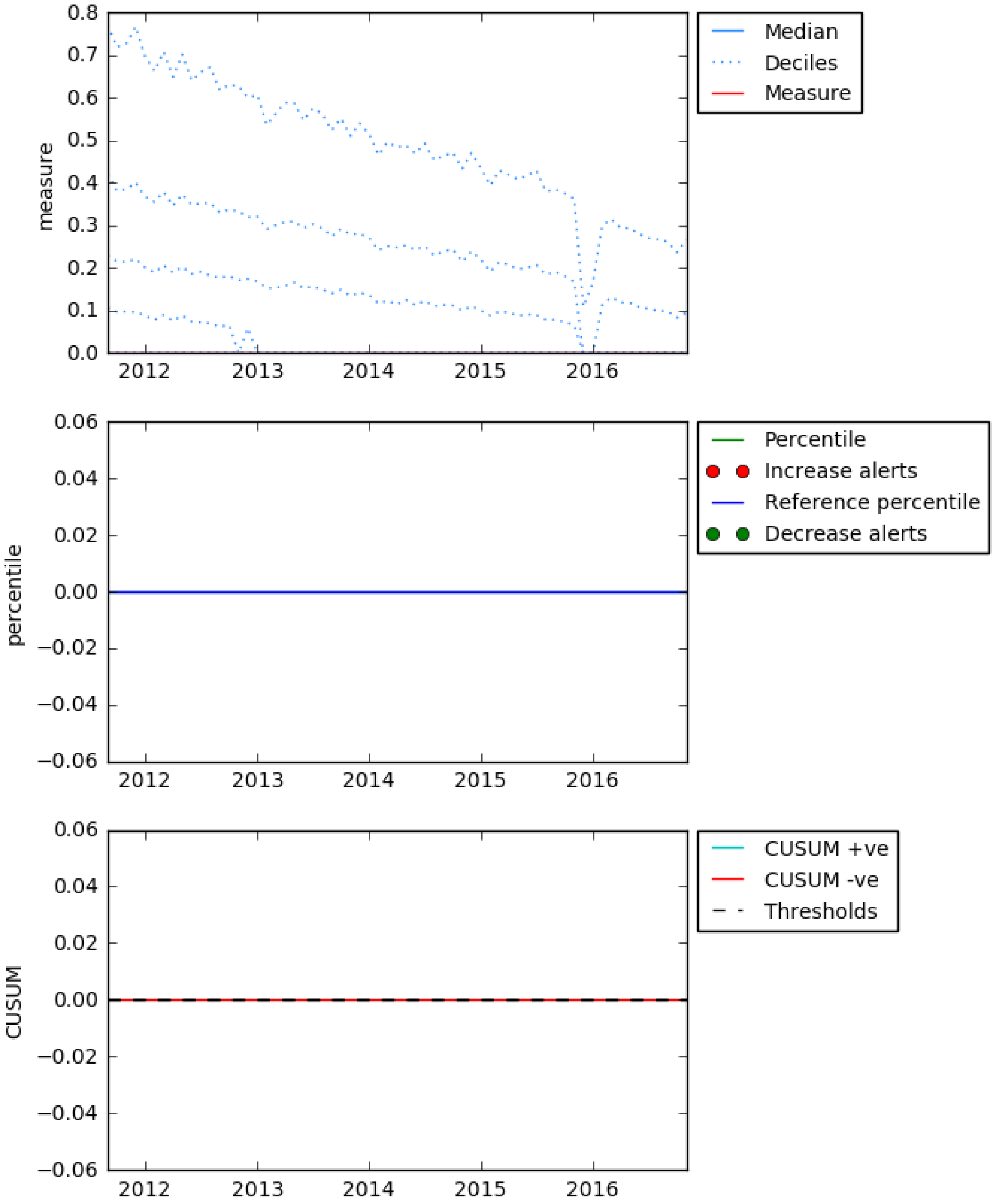

Measure: https://openprescribing.net/ccg/05Y/#opiates

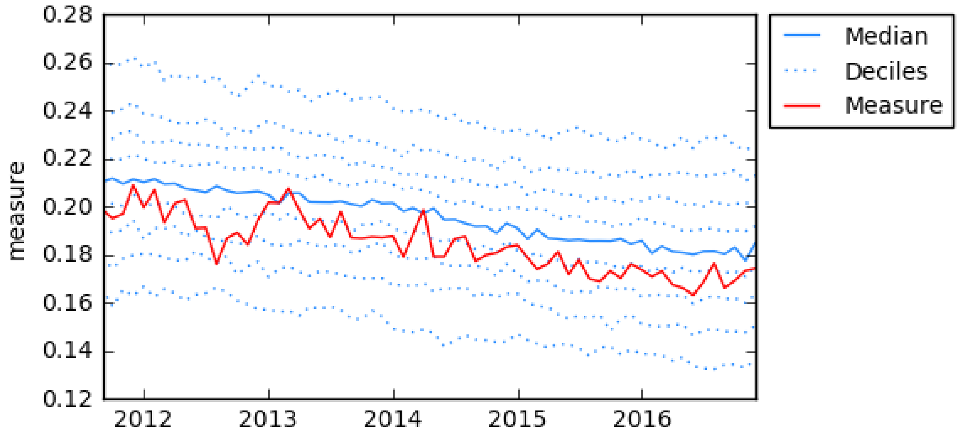

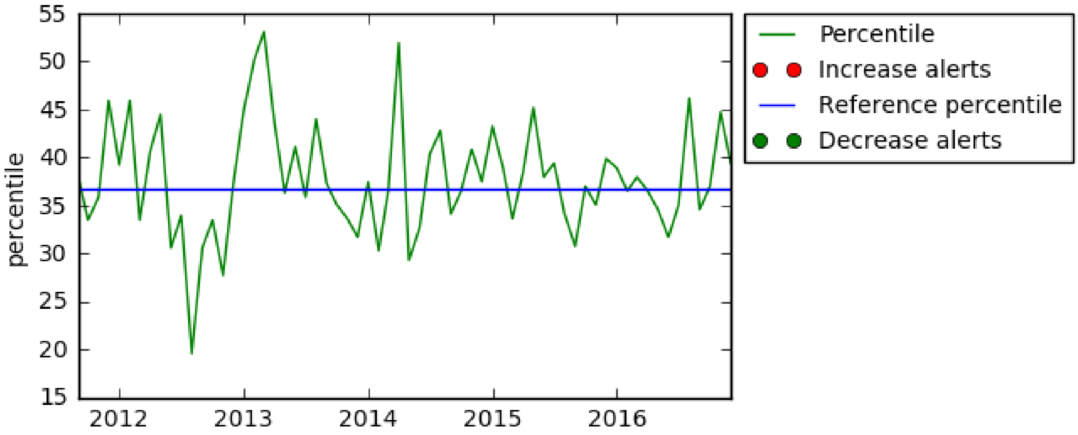

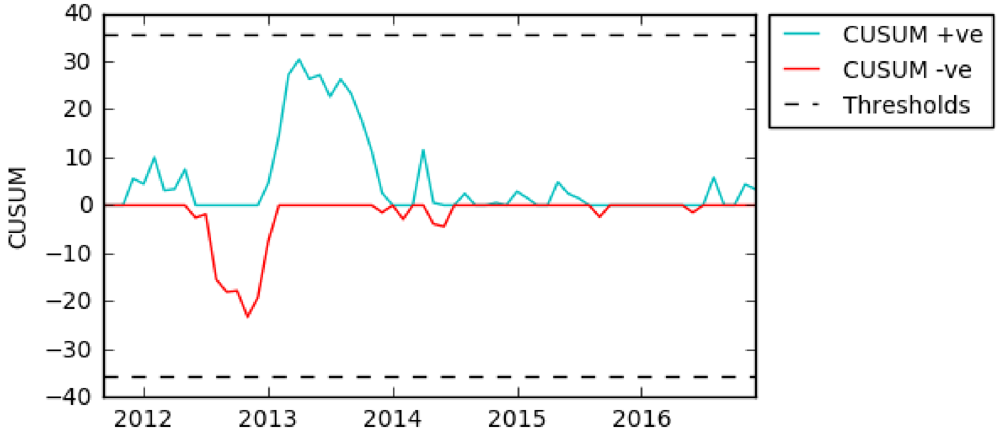

Measure: https://openprescribing.net/practice/G85138/#opiates

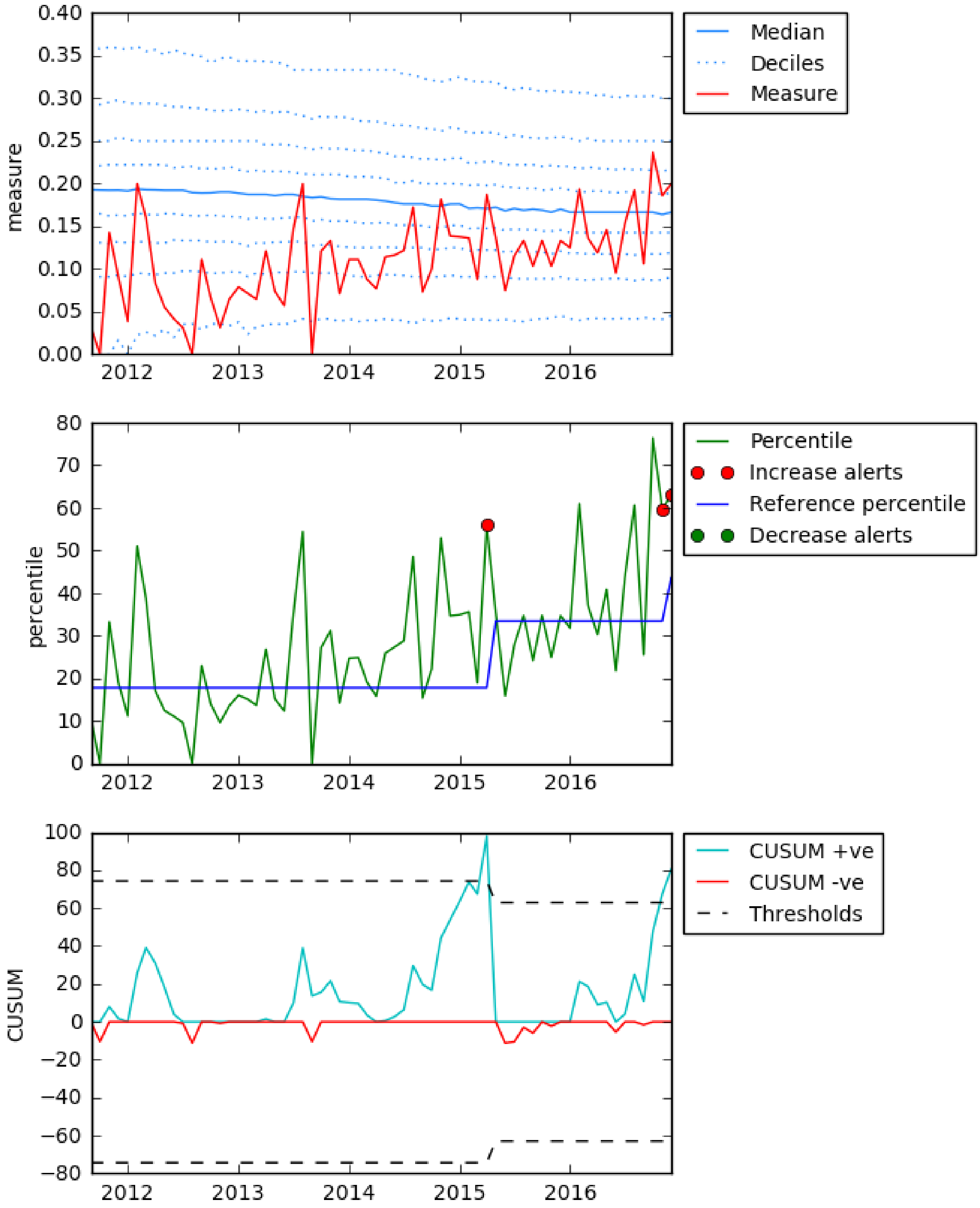

Measure: https://openprescribing.net/ccg/05Y/#ciclosporin

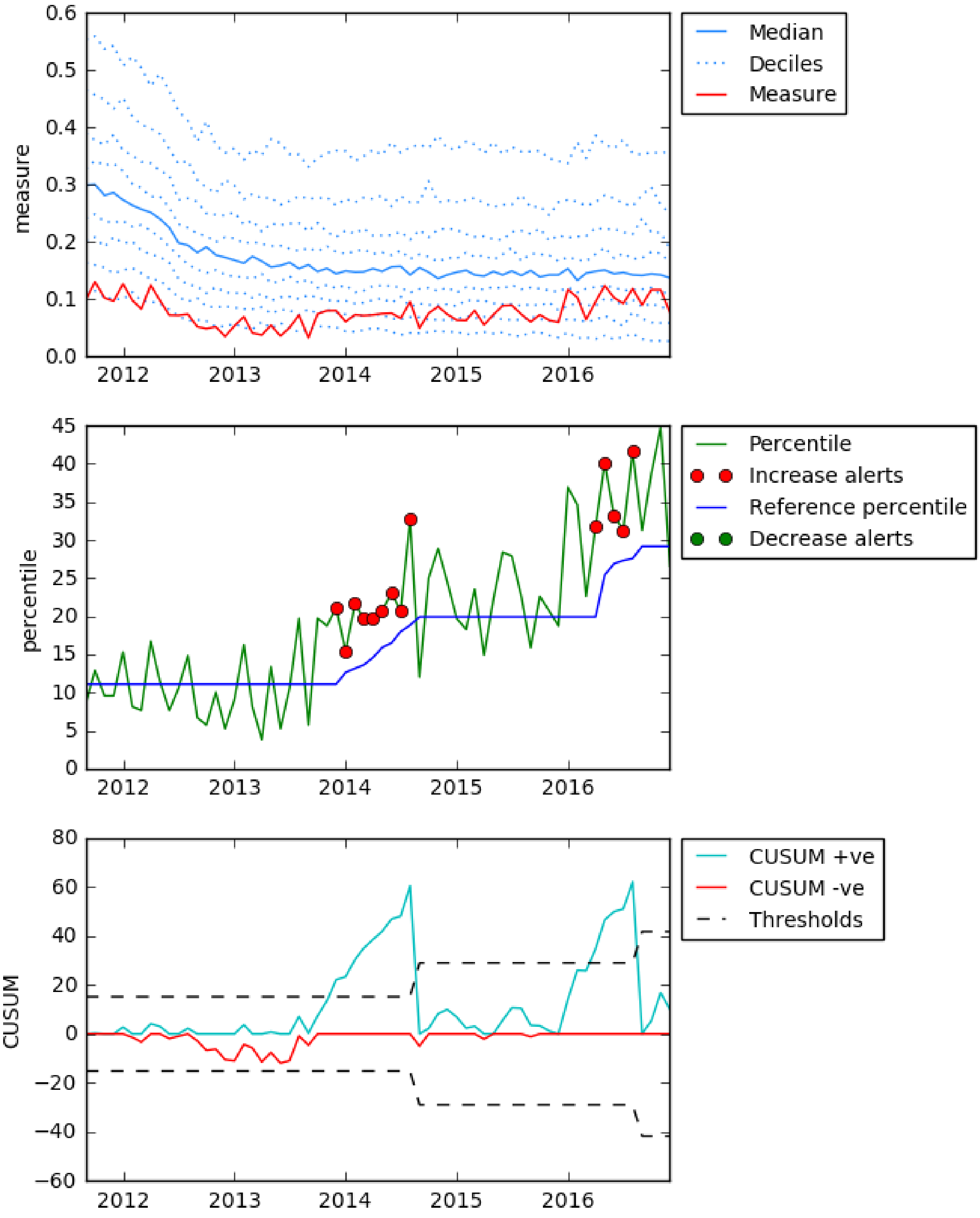

Measure: https://openprescribing.net/practice/G85138/#ciclosporin

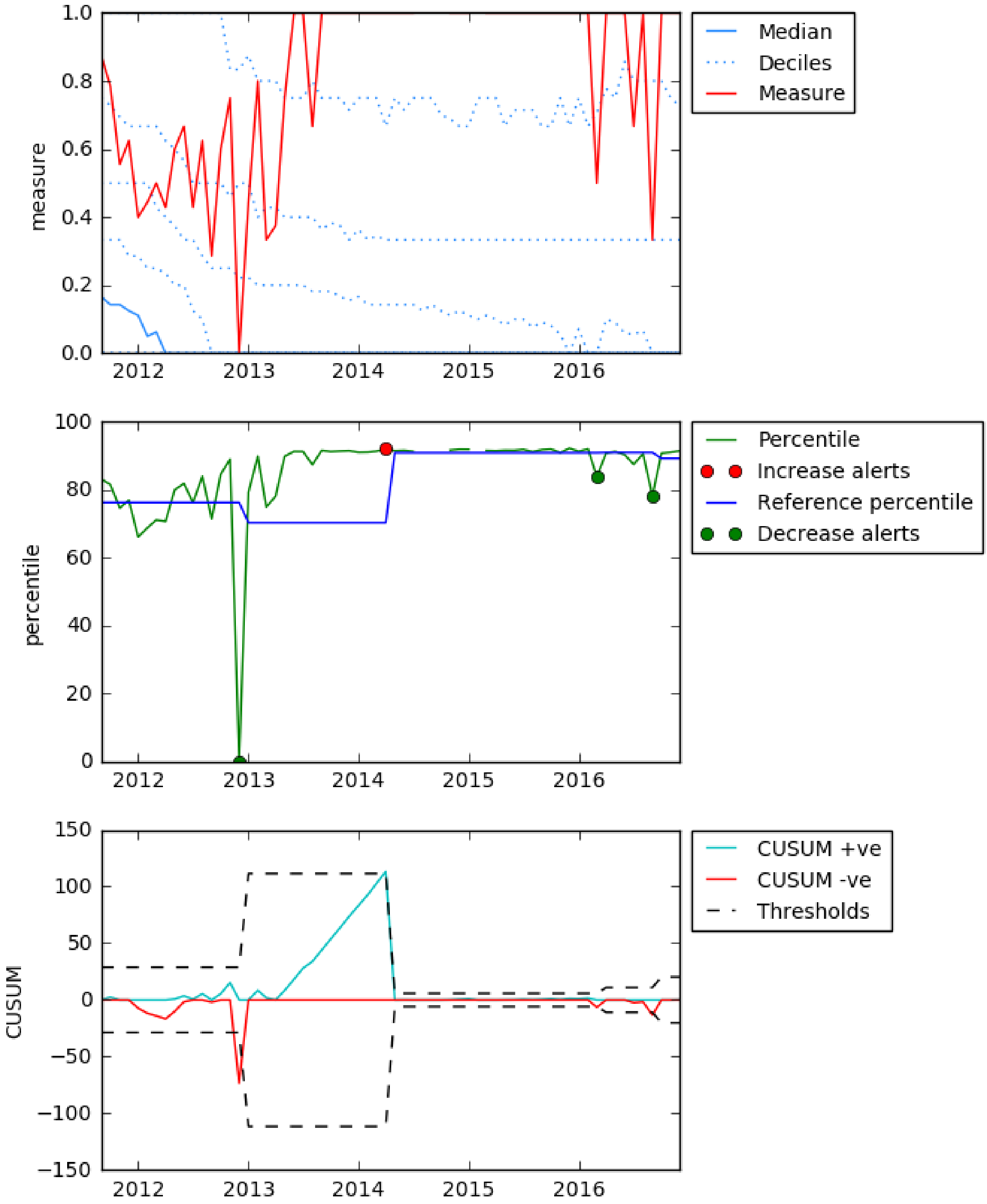

Measure: https://openprescribing.net/ccg/05Y/#cerazette

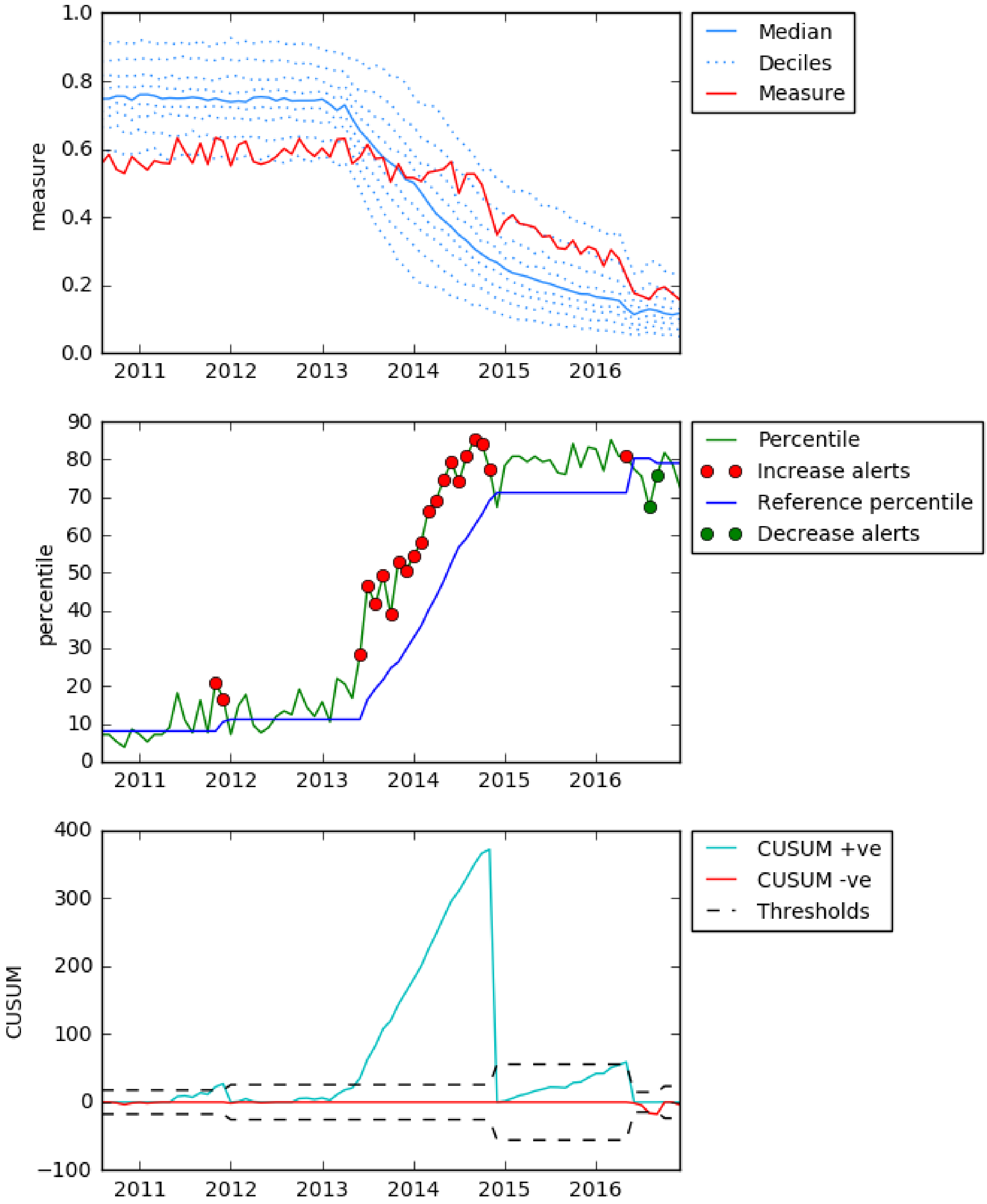

Measure: https://openprescribing.net/practice/G85138/#cerazette

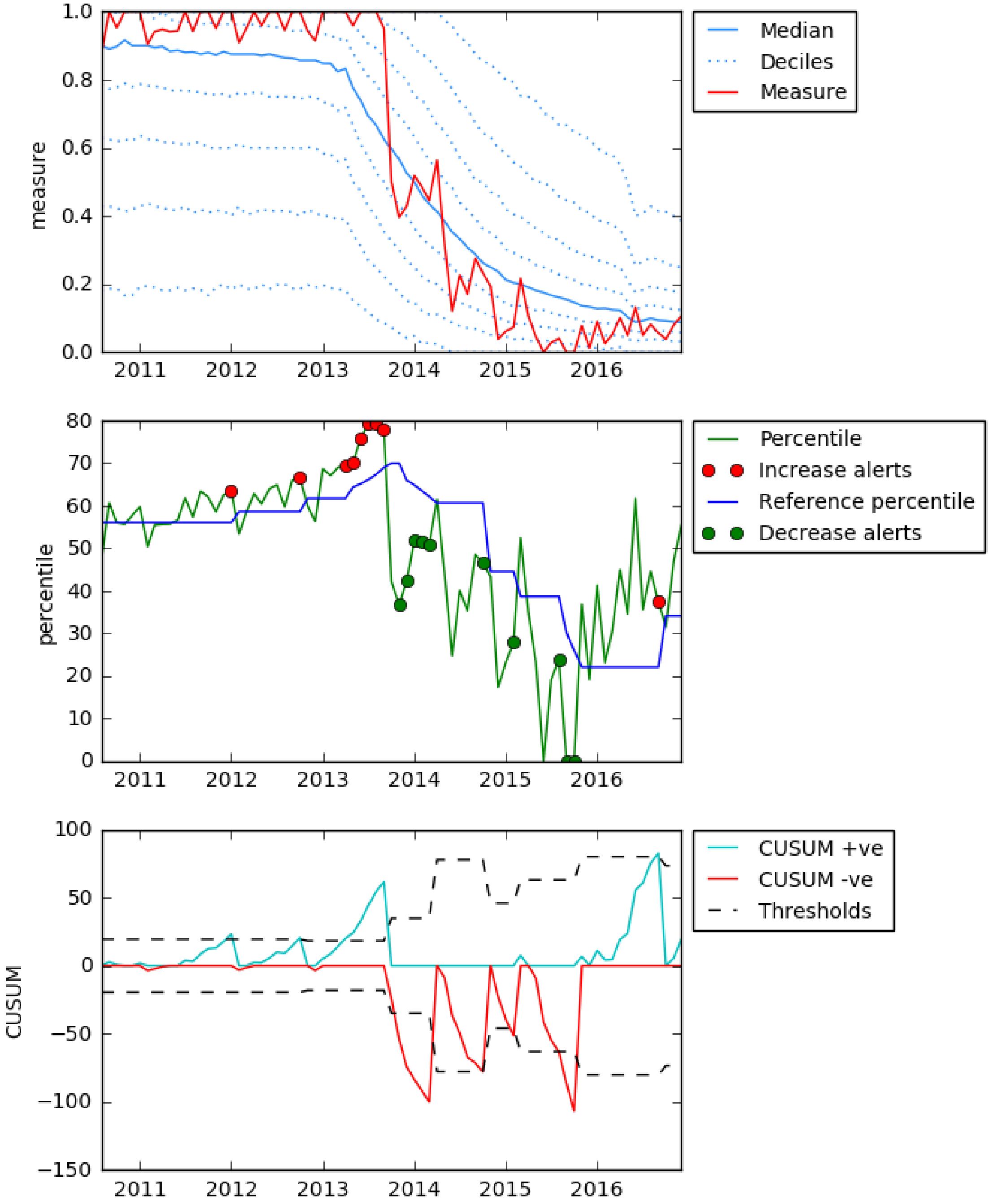

Measure: https://openprescribing.net/ccg/05Y/#nebivolol

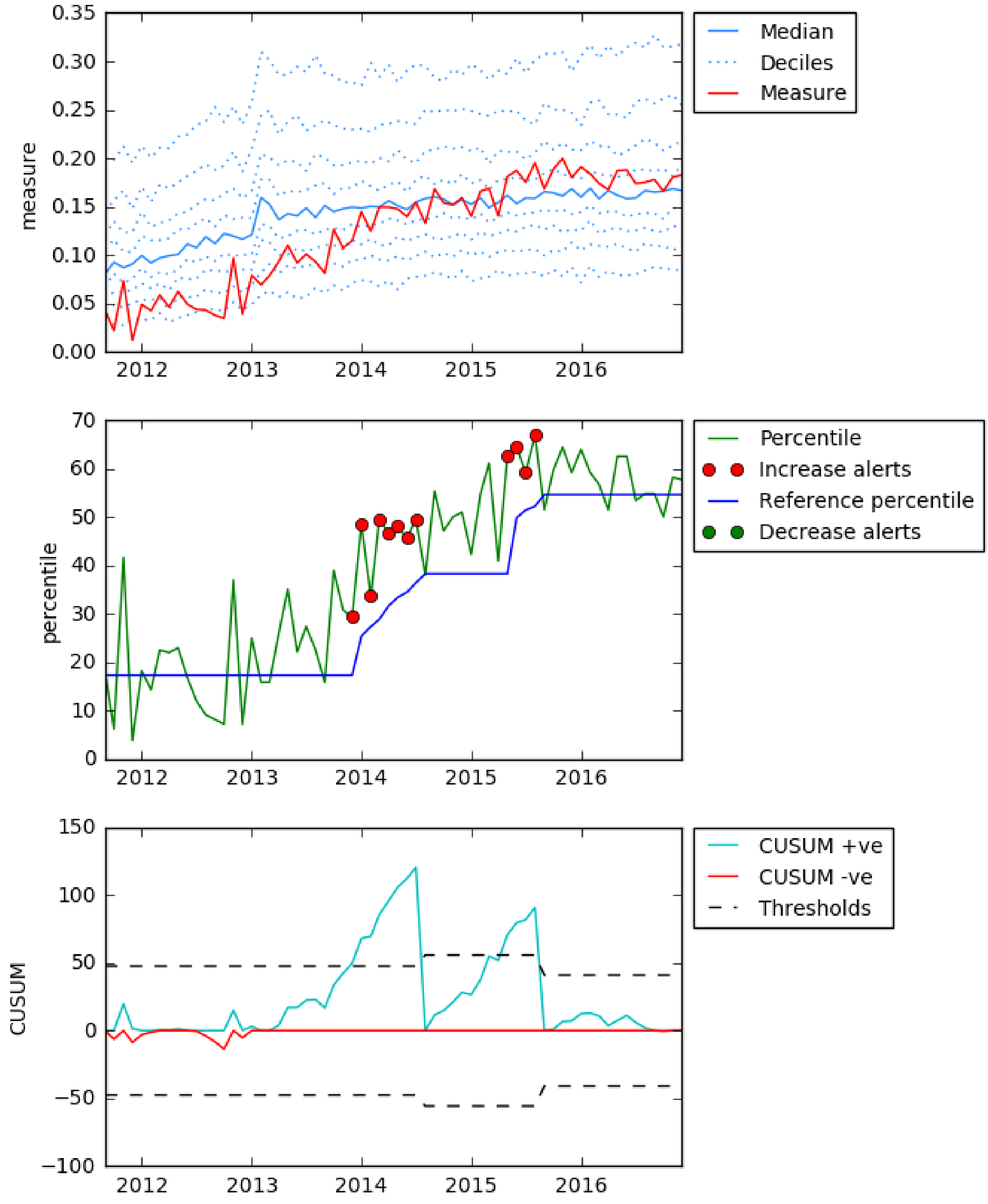

Measure: https://openprescribing.net/practice/G85138/#nebivolol

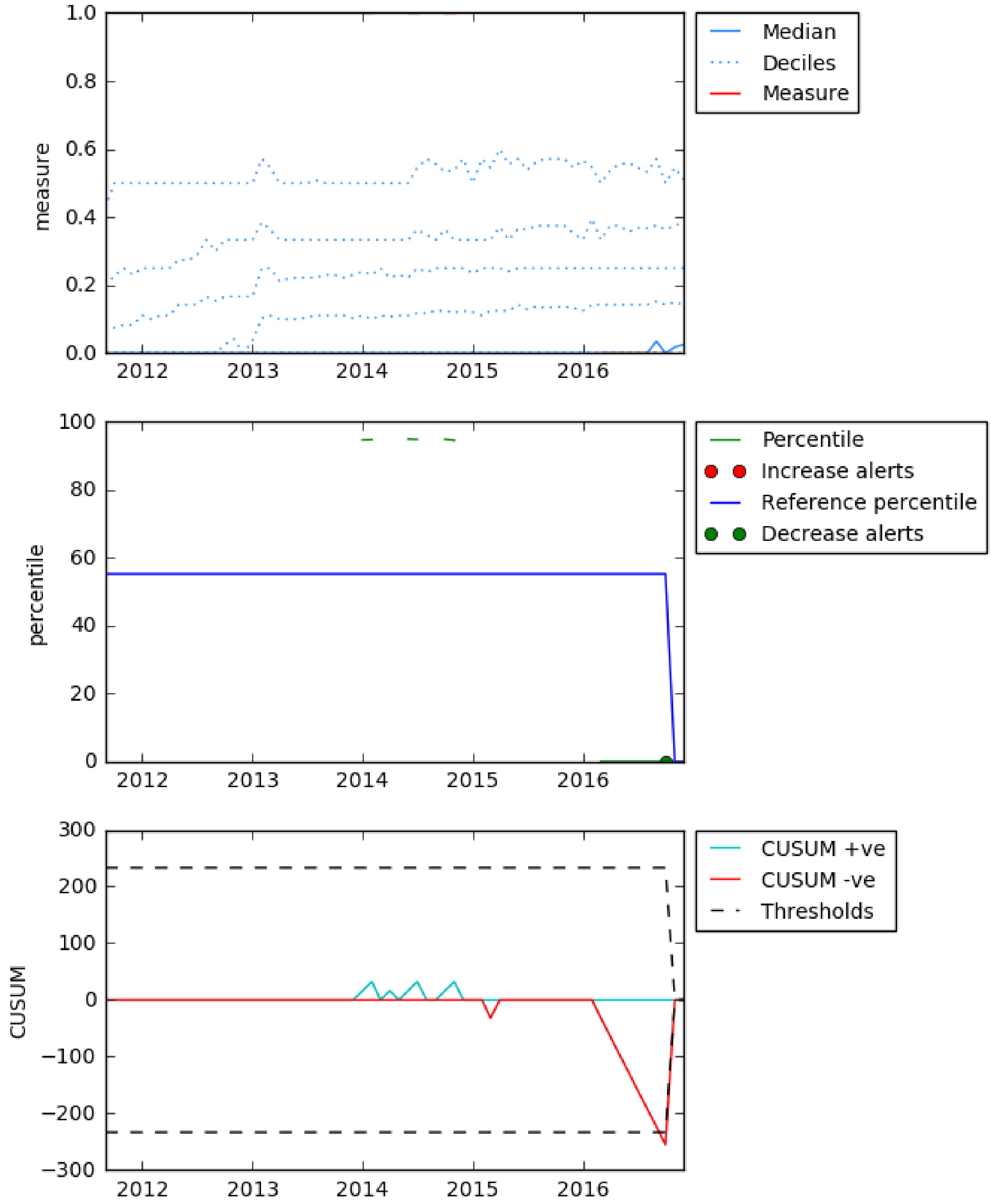

Measure: https://openprescribing.net/ccg/05Y/#ktt9_uti_antibiotics

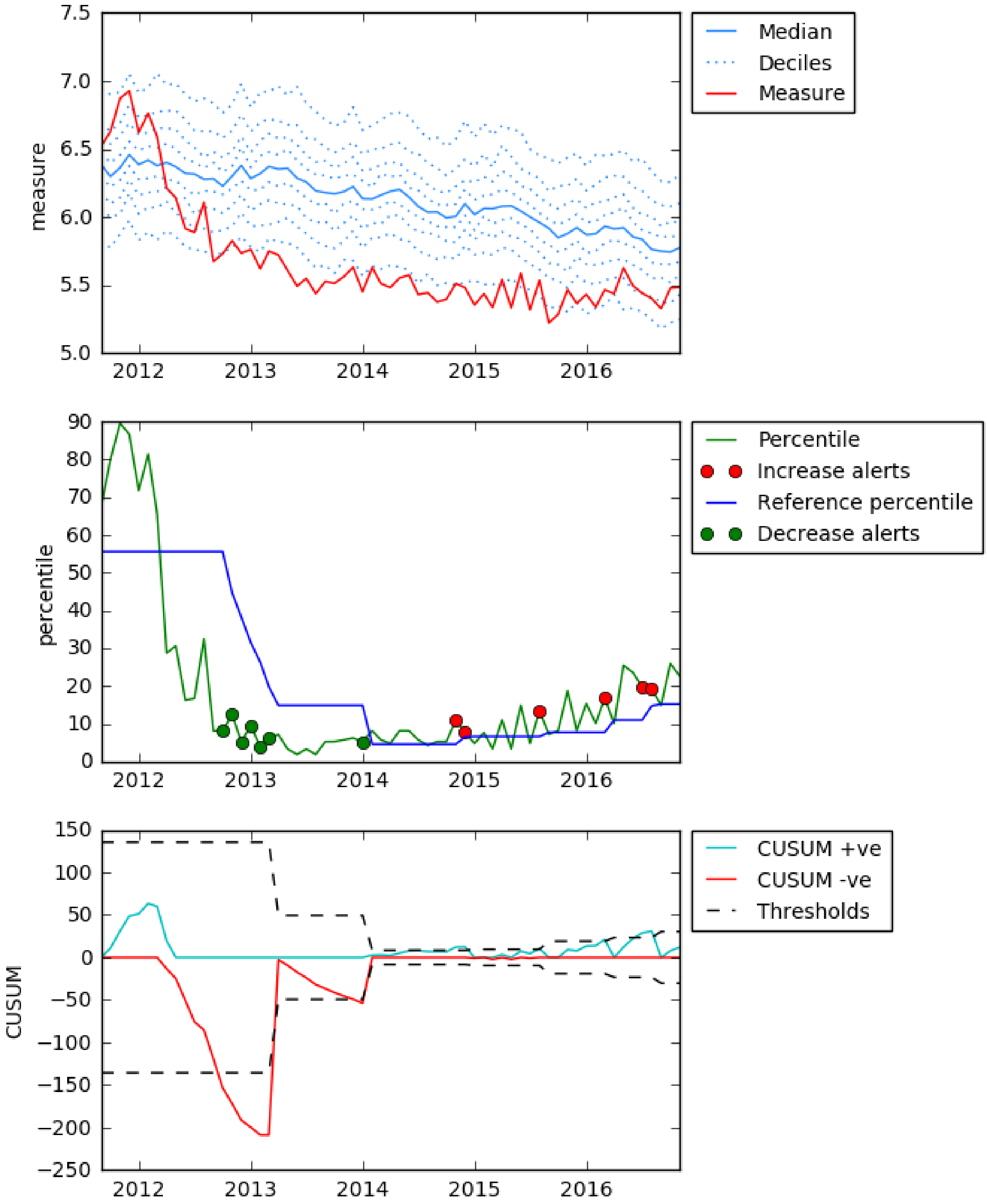

Measure: https://openprescribing.net/practice/G85138/#ktt9_uti_antibiotics

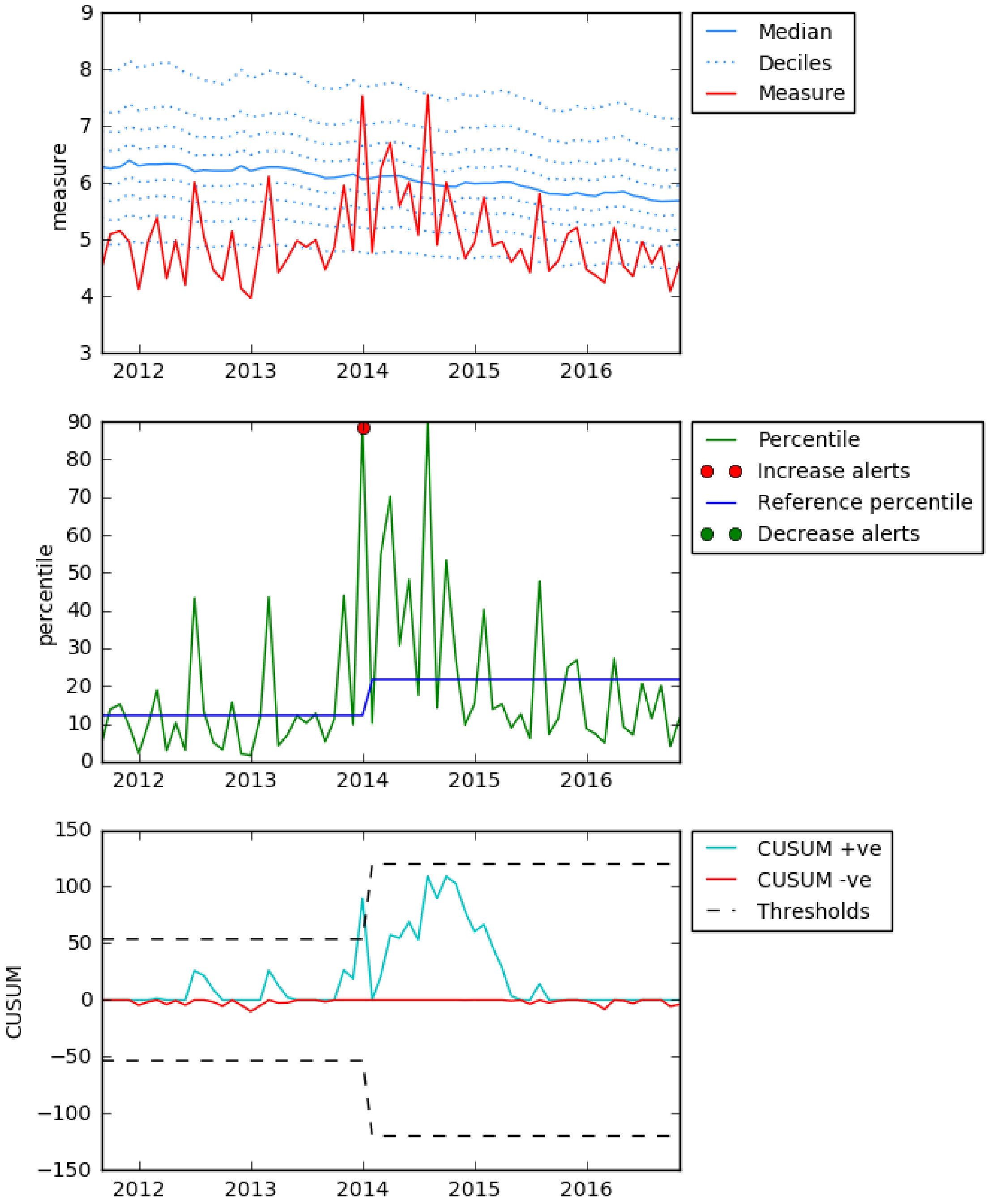

Measure: https://openprescribing.net/ccg/05Y/#ace

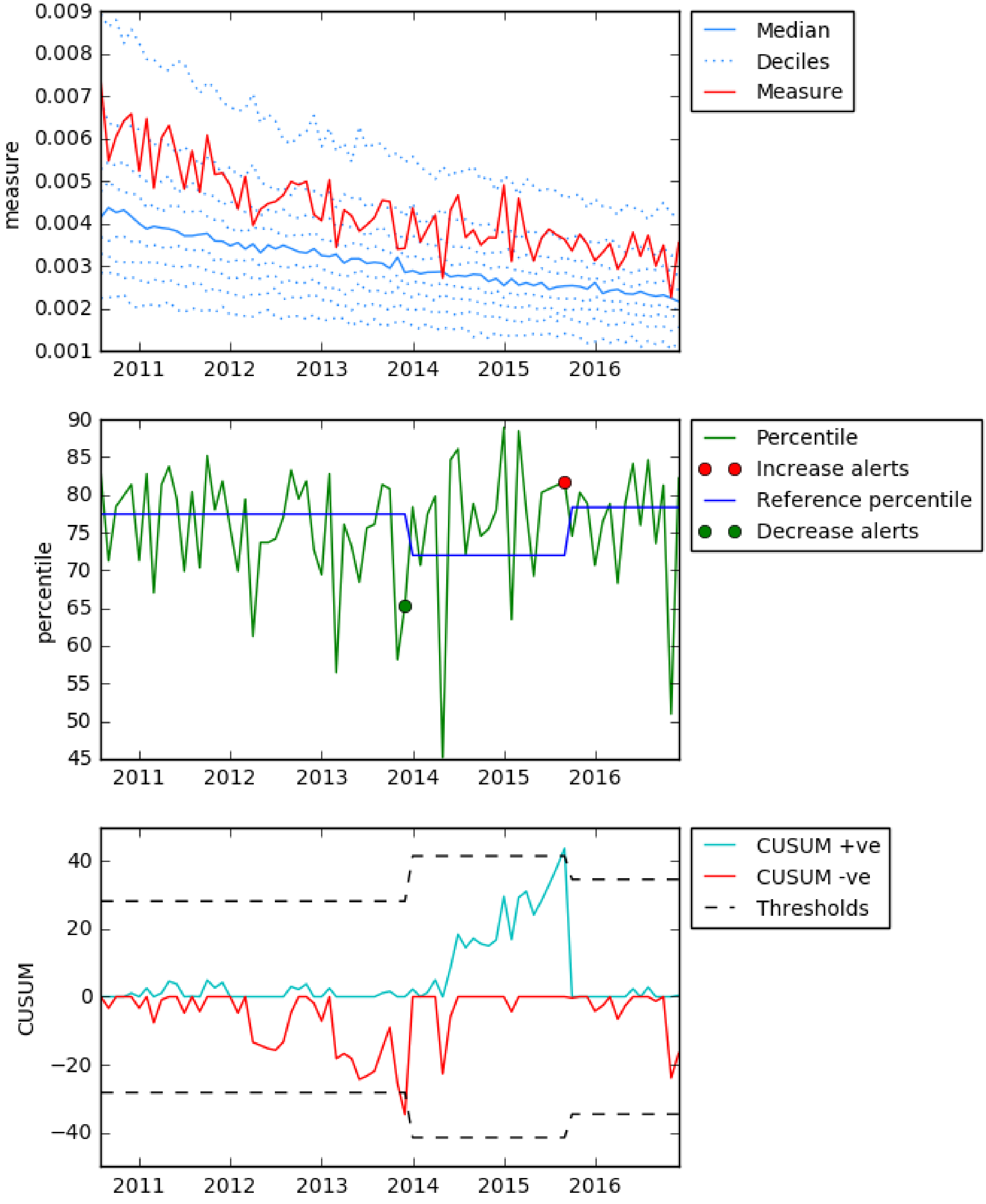

Measure: https://openprescribing.net/practice/G85138/#ace

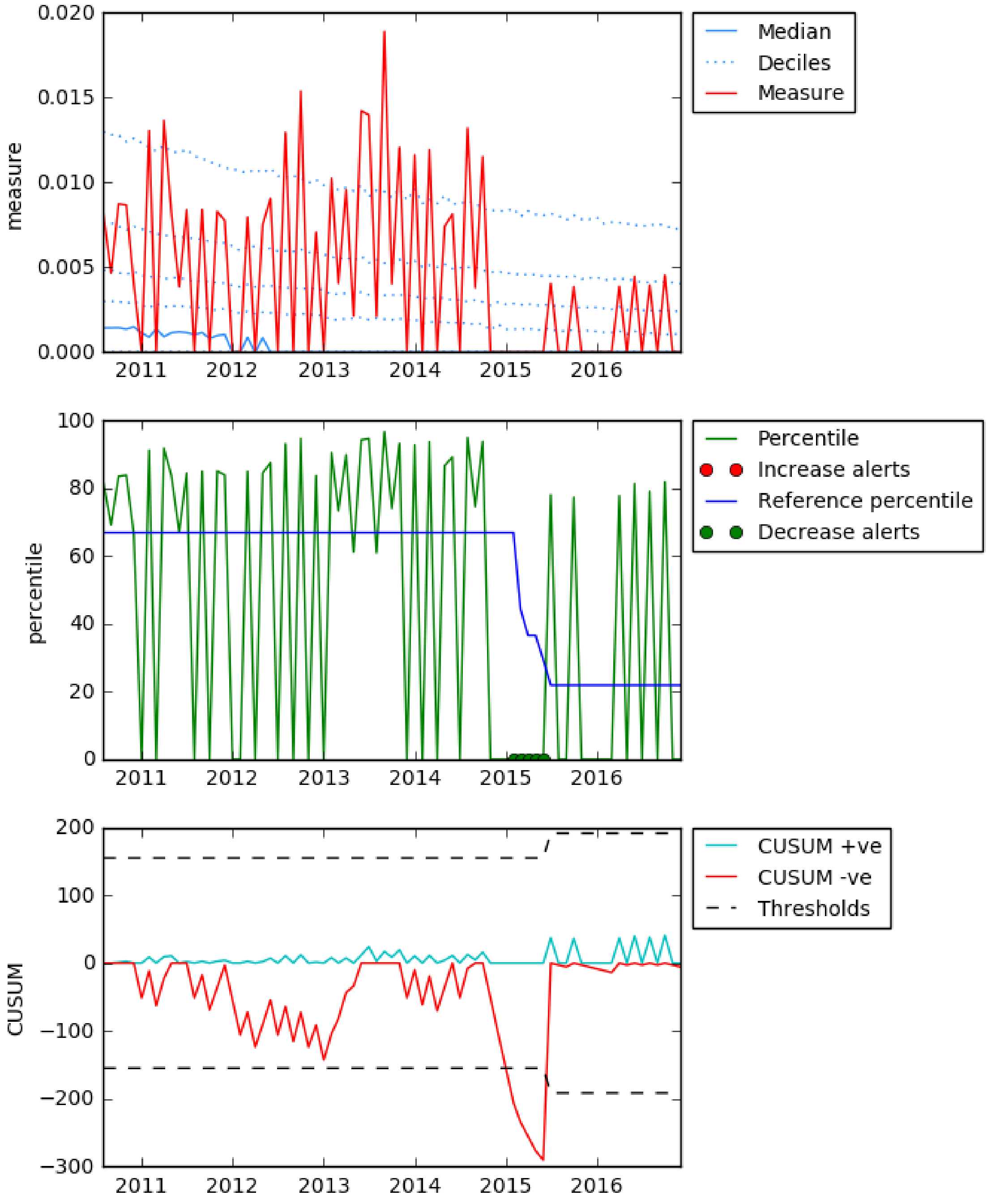

Measure: https://openprescribing.net/ccg/05Y/#statins

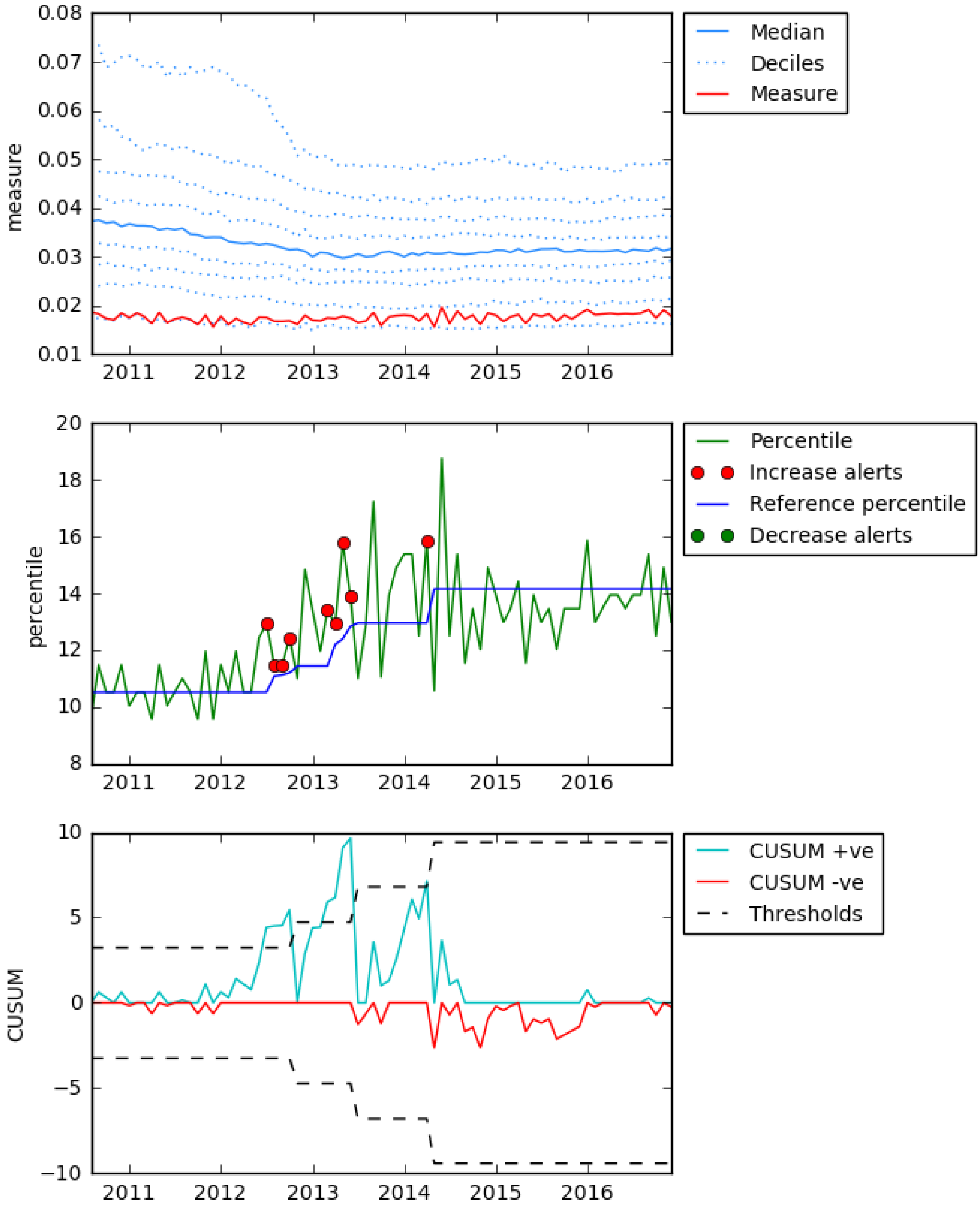

Measure: https://openprescribing.net/practice/G85138/#statins

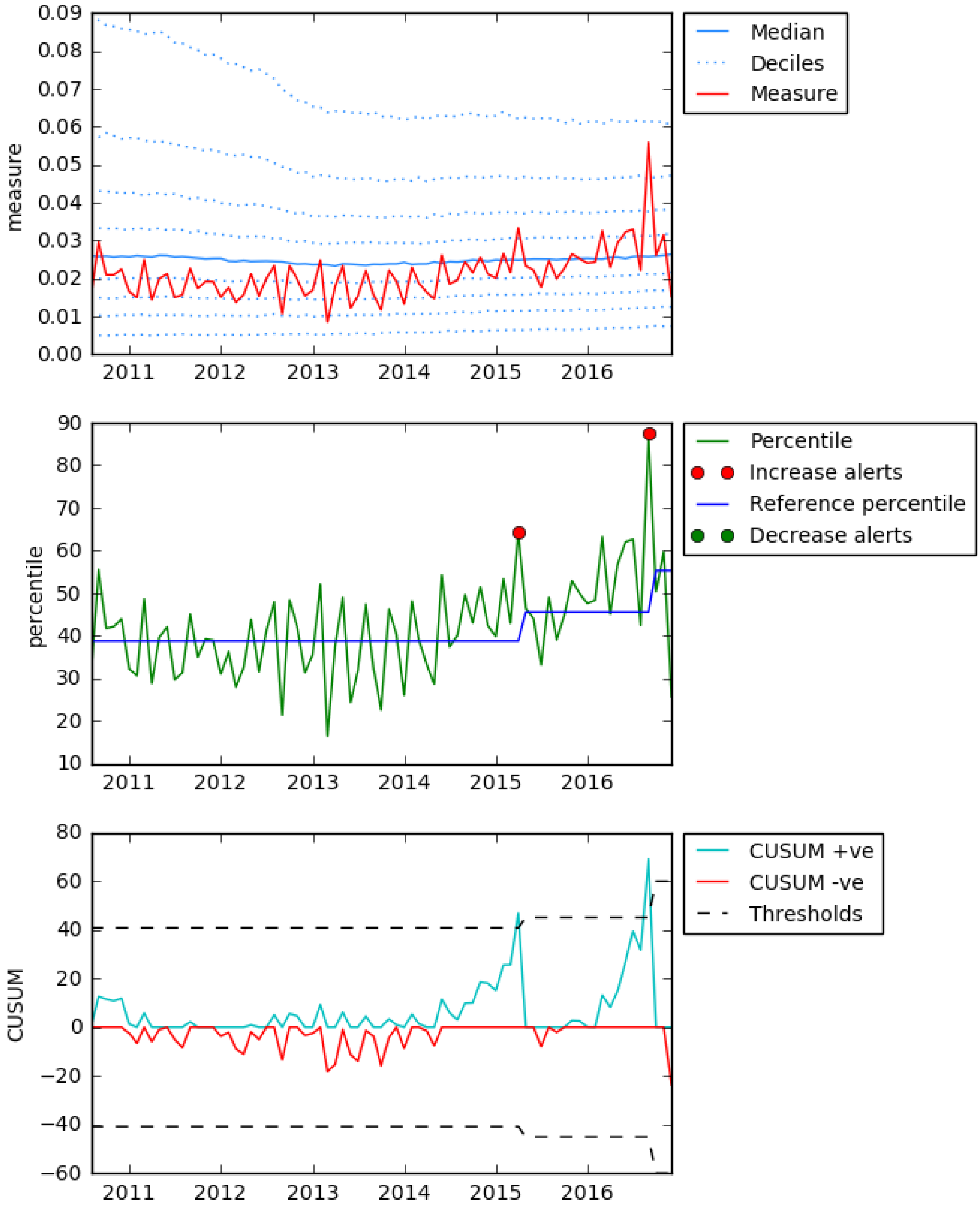

Measure: https://openprescribing.net/ccg/05Y/#glaucoma

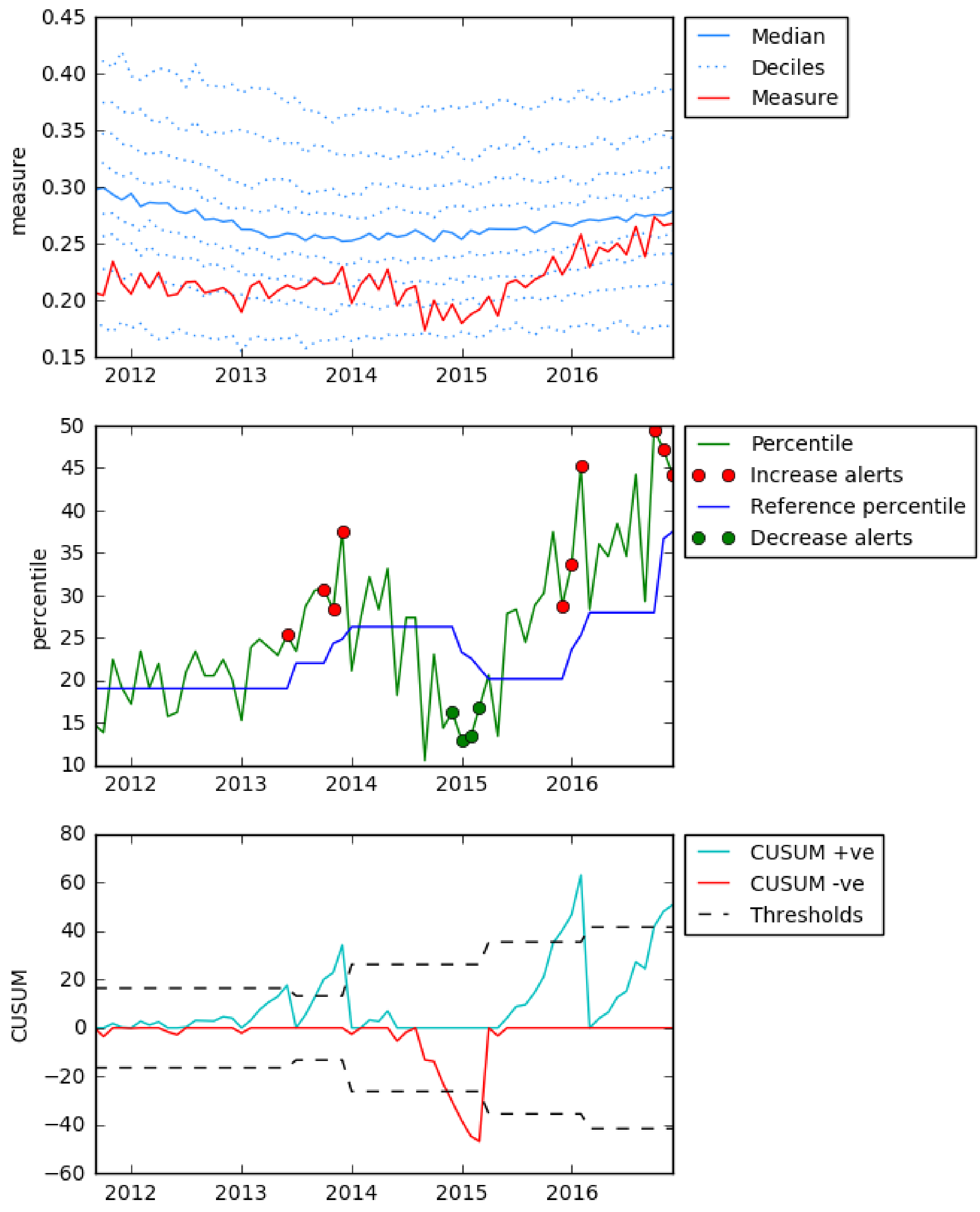

Measure: https://openprescribing.net/practice/G85138/#glaucoma

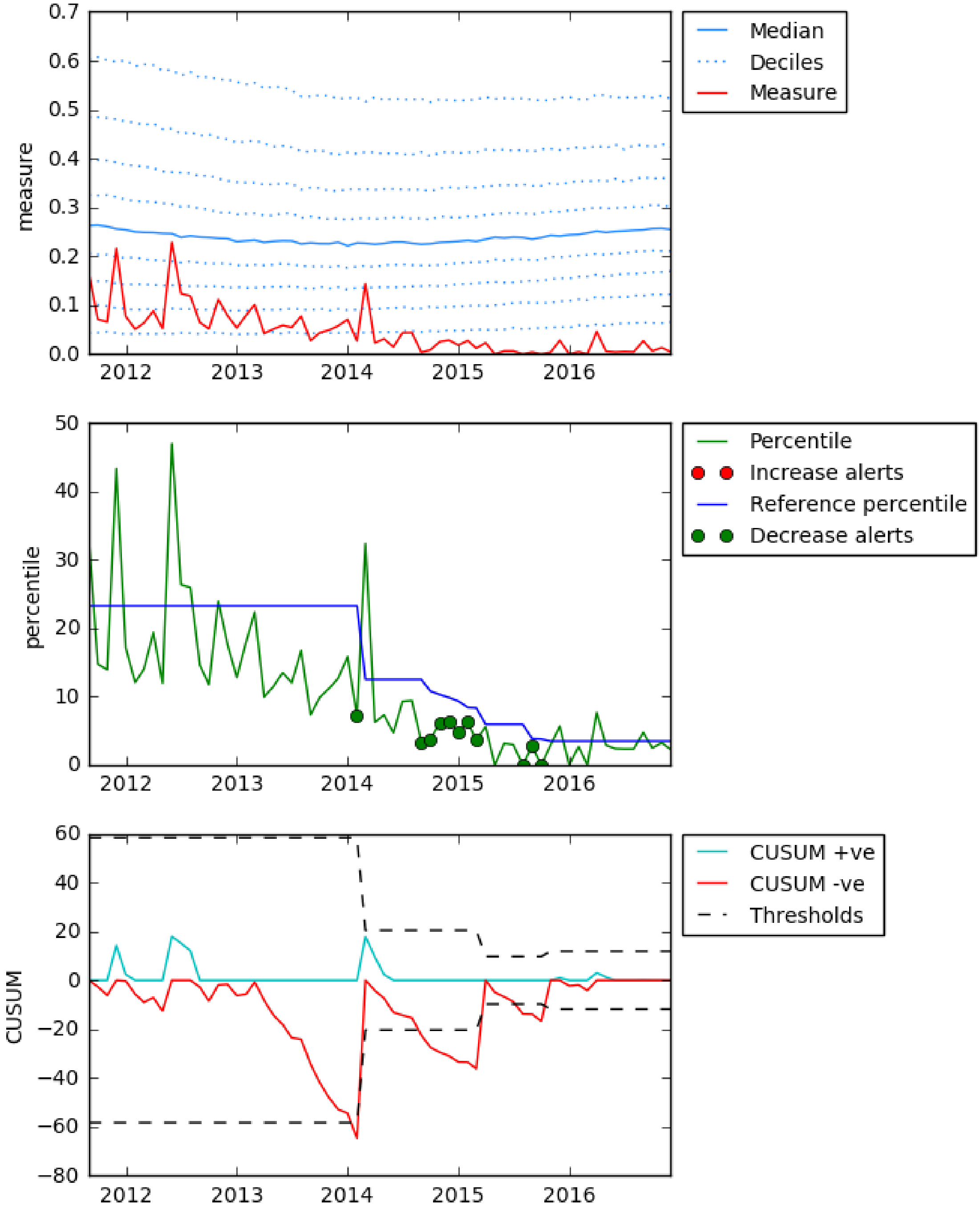

Measure: https://openprescribing.net/ccg/05Y/#ppidose

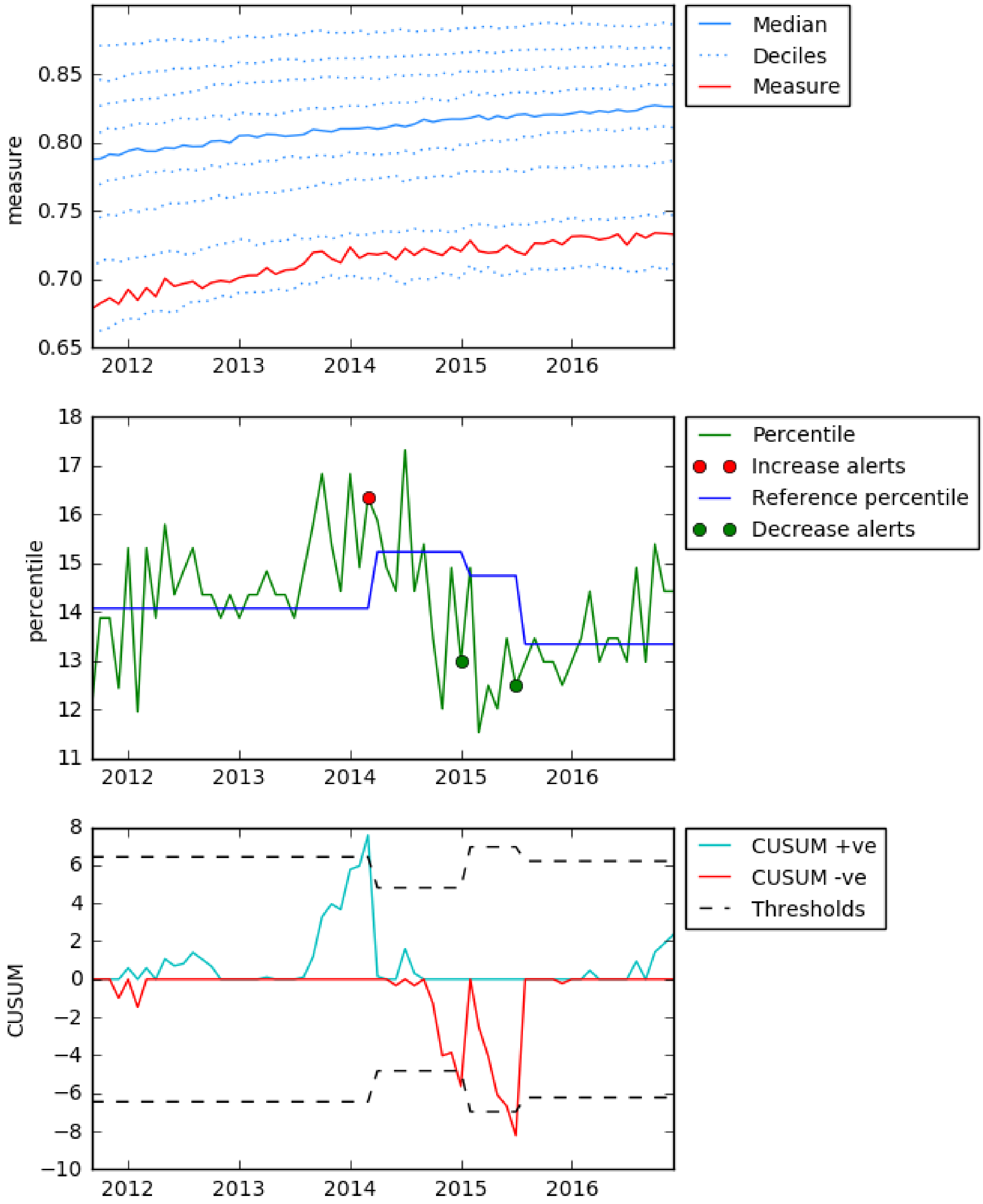

Measure: https://openprescribing.net/practice/G85138/#ppidose

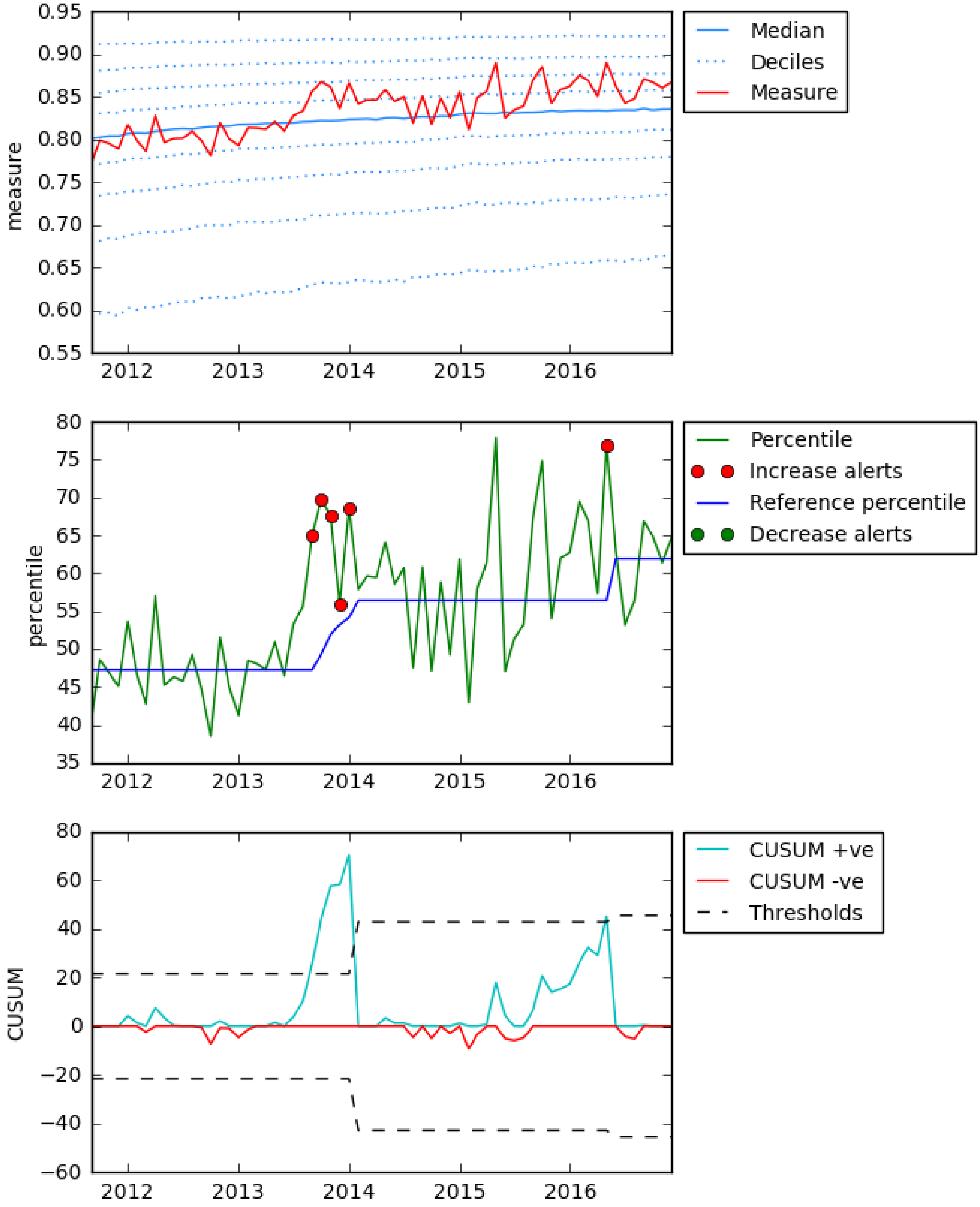

Measure: https://openprescribing.net/ccg/05Y/#ktt9_cephalosporins

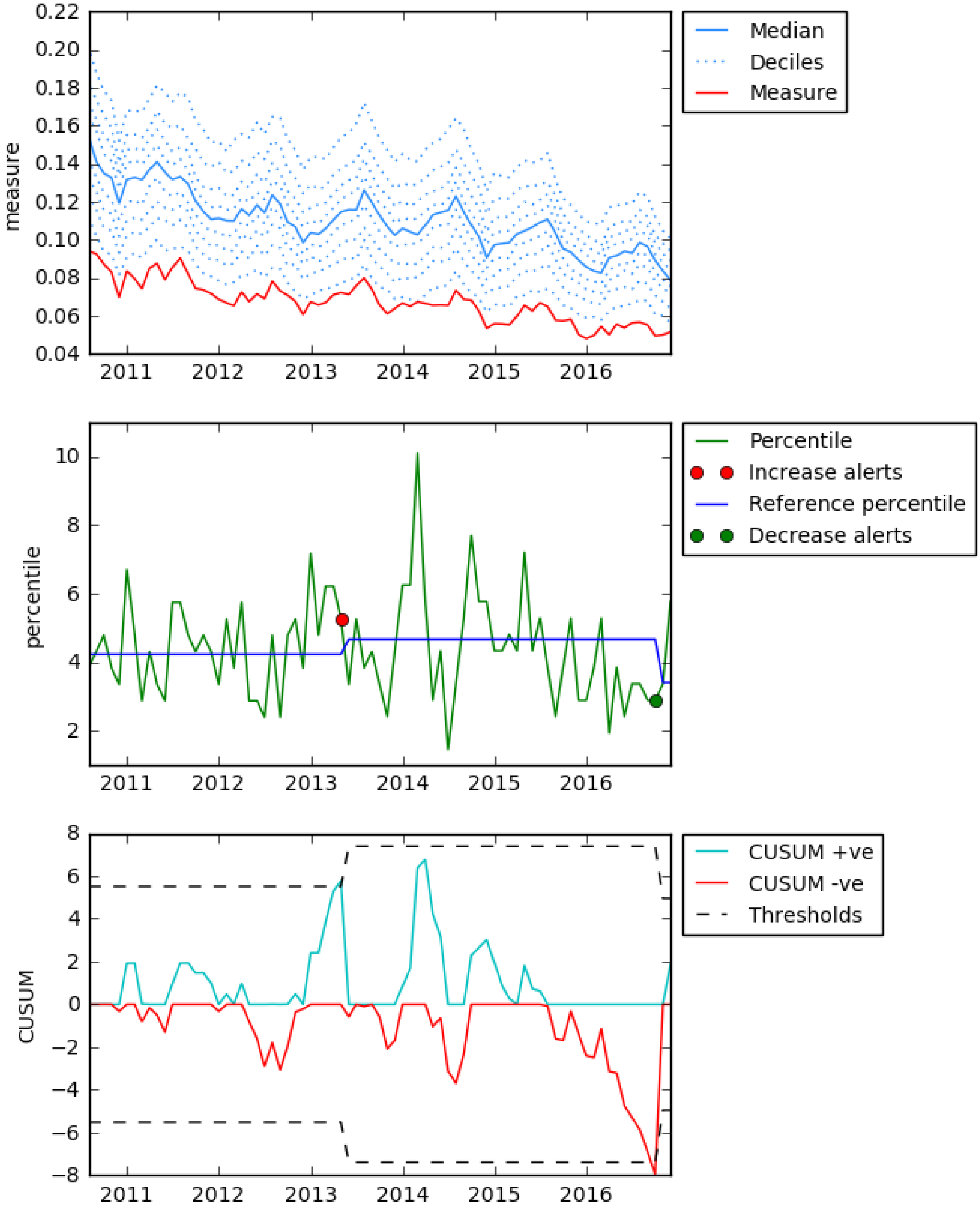

Measure: https://openprescribing.net/practice/G85138/#ktt9_cephalosporins

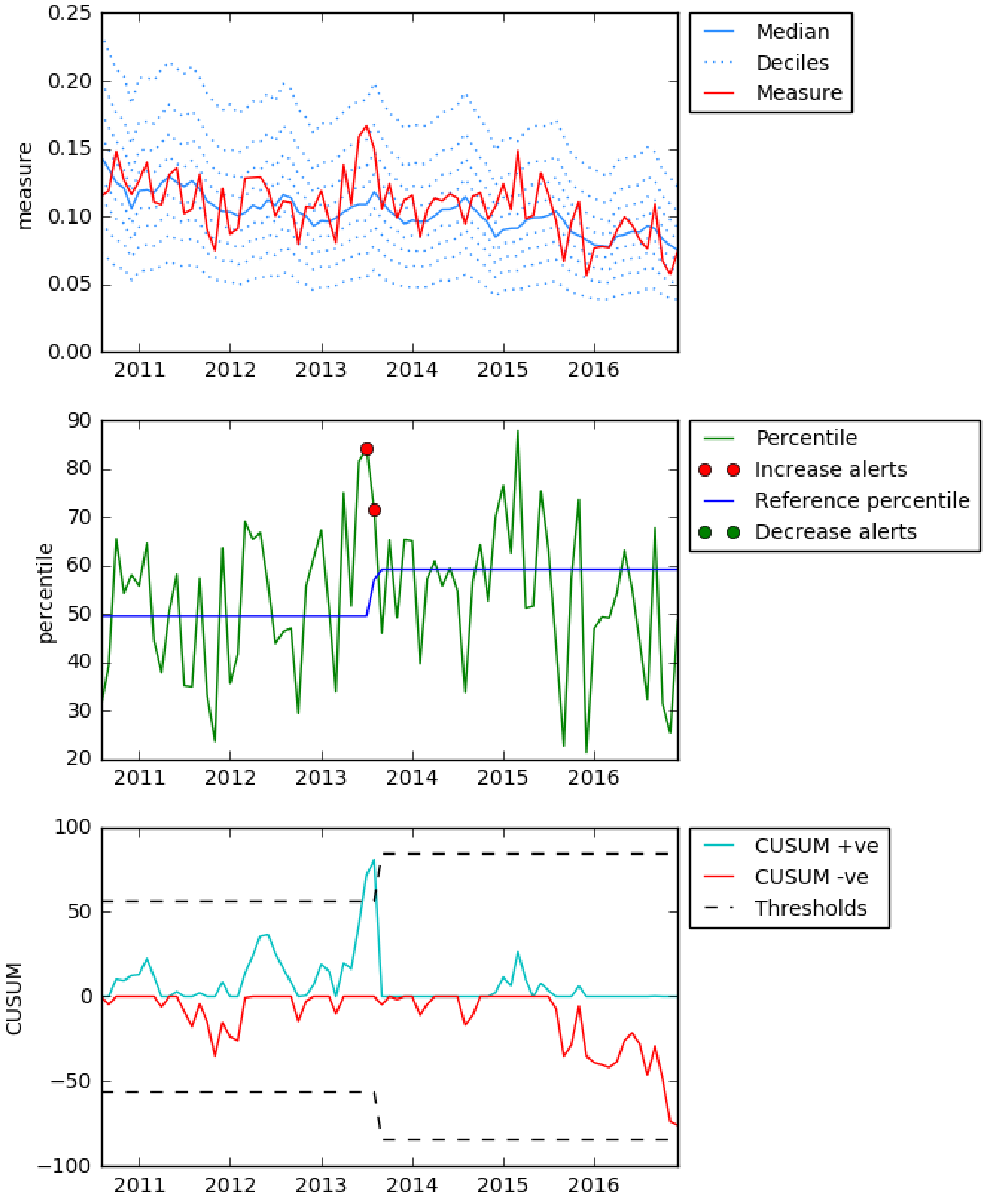

Measure: https://openprescribing.net/ccg/05Y/#quetiapine

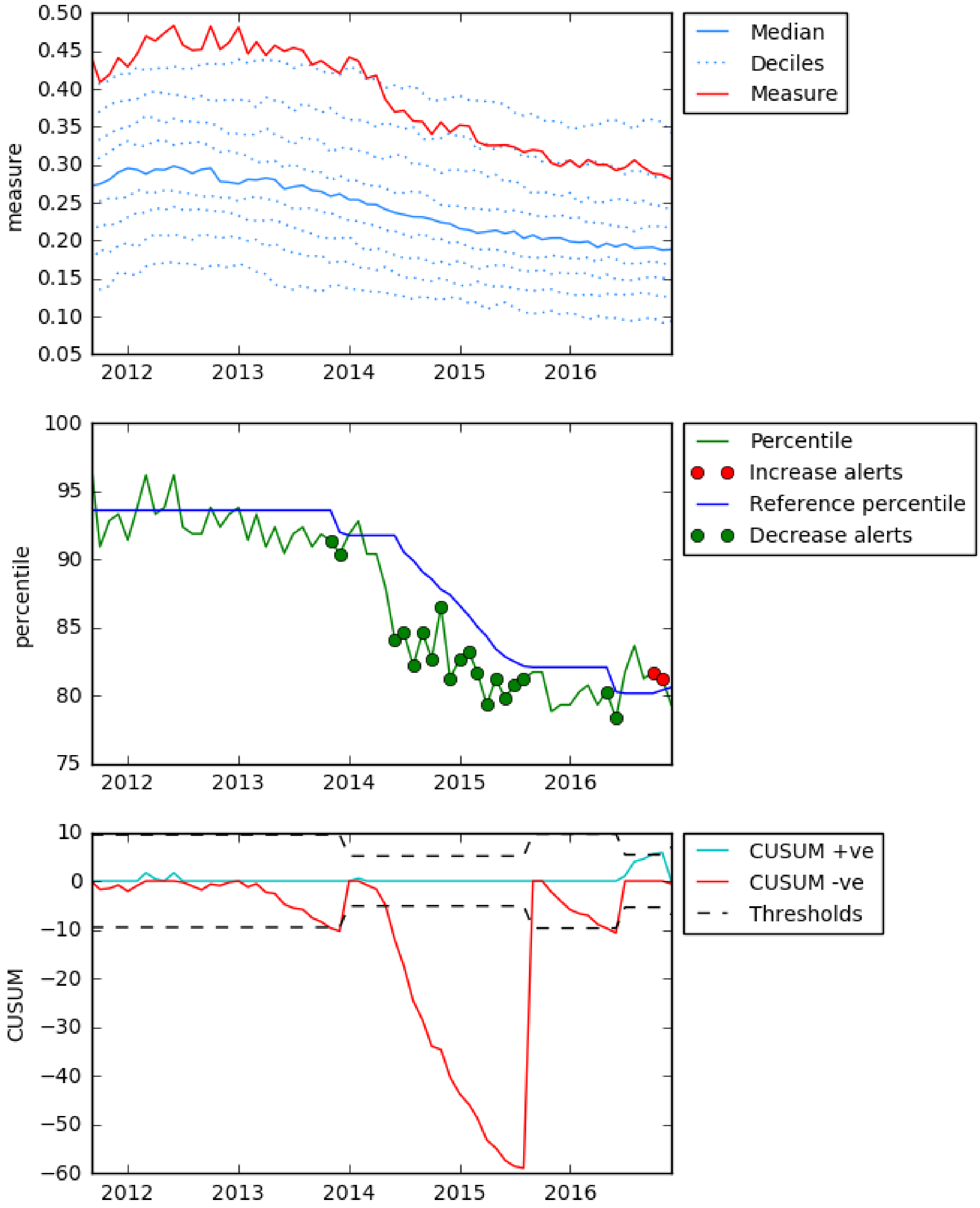

Measure: https://openprescribing.net/practice/G85138/#quetiapine

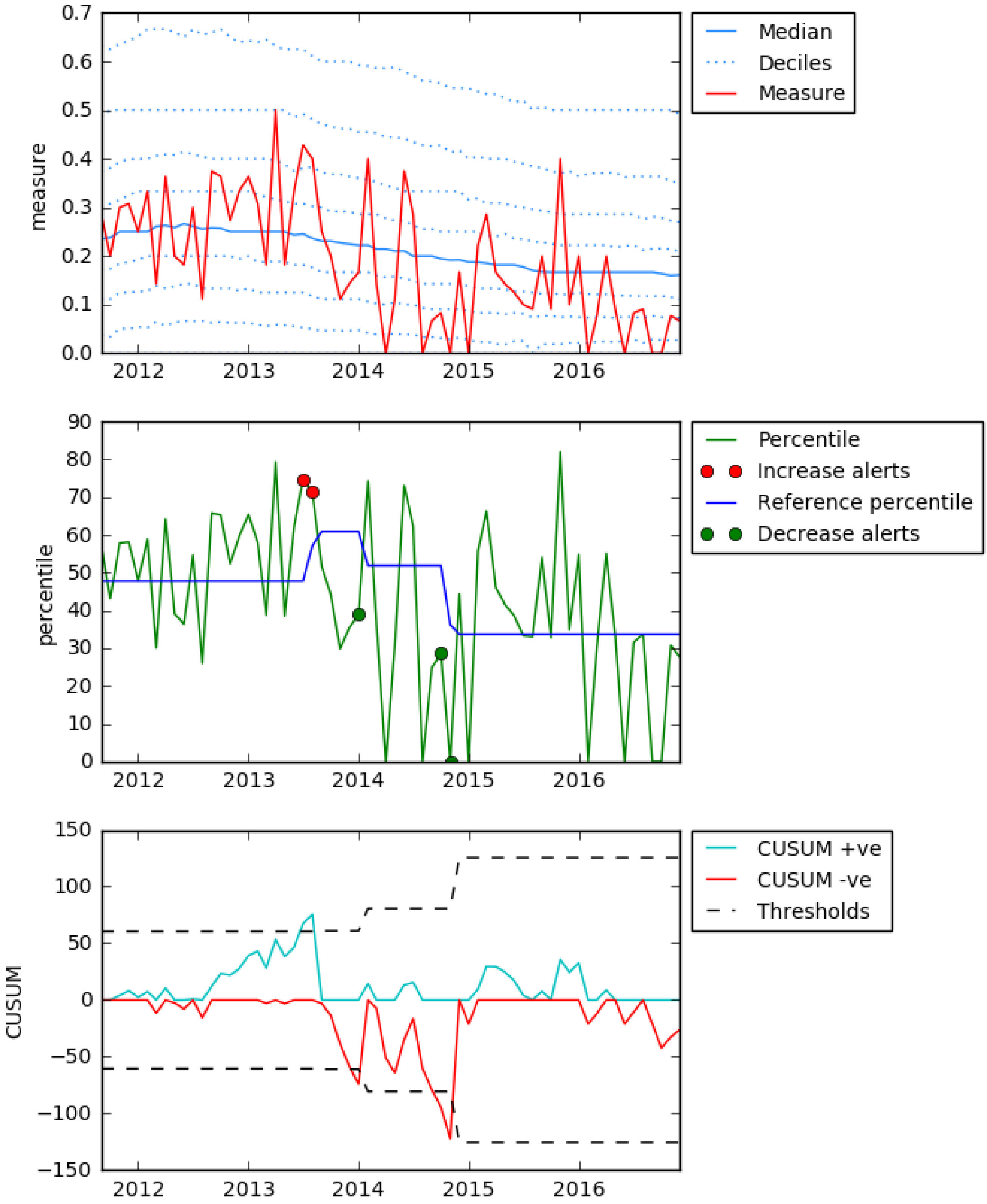

Measure: https://openprescribing.net/ccg/05Y/#saba

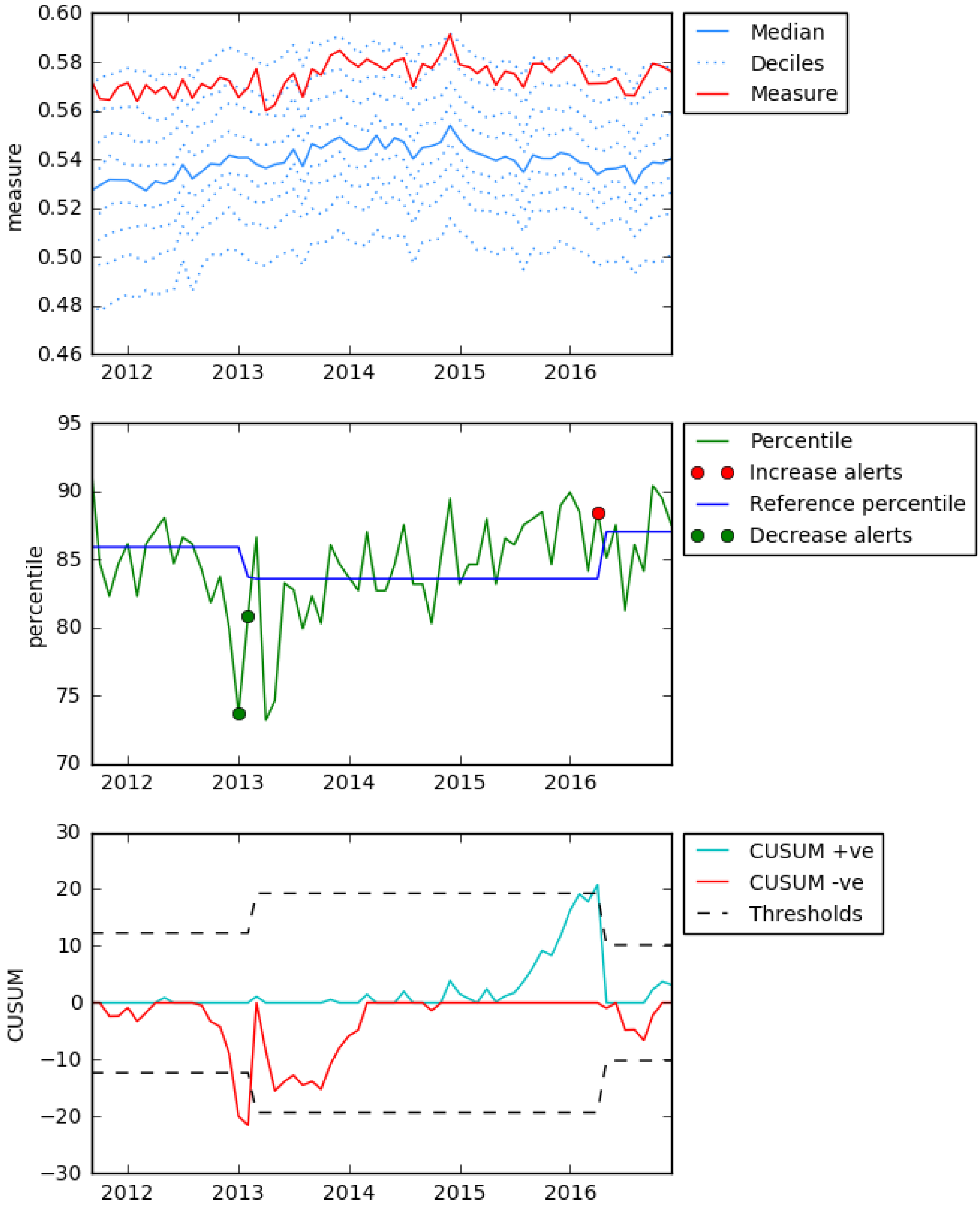

Measure: https://openprescribing.net/practice/G85138/#saba

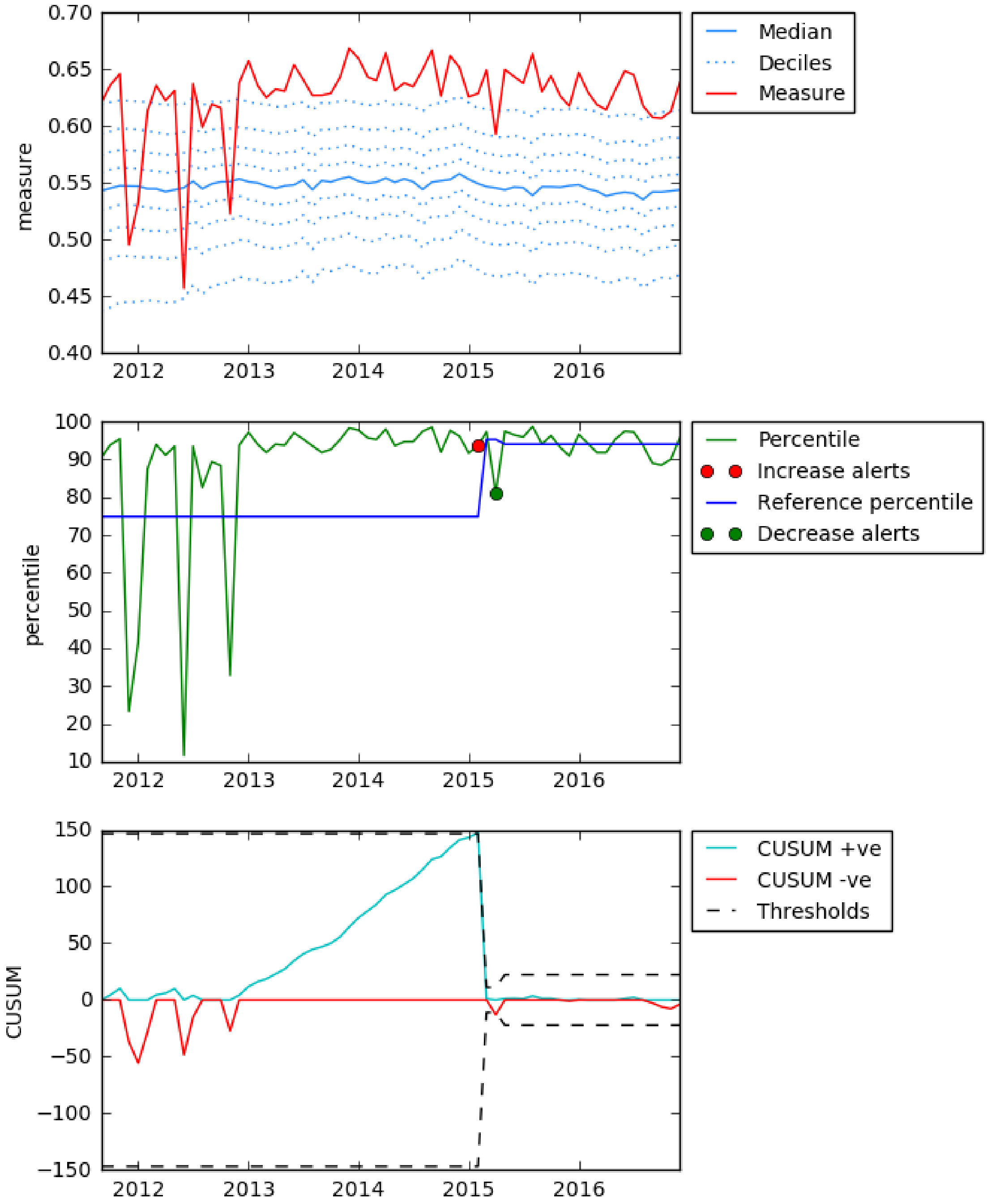

Measure: https://openprescribing.net/ccg/05Y/#keppra

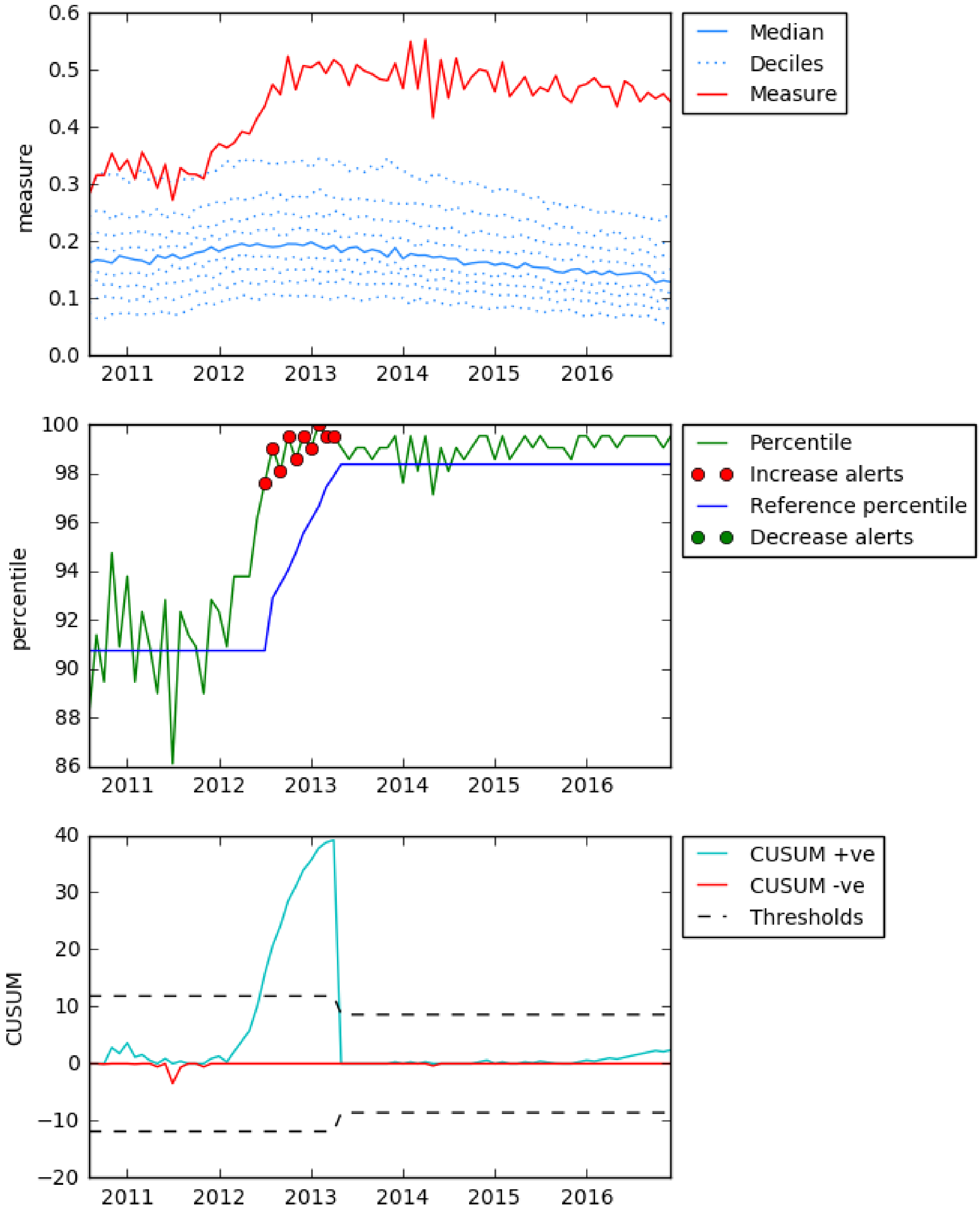

Measure: https://openprescribing.net/practice/G85138/#keppra

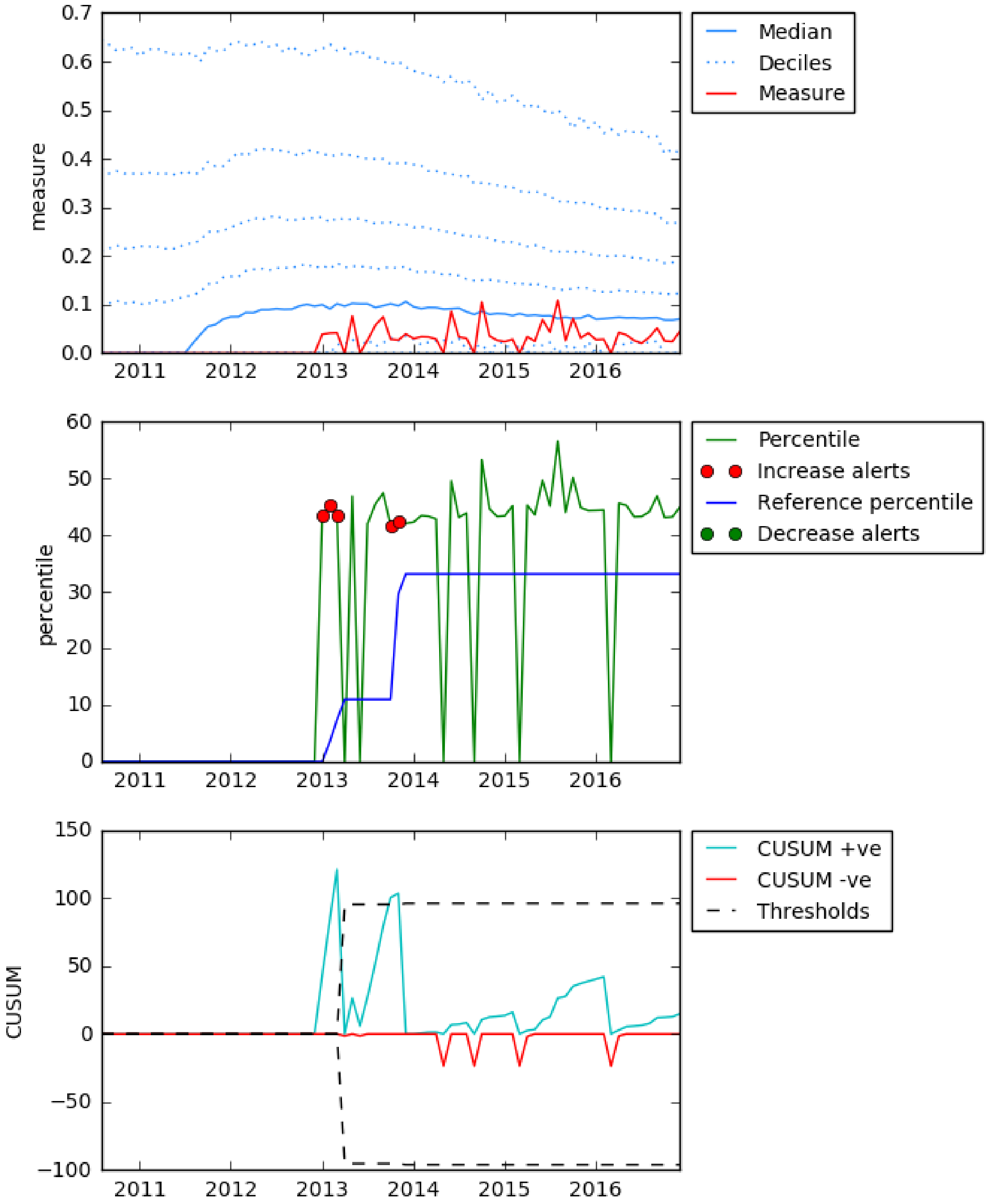

Measure: https://openprescribing.net/ccg/05Y/#silver

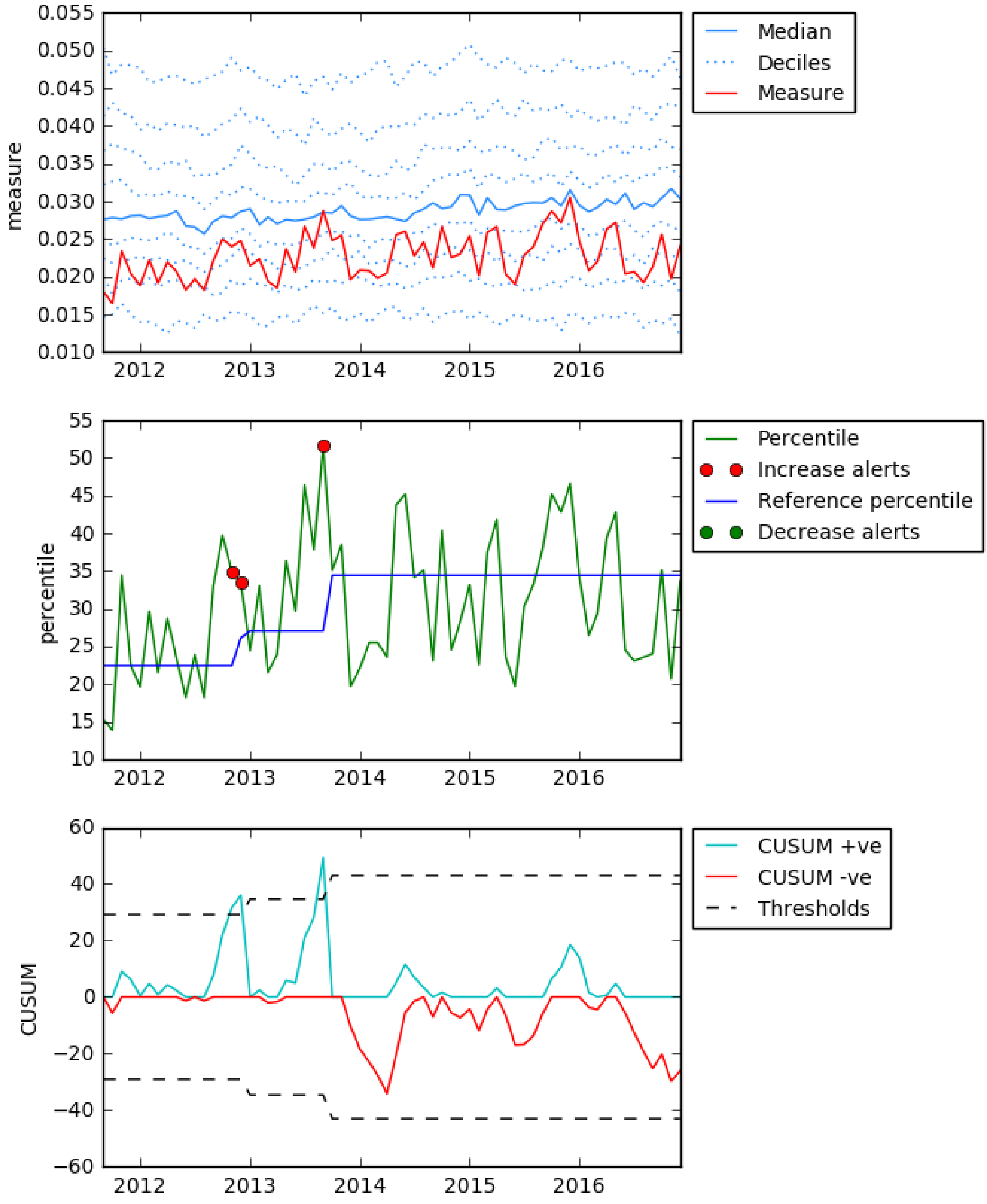

Measure: https://openprescribing.net/practice/G85138/#silver

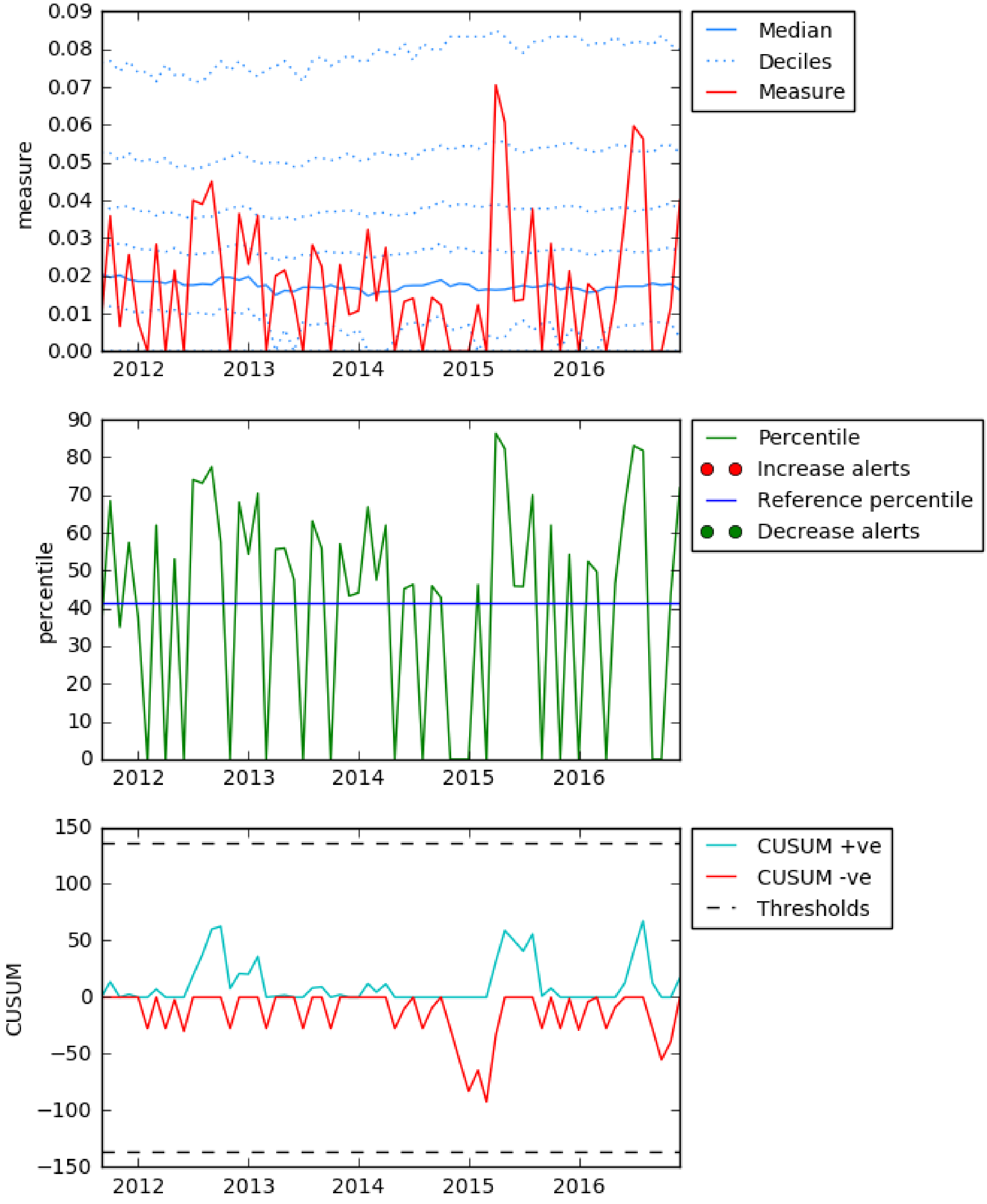

Supplement: Supplementary file 1 — Appendix A. Code. Appendix B. Example graphs. (PDF 4946 kb) [file 12911_2018_642_MOESM1_ESM.pdf]
